# Adherence to Physical Activity and Incident Mobility Disability in Older Adults With Mobility Limitations

**DOI:** 10.1002/jcsm.13870

**Published:** 2025-06-18

**Authors:** Alejandro Álvarez‐Bustos, Helio José Coelho‐Junior, Riccardo Calvani, Leocadio Rodriguez‐Mañas, Matteo Tosato, Matteo Cesari, Antonio Cherubini, Alfonso J. Cruz‐Jentoft, Pálmi V. Jónsson, Fabrizia Lattanzio, Marcello Maggio, Regina Roller‐Wirnsberger, Ingrid Rýznarová, Annemie M. W. J. Schols, Cornel C. Sieber, Alan J. Sinclair, Anna Skalska, Timo Strandberg, Achille Tchalla, Eva Topinková, Bruno Vellas, Stephan von Haehling, Francesco Landi, Emanuele Marzetti, for the SPRINTT consortium

**Affiliations:** ^1^ Biomedical Research Center Network for Frailty and Healthy Ageing (CIBERFES) Institute of Health Carlos III Madrid Spain; ^2^ Department of Geriatrics, Orthopaedics and Rheumatology Università Cattolica del Sacro Cuore Rome Italy; ^3^ Instituto de Investigación IdiPaz Madrid Spain; ^4^ Fondazione Policlinico Universitario Agostino Gemelli, IRCCS Rome Italy; ^5^ Department of Geriatrics Hospital Universitario de Getafe Madrid Spain; ^6^ Department of Clinical Sciences and Community Health University of Milan Milan Italy; ^7^ Geriatria, Accettazione Geriatrica e Centro di Ricerca per l'Invecchiamento IRCCS INRCA Ancona Italy; ^8^ Department of Clinical and Molecular Sciences Università Politecnica delle Marche Ancona Italy; ^9^ Servicio de Geriatría Hospital Universitario Ramón y Cajal‐IRYCIS Madrid Spain; ^10^ Department of Geriatrics, Landspitali University Hospital, Faculty of Medicine University of Iceland Reykjavik Iceland; ^11^ Department of Medicine and Surgery Università degli Studi di Parma Parma Italy; ^12^ Cognitive and Motor Centre, Medicine and Geriatric Rehabilitation Department of Parma University Hospital of Parma Parma Italy; ^13^ Department of Internal Medicine Medical University of Graz Graz Austria; ^14^ Silesian Hospital in Opava Opava Czech Republic; ^15^ Department of Respiratory Medicine, Research Institute NUTRIM Maastricht University Medical Centre Maastricht The Netherlands; ^16^ Institute for Biomedicine of Aging Friedrich‐Alexander‐Universität Erlangen‐Nürnberg Nurnberg Germany; ^17^ DROP and King's College London UK; ^18^ Department of Internal Medicine and Gerontology, Faculty of Medicine Uniwersytet Jagiellonski Collegium Medicum Krakow Poland; ^19^ Helsinki University and Helsinki University Hospital Helsinki Finland; ^20^ Center for Life‐Course Health Research University of Oulu Oulu Finland; ^21^ CHU Limoges, Department of Geriatric Medicine, UR 24134 – VieSante Research Unit (Aging, Frailty, Prevention, eHealth) Limoges France; ^22^ Department of Geriatrics, First Faculty of Medicine Charles University and General Faculty Hospital Prague Czech Republic; ^23^ Faculty of Health and Social Sciences University of South Bohemia Ceske Budejovice Czech Republic; ^24^ Gérontopôle Centre Hospitalier Universitaire de Toulouse Toulouse France; ^25^ IHU HealthAge, CHU, University of Toulouse, INSERM CERPOP Toulouse France; ^26^ Department of Cardiology and Pneumology University Göttingen Medical Center Göttingen Germany; ^27^ DZHK (German Centre for Cardiovascular Research), Partner Site Lower Saxony Göttingen Germany

**Keywords:** disability, frailty, frequency, physical activity, sarcopenia

## Abstract

**Background:**

Preservation of mobility independence is a primary goal in older adults with physical frailty and sarcopenia (PF&S). Interventions based on the combination of physical activity (PA) and nutritional counselling have been indicated as strategies for the management of this condition, although their effectiveness is not confirmed in all investigations. A possible explanation for this uncertain scenario relies in the impact of the adherence to PA interventions. Hence, the present study investigated the impact of the adherence to PA sessions on the incidence of mobility disability in older adults with PF&S.

**Methods:**

This is a secondary analysis of an evaluator blinded, randomised controlled trial, developed in 16 clinical sites across 11 European countries, from January 2016 to 31 October 2019. Participants were community‐dwelling older adults (70+ years) with PF&S enrolled in the SPRINTT trial (NCT02582138). PF&S was operationalised as having a total score from 3 to 9 on the short physical performance battery (SPPB), low appendicular lean mass and ability to complete the 400‐m walk test in < 15 min. Data from participants allocated to a multicomponent intervention (PA with technological support plus nutritional counselling) and a healthy ageing lifestyle education programme (control group) were analysed. Adherence to PA was assessed based on the number of weekly sessions attended. According to recommendations of the American College of Sports Medicine, adherence was categorised as below recommendations (< 2 sessions/week, BR), meeting recommendations (2–3 sessions/week, MR), and above recommendations (> 3 sessions/week, AR). The primary outcome was incident mobility disability, operationalised as incident inability to complete the 400‐m walk test in < 15 min during up to 36 months of follow‐up.

**Results:**

Data of 1444 participants (mean age 79.3 years, 72.6% women) were analysed. In those with SPPB scores of 3–7, MR and AR groups had lower risk of mobility disability compared with controls [MR HR (95% CI): 0.57 (0.41–0.78), *p* = 0.001; AR HR (95% CI): 0.33 (0.23–0.46), *p* < 0.001] and BR groups [MR: HR (95% CI): 0.48 (0.34–0.69), *p* < 0.001; AR: HR (95% CI): 0.27 (0.18–0.38), *p* < 0.001] in a dose‐dependent manner. In those with SPPB scores of 8 or 9, the BR group had a higher risk of mobility disability than controls. MR and AR groups had a lower risk of mobility disability than the BR group.

**Conclusions:**

In older adults with PF&S, adherence to PA recommendations is associated with lower incidence of mobility disability. This benefit depends on the degree of adherence as well as baseline physical performance.

**Trial Registration:** ClinicalTrials.gov NCT02582138

## 1 Introduction

Although life expectancy has increased steadily over the last decades, gains in disability‐free life years have been much smaller than those in lifespan [1, 2]. This scenario is, at least partly, attributable to limitations of existing models of care whose disease‐centric approach is not suitable for fostering active ageing [3]. Furthermore, insufficient resources are allocated to promote the transition to health systems that ensure availability of cost‐effective services prioritising the needs of older adults [2, 4]. Unfortunately, impactful conditions such as frailty and sarcopenia are often neglected in daily clinical practice.

Physical activity (PA) programmes and nutritional interventions are effective for preventing mobility disability in older adults with mobility limitations [5, 6]. Nevertheless, considerable heterogeneity in the response to interventions has been observed among study participants, with some individuals experiencing no benefits in mobility [5, 6]. A possible explanation for this scenario relies in the different levels of adherence to interventions, participant functional status or a combination of both. International health organisations recommend a minimum frequency of PA to achieve significant health benefits. For instance, the World Health Organisation (WHO) [7] and the American College of Sports and Medicine (ACSM) [8] encourage the practice of PA and exercise at least two to three times weekly in older adults who aim to improve or maintain cardiorespiratory, musculoskeletal and neuromotor health, as well as reducing the risk of falls, with greater gains possibly obtained with more weekly sessions [7, 8, 9]. However, no empirical evidence is available to support these assumptions in older adults with functional limitations.

The results of the ‘Sarcopenia and Physical fRailty IN older people: multi‐componenT Treatment strategies’ (SPRINTT) trial were recently published and indicated that a multicomponent intervention involving PA with technological support and nutritional counselling was effective in reducing the incidence of mobility disability in older adults with physical frailty and sarcopenia (PF&S) [6]. The average adherence of participants to the PA programme was approximately 70%, but significant variations in PA adherence were observed for both centre‐based (standard deviation 22.8%) and home‐based sessions (standard deviation 36.5%) [6]. However, the impact of attendance on the prevention of mobility disability was not explored.

The purpose of this study was to examine the effects of various levels of adherence to the PA programme on the risk of developing mobility disability in older adults with PF&S enrolled in the SPRINTT trial.

## 2 Methods

This study is a secondary analysis of the SPRINTT clinical trial (ClinicalTrials.gov identifier: NCT02582138) [6]. SPRINTT was a multicentre, evaluator‐blinded, randomised controlled trial (RCT) performed from 11 January 2016 to 31 October 2019 that tested whether a multicomponent intervention, encompassing PA with technological support and nutritional counselling, would reduce the risk of mobility disability in older adults with P&S compared with a healthy ageing lifestyle educational programme. Details on the RCT methodology and main findings are reported elsewhere [6, 10]. The ethics committee of the Università Cattolica del Sacro Cuore (Rome, Italy; coordinating centre) granted approval to the study protocol (protocol # 15611/15), which was then ratified by the ethics committees of all participating institutions. All candidate participants provided written informed consent before being evaluated for inclusion.

### 2.1 Participants

Trial participants were men and women aged 70 years or older with PF&S. The condition of interest was operationalised as the combination of (1) physical frailty, defined as a summary score on the short physical performance battery (SPPB) [11] of 3–9 points; (2) low appendicular lean mass (aLM), either absolute or body mass index (BMI) adjusted, according to sex‐specific cut‐points recommended by the Foundation for the National Institutes of Health (FNIH) sarcopenia project [12]; and (3) the absence of mobility disability, operationalised as the ability to complete a 400‐m walk test in less than 15 min without sitting, stopping for more than 1 min, help of another person, or using a walker [13]. The main criteria for exclusion were self‐reported walking disability, cognitive impairment (defined as a mini‐mental state examination [MMSE] [14], score < 24/30), having a terminal illness, engagement in a structured PA programme, contraindications to safely participate in the trial as determined by local study physicians, and plans to move out of the study area within at least 2 years. The primary trial population included participants with an SPPB score of 3–7 (*n* = 1205). An exploratory sample of 314 participants with an SPPB score of 8 or 9 was enrolled to describe the condition of interest and gather information on the impact of interventions over the entire range of SPPB scores defining physical frailty (i.e., 3–9).

Participants were randomised 1:1 to the multicomponent intervention or the healthy ageing lifestyle education programme using a web‐based randomisation system with permuted block algorithm, stratified by study site, sex, and SPPB score category (3–7 and 8 or 9).

### 2.2 Multicomponent Intervention and Adherence to PA

The multicomponent intervention was implemented for a maximum length of 36 months, which varied based on the timing of participant recruitment along the trial. The PA programme was modelled on the ‘Lifestyle Interventions and Independence for Elders’ (LIFE) study protocol [15] and comprised aerobic, strength, flexibility, and balance exercises. The workout routine was designed to be performed both at the study site and at home. Participants were expected to exercise at the centre twice weekly under the guidance of instructors. Centre‐based sessions were gradually complemented by home‐based exercises (up to four times weekly). Adherence was assessed by registering attendance at the centre and via the completion of diaries by participants documenting the frequency of home‐based sessions. For the purposes of the present study, adherence to PA sessions, both at centre and at home, was categorised according to ACSM recommendations as below (< 2 sessions weekly, BR), meeting (2–3 sessions weekly, MR), and above recommendations (> 3 sessions weekly, AR) [8].

The total amount of PA was measured continuously for 7 days at baseline and every 6 months thereafter using the activPAL3 actimeter (PAL Technologies Ltd., Glasgow, UK) placed on the thigh. Instructors could request extra 7‐day actimetry recordings if there were signs that participants were not adhering to target prescriptions. Instructors utilised the information to offer personalised feedback to participants on their performance objectives, enhance adherence, and eliminate potential disincentives. The dietary component was devised to enhance the impact of the PA programme. The intervention included personalised nutritional evaluations and prescription of dietary plans aimed at achieving a daily energy intake of 25–30 kcal/kg body weight and a daily protein intake of at least 1.0–1.2 g/kg body weight.

### 2.3 Healthy Ageing Lifestyle Educational Programme

The healthy ageing lifestyle educational programme was carried out for up to 36 months and consisted of lectures and workshops on topics relevant to older adults. Activities were conducted in groups of 10–20 participants once or twice a month, with a minimum attendance requirement of once a month. Sessions ended with a short programme (5–10 min) of upper extremity stretching exercises or some relaxation techniques.

### 2.4 Outcome

The primary outcome measure was mobility disability, which was operationalised as the inability to complete the 400‐m walk test in less than 15 min without sitting, halting for more than 1 min, or requiring the assistance of a walker or another person [13]. For the test, participants were asked to complete 10 circuits around a 20‐m course at their habitual pace without overexerting themselves. The 400‐m walk test was administered at baseline, at 3 months, and every 6 months after randomisation. Mobility disability was considered ascertained at the date the participant: (a) failed the 400‐m walk test, (b) attended a scheduled visit and was found unable to attempt the test, or (c) died. A rigorous procedure was developed for outcome adjudication in the event the 400‐m walk test was not performed.

### 2.5 Study Variables

#### 2.5.1 SPPB

The SPPB is a standardised and objective tool used to measure lower extremity function in older adults [11]. The battery includes three tests: the balance test, the gait speed test, and the chair stand test. For the balance test, participants were requested to stand in three increasingly challenging positions for up to 10 s each: side‐by‐side stand, semi‐tandem stand, and full tandem stand. For the gait speed test, participants were instructed to walk at their usual speed over a 4‐m track. The faster of two trials (m/s) was used for the scoring. Finally, for the chair stand test, participants were instructed to fold their arms across their chest and stand up from a straight‐backed chair with no armrests five times as quickly as possible. The time (in seconds) was used for scoring the test. Each test was assigned a score ranging from 0 (*inability to complete the test*) to 4 (*best performance*) to obtain a summary score spanning from 0 (*worst performance*) to 12 (*best performance*).

#### 2.5.2 Isometric Handgrip Strength

Handgrip strength was measured using a Jamar (J. A. Preston Corporation, Clifton, NJ, USA) or a North Coast handheld dynamometer (North Coast Medical Inc., Morgan Hill, CA, USA). The measurement was taken when the test person was sat on a chair with the shoulder in a neutral position, the elbow close to the body and bent at a 90° angle, and the wrist in a neutral position (thumbs up). The contralateral stayed relaxed beneath the thigh. The dominant hand was tested unless the participant reported pain in the wrist or hand or had undergone fusion, arthroplasty, tendon repair, synovectomy, or other surgery of the hand or wrist of the dominant side in the past 3 months. One practice try was allowed to familiarise participant with the feel of the instrument. Afterward, two trials of maximal contraction under encouragement were performed with a 10‐s rest interval. The higher reading (in kg) was recorded.

#### 2.5.3 aLM

aLM was measured by whole‐body dual‐energy x‐ray absorptiometry (DXA) on a Hologic (Bedford, MA, USA) or a GE Lunar (General Electric, Boston, MA, USA) scanner. Instruments were cross‐calibrated using a calibration block phantom. As a further means of standardisation, DXA scan raw files were transferred to the coordinating centre in Rome for centralised analysis. Low aLM was operationalised according to two alternative sex‐specific criteria proposed by the FNIH initiative: BMI‐adjusted aLM < 0.789 in men and < 0.512 in women or absolute aLM < 19.75 kg in men and < 15.02 kg in women [12].

### 2.6 Covariables

The MMSE [14] and the Strength, Assistance with walking, Rising from a chair, Climbing stairs and Falls (SARC‐F) [16] were used to assess global cognitive function and the probability of sarcopenia, respectively. The Center for Epidemiological Studies Depression Scale (CES‐D) [17], a 20‐item self‐rating questionnaire, was employed as a screening tool for depressive symptoms. Energy and protein intake was estimated based on information collected by 3‐day dietary records using standard nutrition software. Finally, the presence of medical conditions, including Type 2 diabetes mellitus, cardiovascular disease, osteoarthritis, and cancer, was obtained through self‐report and clinical records if available.

### 2.7 Statistical Analysis

Data are presented as mean ± standard deviation (SD) for continuous variables, and frequency (percentages) for categorical variables. Analyses were conducted according to SPPB score categories (3–7 and 8 or 9) and adherence to PA sessions. Differences in participant characteristics according to their adherence to the PA programme and group allocation were examined via analysis of variance (ANOVA) and χ^2^ statistics for continuous and categorical variables, respectively. Cox proportional hazard regression models were conducted to evaluate associations of different levels of PA adherence with incident mobility disability. The final model included age, sex, BMI, medical conditions, SPPB, MMSE, CES‐D, and SARC‐F. Two‐way ANOVA was conducted to examine changes in physical performance tests and aLM that could be associated with the effects of adherence to PA sessions on the incidence of mobility disability. Adherence categories and time (i.e., baseline, 24 months and 36 months) served as independent variables, whereas changes in SPPB, aLM, aLM/BMI, time to complete the 400‐m walk test, handgrip strength, time to complete the chair stand test and 4‐m walk speed were analysed as dependent variables. If significance was observed in adherence × time interaction, Bonferroni's post hoc test was conducted to identify differences among groups. Effect sizes and 95% confidence intervals (95% CIs) were calculated for all analyses, and the level of significance was set at *p* < 0.05.

All analyses were performed using SPSS (Version 23; IBM, New York, NY, USA) and STATA (Version 16; StataCorp LLC, College Station, TX, USA) statistical packages.

## 3 Results

### 3.1 Characteristics of Study Participants

**FIGURE 1:**
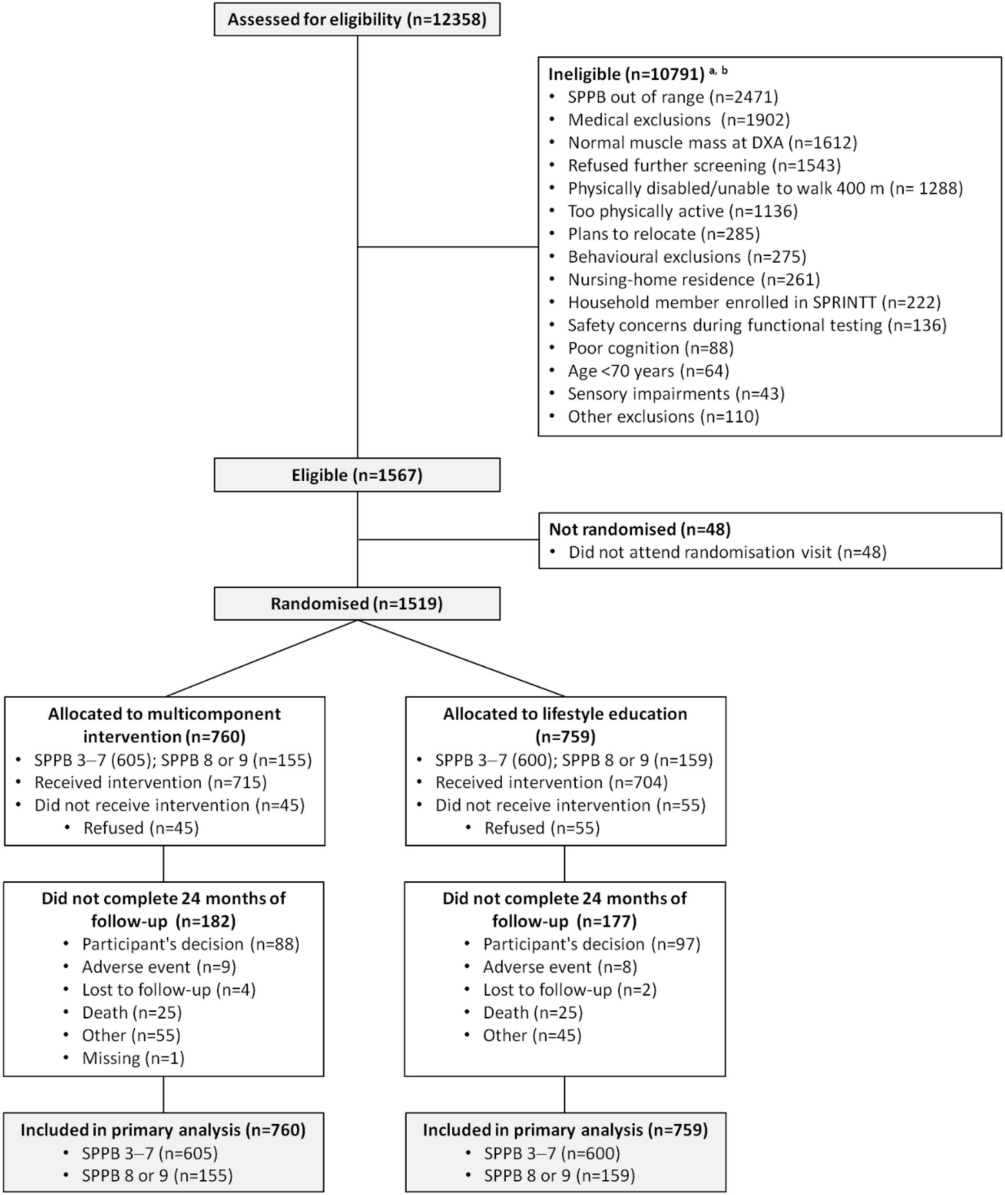
Flow of participants through the study. ^a^The sum of individual items is higher than the number of ineligible participants because the screening process was not always stopped at the first unmet eligibility criterion. ^b^Some entries are different from those previous published [18] because of data updates following database cleaning.

Of the 760 participants randomised to the multicomponent interventions, 45 declined to partake before the first PA session. Among the remaining 715 participants, 689 had complete data available for analysing their adherence to the PA programme, both at the centre and at home. Thus, data of a total of 1448 participants, including 759 allocated in the lifestyle education group, were analysed (Figure 1). The main characteristics of the 1448 participants according to SPPB score categories, group allocation and adherence to PA sessions are listed in Table 1. Of the whole sample, 1203 (mean age 79.2 ± 5.8 years; 72.0% women) had a baseline SPPB score of 3–7, whereas 316 (77.7 ± 5.6 years; 74.8% women) had a score of 8 or 9.

**TABLE 1:** Characteristics of study participants according to baseline SPPB score category, group allocation and attendance to physical activity sessions.

	SPPB 3–7							SPPB 8–9				
	Lifestyle education (*n* = 600)	Multicomponent intervention					Lifestyle education (*n* = 159)	Multicomponent intervention				
		Weekly physical activity sessions			Total(*n* = 554)						Total (*n* = 135)	
		< 2 (*n* = 288)	2–3 (*n* = 113)	> 3 (*n* = 153)		*p*		< 2 (*n* = 49)	2–3 (*n* = 35)	> 3 (*n* = 51)		*p*
Age, years	79.16 ± 5.7	79.4 ± 6.2	79.2 ± 5.5	79.1 ± 5.6	79.3 ± 5.9	0.903	77.1 ± 5.4	79.6 ± 6.3*	77.6 ± 5.4	77.5 ± 5.1	78.3 (5.7)	0.051
Sex (women)	425 (70.8)	204 (70.6)	83 (74.1)	112 (73.2)	399 (72.0)	0.889	117 (73.1)	40 (81.6)	23 (65.7)	38 (74.5)	101 (74.8)	0.424
BMI, kg/m^2^	28.7 ± 6.0	28.6 ± 5.4	27.9 ± 5.2	29.1 ± 4.82	28.6 ± 5.2	0.373	28.3 ± 6.1	27.6 ± 5.9	28.8 ± 5.6	28.6 ± 5.0	28.3 ± 5.5	0.787
Appendicular lean mass, kg												
Absolute appendicular lean mass, kg	16.6 ± 3.8	16.5 ± 3.9	16.3 ± 3.6	16.5 ± 3.7	16.5 ± 3.7	0.914	16.6 ± 4.6	15.8 ± 3.2	16.8 ± 3.4	16.3 ± 3.9	16.3 ± 3.5	0.635
BMI‐adjusted appendicular lean mass	0.585 ± 0.117	0.587 ± 0.119	0.590 ± 0.116	0.569 ± 0.111	0.582 ± 0.116	0.350	0.589 ± 0.117	0.587 ± 0.117	0.582 ± 0.142	0.573 ± 0.120	0.580 ± 0.124	0.870
SPPB score	6.2 ± 1.1	6.0 (1.2)	6.4 (0.9) **	6.4 (0.9) **	6.2 (1.1)	0.001	8.6 ± 0.5	8.5 (0.5)	8.5 (0.5)	8.7 (0.5)	8.6 (0.5)	0.127
400‐m walk test time, min	9.06 ± 2.5	9.3 ± 2.7	8.6 ± 2.1	8.8 ± 2.4	9.0 ± 2.5	0.037	7.27 ± 1.59	8.0 ± 2.2	7.7 ± 2.0	7.7 ± 2.3	7.8 ± 2.2	0.094
Chair stand test, s	19.2 ± 6.7	19.1 ± 6.0	20.7 ± 8.9	18.3 ± 6.4***	19.2 ± 6.8	0.043	19.4 ± 6.2	18.6 ± 4.3	18.5 ± 5.3	18.2 ± 5.0	18.4 ± 4.8	0.542
4‐m gait speed, s	6.14 ± 1.70	6.46 ± 1.91*	5.87 ± 1.35**	5.85 ± 1.38**	6.17 ± 1.70	0.001	4.8 ± 1.1	4.89 ± 0.89	4.92 ± 1.01	4.97 ± 1.01	4.93 ± 0.96	0.883
Handgrip strength, kg	20.4 ± 8.9	19.9 ± 8.3	19.5 ± 8.0	19.5 ± 8.6	19.7 ± 8.3	0.595	20.5 ± 9.1	20.4 ± 7.4	21.4 ± 7.0	21.3 ± 9.6	21.0 ± 8.2	0.880
SARC‐F score	3.1 ± 2.0	3.2 ± 1.8	3.1 ± 1.8	2.9 ± 1.7	3.1 ± 1.8	0.598	2.3 ± 1.7	1.8 ± 1.4	2.8 ± 1.5**	2.2 ± 1.8	2.2 ± 1.6	0.078
MMSE score	27.9 ± 1.8	27.8 ± 1.8	27.7 ± 1.8	28.3 ± 1.7**	27.9 ± 1.8	0.032	28.3 ± 1.8	27.9 ± 2.0	28.1 ± 1.7	28.1 ± 1.7	28.0 ± 1.8	0.452
CES‐D score	5.6 ± 3.9	6.0 ± 3.8	5.5 ± 4.2	5.3 ± 4.2	5.7 ± 4.0	0.285	5.1 ± 3.8	4.8 ± 4.2	5.9 ± 4.2	5.4 ± 4.2	5.3 ± 4.2	0.679
Cardiovascular disease	422 (70.5)	219 (75.8)	81 (72.3)	106 (69.3)	406 (73.3)	0.361	101 (63.1)	34 (69.4)	26 (74.3)	39 (76.5)	99 (73.3)	0.252
Diabetes mellitus	139 (23.2)	64 (22.2)	27 (24.1)	31 (20.3)	122 (22.0)	0.865	30 (18.8)	6 (12.2)	6 (17.1)	6 (11.8)	18 (13.3)	0.555
Cancer	82 (13.7)	40 (13.8)	16 (14.3)	14 (9.2)	70 (12.6)	0.470	25 (15.6%)	10 (20.4)	5 (14.3)	8 (15.7)	23 (17.0)	0.855
Osteoarthritis	462 (77.1)	208 (72.0)	89 (79.5)	128 (83.7)	425 (76.7)	0.036	118 (73.8)	38 (77.6)	24 (68.6)	41 (80.4)	103 (76.3)	0.602
Follow‐up length, months	26.0 ± 8.7	23.4 ± 10.2*	28.0 ± 6.6**	28.8 ± 6.2 * ^,^ **	25.8 ± 8.9	< 0.001	29.0 ± 10.7	25.0 ± 11.9	33.2 ± 5.4**	32.8 ± 4.2**	30.1 ± 8.9	< 0.001
Incident disability	315 (52.6)	170 (58.8)	46 (41.1)	43 (28.1)	259 (46.8)	< 0.001	39 (24.4)	19 (38.8)	11 (31.4)	9 (17.7)	39 (28. 9)	0.084

*Note:* Data are shown as mean ± standard deviation and absolute numbers (%).

Abbreviations: BMI = body mass index, CES‐D = Center for Epidemiologic Studies Depression Scale, MMSE = mini mental state examination, SARC‐F = Strength, Assistance with walking, Rising from a chair, Climbing stairs and Falls, SPPB = short physical performance battery.

* *p* < 0.05 versus lifestyle education.

** *p* < 0.05 versus < 2 sessions weekly.

*** *p* < 0.05 versus 2–3 sessions weekly.

In the SPPB 3–7 group, 289 (52.2%) had a PA adherence below ACSM recommendations, 113 (20.4%) met the ACSM recommendations, and 153 (27.6%) attended more than three sessions weekly. Participants with higher adherence to PA sessions (i.e., MR and AR) had higher SPPB scores and better walking speed performance than those in the BR category. Moreover, AR had higher MMSE scores in comparison to the BR group and better performance on the chair stand test compared with the MR group. Significant differences in the rate of osteoarthritis and the incidence of disability were observed among groups. No other significant differences were found.

In the SPPB 8 or 9 group, 49 (36.3%) had a PA adherence below ACSM recommendations, 35 (25.9%) met the recommendations, and 51 (37.8%) attended more than three sessions a week. Participants who exercised at least twice weekly had longer follow‐up than the BR group. No other significant differences were observed.

### 3.2 Incidence of Mobility Disability According to Adherence to PA Sessions

Incident mobility disability was experienced by 574 (49.7%) participants with SPPB of 3–7 group, and of 78 (26.5%) in the SPPB 8 or 9 group. The risk of incident mobility disability in the two SPPB score groups is shown in Table 2. In the SPPB 3–7 score category, participants in the MR (HR: 0.57 95% CI: 0.41–0.78; *p* = 0.001) and AR (HR: 0.33; 95% CI: 0.23–0.46; *p* < 0.001) groups had a lower risk of mobility disability compared with those in the lifestyle education programme. Moreover, MR and AR had a significantly lower risk of incident mobility disability than BR. When the two groups with higher adherence were compared, a significantly lower risk of incident mobility disability was observed in AR participants (HR: 0.50; 95% CI: 0.32–0.79; *p* = 0.003).

**TABLE 2:** Probability of incident mobility disability according to baseline SPPB score category.

SPPB 3–7												
	Model 1						Model 2					
	HR (95% CI)	*p*	HR (95% CI)	*p*	HR (95% CI)	*p*	HR (95% CI)	*p*	HR (95% CI)	*p*	HR (95% CI)	*p*
Lifestyle education	Reference						Reference					
Physical activity sessions												
< 2 weekly	1.27 (1.05–1.53)	0.013	Reference				1.13 (0.93–1.38)	0.214	Reference			
2–3 weekly	0.58 (0.43–0.79)	0.001	0.46 (0.33–0.63)	< 0.001	Reference		0.56 (0.41–0.77)	< 0.001	0.49 (0.35–0.69)	< 0.001	Reference	
> 3 weekly	0.36 (0.26–0.50)	< 0.001	0.28 (0.20–0.39)	< 0.001	0.60 (0.40–0.91)	0.016	0.34 (0.25–0.48)	< 0.001	0.28 (0.20–0.40)	< 0.001	0.54 (0.35–0.83)	0.005
Age							1.07 (1.05–1.08)	< 0.001	1.07 (1.05–1.10)	< 0.001	1.09 (1.05–1.14)	< 0.001
Sex							0.77 (0.64–0.94)	0.009	0.75 (0.56–1.00)	0.050	0.83 (0.49–1.39)	0.472
BMI							1.02 (1.01–1.04)	0.005	1.01 (0.98–1.04)	0.458	1.03 (0.99–1.08)	0.140
Diabetes mellitus							1.08 (0.88–1.33)	0.450	1.11 (0.8–1.52)	0.533	0.75 (0.43–1.30)	0.303
Cardiovascular disease							1.24 (1.01–1.51)	0.039	1.48 (1.07–2.04)	0.018	1.74 (1.01–3.00)	0.046
Osteoarthritis							0.98 (0.80–1.20)	0.845	1.11 (0.80–1.55)	0.523	1.28 (0.69–2.40)	0.434
Cancer							1.00 (0.78–1.28)	0.997	0.97 (0.66–1.41)	0.871	1.11 (0.60–2.06)	0.737
SPPB score							0.87 (0.80–0.94)	< 0.001	0.84 (0.75–0.94)	0.003	0.79 (0.63–0.98)	0.031
MMSE score							1.00 (0.95–1.05)	1.000	1.03 (0.96–1.11)	0.406	1.07 (0.94–1.21)	0.302
CES‐D score							1.04 (1.01–1.06)	0.001	1.00 (0.97–1.03)	0.984	1.00 (0.95–1.06)	0.871
SARC‐F score							1.13 (1.07–1.18)	< 0.001	1.11 (1.03–1.21)	0.008	0.95 (0.83–1.09)	0.485

SPPB 8–9												
	Model 1						Model 2					
	HR (95% CI)	*p*	HR (95% CI)	*p*	HR (95% CI)	*p*	HR (95% CI)	*p*	HR (95% CI)	*p*	HR (95% CI)	*p*
Lifestyle education	Reference						Reference					
Physical activity sessions												
< 2 weekly	2.32 (1.33–4.03)	0.003	Reference				2.82 (1.49–5.33)	0.001	Reference			
2–3 weekly	1.01 (0.52–1.98)	0.973	0.45 (0.21–0.94)	0.034	Reference		1.07 (0.53–2.15)	0.846	0.29 (0.12–0.69)	0.006	Reference	
> 3 weekly	0.54 (0.26–1.12)	0.100	0.24 (0.11–0.54)	< 0.001	0.56 (0.23–1.37)	0.205	0.57 (0.27–1.19)	0.133	0.16 (0.07–0.39)	< 0.001	0.64 (0.25–1.67)	0.365
Age							1.04 (0.99–1.09)	0.105	0.99 (0.93–1.05)	0.739	0.93 (0.83–1.04)	0.189
Sex							0.51 (0.30–0.88)	0.015	0.46 (0.21–1.00)	0.051	0.34 (0.12–0.97)	0.043
BMI							1.01 (0.97–1.06)	0.595	1.00 (0.93–1.06)	0.917	1.02 (0.93–1.12)	0.681
Diabetes mellitus							1.75 (0.94–3.25)	0.077	2.72 (1.04–7.15)	0.042	3.14 (0.90–10.91)	0.072
Cardiovascular disease							0.65 (0.38–1.11)	0.118	0.48 (0.22–1.06)	0.068	0.39 (0.13–1.20)	0.101
Osteoarthritis							0.78 (0.45–1.37)	0.388	0.74 (0.33–1.66)	0.466	0.58 (0.18–1.81)	0.344
Cancer							0.81 (0.42–1.57)	0.537	0.57 (0.21–1.53)	0.264	0.66 (0.14–3.10)	0.600
SPPB score							1.03 (0.62–1.70)	0.919	1.83 (0.90–3.74)	0.096	1.33 (0.44–4.05)	0.617
MMSE score							0.93 (0.82–1.05)	0.231	0.99 (0.82–1.21)	0.932	0.83 (0.59–1.18)	0.297
CES‐D score							1.06 (1.00–1.12)	0.054	1.04 (0.96–1.13)	0.369	1.00 (0.90–1.12)	0.950
SARC‐F score							1.13 (0.97–1.32)	0.124	1.15 (0.92–1.43)	0.236	1.22 (0.91–1.64)	0.179

*Note:* Model 1: unadjusted model. Model 2: adjusted for age, sex, BMI, medical conditions, SPPB, MMSE, CES‐D, and SARC‐F.

Abbreviations: BMI = body mass index, CES‐D = Center for Epidemiologic Studies Depression Scale, CI = confidence interval, HR = hazard ratio, MMSE = mini mental state examination, SARC‐F = Strength, Assistance with walking, Rising from a chair, Climbing stairs and Falls, SPPB = short physical performance battery.

In the SPPB 8 or 9 score category, participants in the BR group had a significantly higher risk of mobility disability than those in the lifestyle education programme (HR: 2.48; 95% CI: 1.27–4.86, *p* = 0.008). MR (HR: 0.32; 95% CI: 0.13–0.79; *p* = 0.014) and AR (HR: 0.19; 95% CI: 0.08–0.48; *p* < 0.001) groups had a significantly lower likelihood of mobility disability compared with the BR group. No other significant differences were observed.

Results were consistent when death was removed from the primary outcome (Table S1).

### 3.3 Changes in Physical Performance and aLM by Adherence to PA Sessions

**FIGURE 2:**
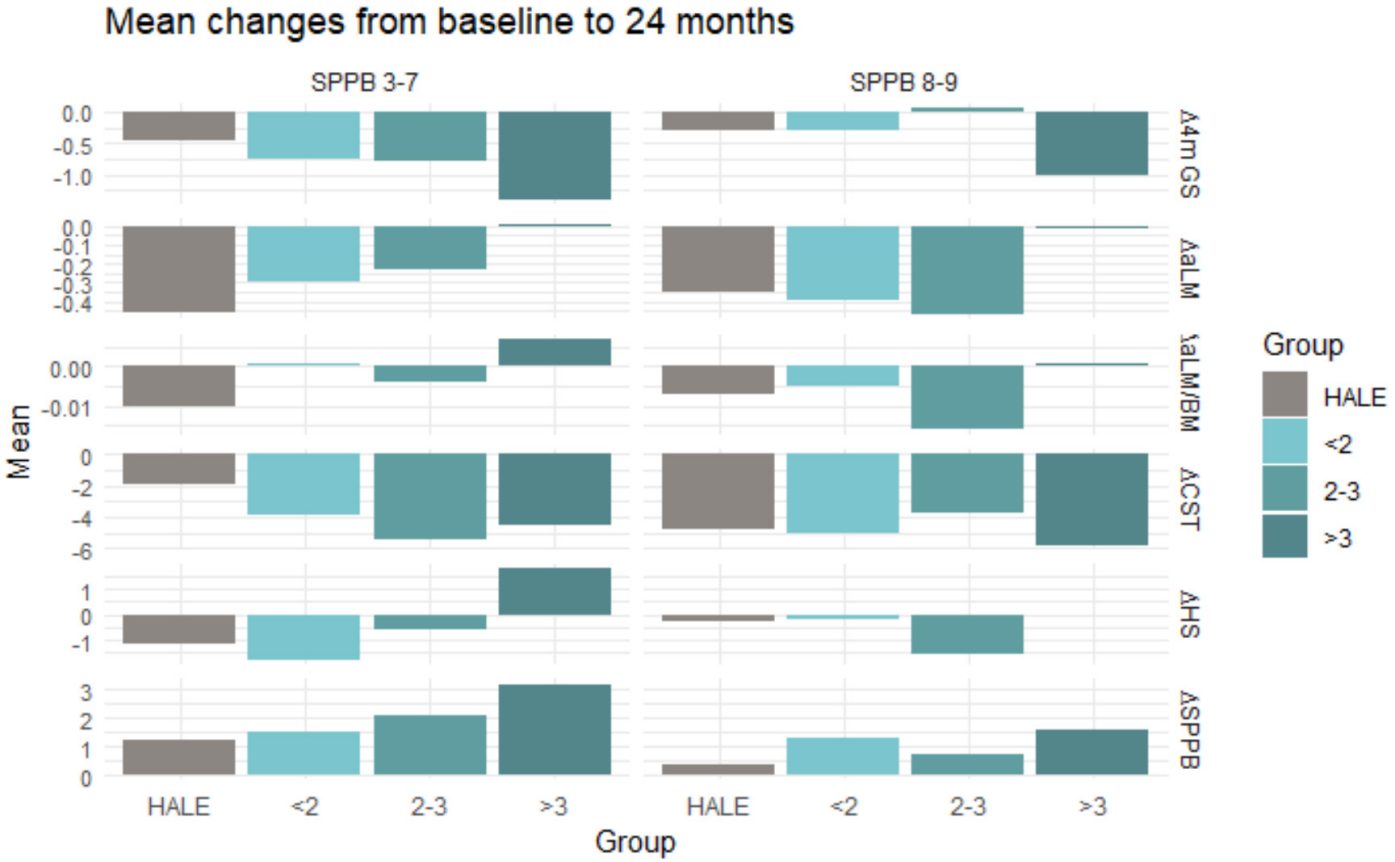
Changes in physical performance and appendicular lean mass based on adherence to physical activity sessions over 24 months. < 2, 2–3, > 3 = multicomponent intervention group categorisation according to weekly physical activity adherence; 4‐m GS = 4‐m gait speed, aLM = appendicular lean mass, BMI = body mass index, CST = chair stand test, HALE = healthy ageing lifestyle education, HS = handgrip strength, SPPB = short physical performance battery.

**FIGURE 3:**
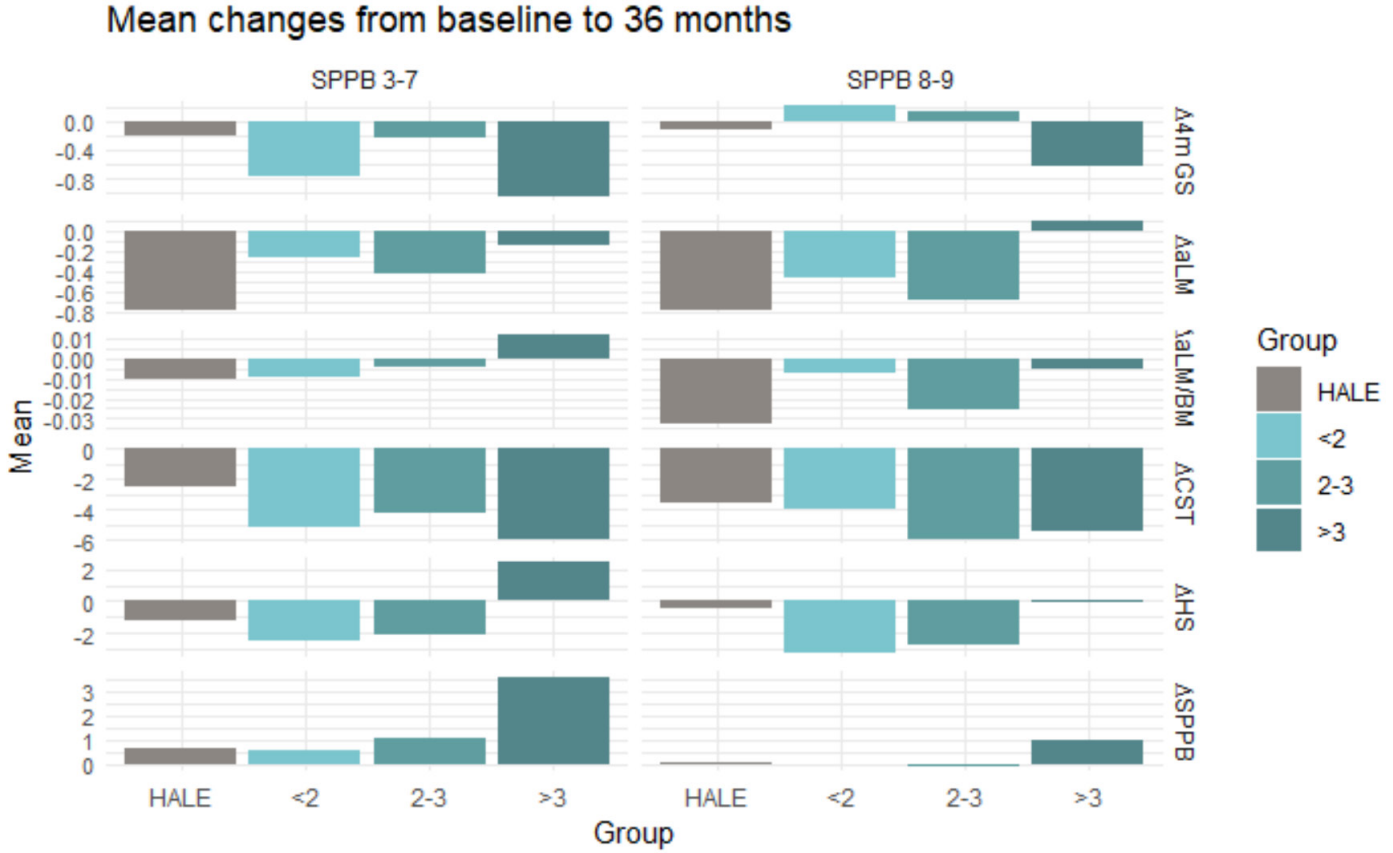
Changes in physical performance and appendicular lean mass based on adherence to physical activity sessions over 36 months. < 2, 2–3, > 3 = multicomponent intervention group categorisation according to weekly physical activity adherence; 4‐m GS = 4‐m gait speed, aLM = appendicular lean mass, BMI = body mass index, CST = chair stand test, HALE = healthy ageing lifestyle education, HS = handgrip strength, SPPB = short physical performance battery.

Changes in physical performance and aLM according to intervention allocation, SPPB score categories and adherence to PA sessions are shown in Table 3. At 24 months, AR participants with SPPB scores of 3–7 experienced greater improvements in SPPB, aLM, handgrip strength and 4‐m gait speed than those in the BR and lifestyle education groups (Figure 2). A greater increase in handgrip strength was also observed in participants who attended more than three PA sessions weekly compared with the MR group. At 36 months, AR participants had greater improvements in SPPB and handgrip strength than those in the MR and BR groups (Figure 3).

**TABLE 3:** Mean changes from baseline (∆) to 24 and 36 months in physical performance and appendicular lean mass according to group allocation, SPPB score category, and adherence to physical activity.

Variable	Time	SPPB 3–7					SPPB 8–9				
		Lifestyle education	Adherence to physical activity sessions (weekly)				Lifestyle education	Adherence to physical activity sessions (weekly)			
			< 2	2–3	> 3	*p*		< 2	2–3	> 3	*p*
∆ SPPB score	24 months	1.2 (2.3)	1.5 (2.8)	2.1 (2.1)***	3.2 (1.8)* ^,^ ***	< 0.001	0.4 (1.8)	1.3 (1.9)	0.7 (2.0)	1.6 (1.6)*	0.001
	36 months	0.7 (2.8)	0.6 (2.9)	1.1 (2.8)	3.6 (2.0)*,**,***	< 0.001	0.1 (1.9)	0.0 (3.3)	−0.1 (3.0)	1.0 (2.9)	0.504
∆ aLM (kg)	24 months	−0.46 (1.03)	−0.30 (0.94)	−0.23 (0.80)	0.01 (0.88)*,***	0.001	−0.35 (0.75)	−0.39 (0.97)	−0.47 (0.69)	−0.01 (0.99)	0.086
	36 months	−0.79 (1.04)	−0.26 (1.05)	−0.42 (1.25)	−0.15 (0.75)	0.041	−0.84 (0.90)	−0.47 (0.98)	−0.69 (1.05)	0.10 (1.15)*	0.008
∆ aLM/BMI	24 months	−0.01 (0.056)	0.001 (0.038)	−0.004 (0.044)	0.007 (0.049)***	0.028	−0.007 (0.043)	−0.005 (0.042)	−0.016 (0.040)	0.001 (0.035)	0.462
	36 months	−0.01 (0.046)	−0.009 (0.041)	−0.004 (0.053)	0.012 (0.042)***	0.049	−0.033 (0.050)	−0.007 (0.034)	−0.026 (0.058)	−0.005 (0.046)	0.152
∆ Handgrip strength (kg)	24 months	−1.2 (6.1)	−1.8 (4.7)	−0.6 (6.6)	1.8 (6.5)*,**,***	< 0.001	−0.3 (5.2)	−0.2 (3.4)	−1.6 (3.8)	0.0 (4.6)	0.539
	36 months	−1.2 (6.2)	−2.4 (5.5)	−2.1 (4.4)	2.4 (7.5)*,**,***	0.012	−0.4 (6.8)	−3.2 (3.5)	−2.7 (7.3)	−0.1 (4.6)	0.262
∆ Chair stand test (s)	24 months	−1.9 (9.7)	−3.9 (8.6)	−5.4 (9.1)***	−4.6 (7.7)	0.007	−4.8 (7.2)	−5.1 (7.2)	−3.70 (7.9)	−5.8 (5.8)	0.672
	36 months	−2.5 (7.1)	−5.1 (8.7)	−4.2 (10.5)	−5.9 (6.9)	0.222	−3.5 (7.2)	−4.0 (7.6)	−5.9 (9.0)	−5.4 (5.6)	0.626
∆ 4‐m gait speed (m/s)	24 months	−0.46 (1.75)	−0.74 (2.69)	−0.79 (1.57)	−1.40 (1.49)*,***	< 0.001	−0.31 (1.09)	−0.29 (1.28)	0.05 (1.01)	−1.02 (1.15)*,**,***	< 0.001
	36 months	−0.19 (2.34)	−0.78 (1.96)	−0.22 (1.50)	−1.05 (3.03)	0.279	−0.11 (1.28)	0.21 (1.30)	0.14 (0.84)	−0.63 (1.17)	0.167

*Note:* Data are shown as mean (standard deviation).

Abbreviations: aLM = appendicular lean mass, BMI = body mass index, SPPB = short physical performance battery.

* *p* < 0.05 versus lifestyle education.

** *p* < 0.05 versus < 2 sessions weekly.

*** *p* < 0.05 versus 2–3 sessions weekly.

In the SPPB 8 or 9 score category, AR participants had greater improvements in SPPB score and 4‐m gait speed than the lifestyle education group at 24 months and greater gains in aLM at 36 months. Improvements in the 4‐m gait speed experienced by the AR group were greater than in the MR and BR groups. No other significant differences were observed.

The effects of adherence to PA sessions and time on physical performance and aLM are reported in Tables S2 and S3. In the SPPB 3–7 group, a significant adherence × time interaction was observed for ∆ SPPB [*F*(6, 1970) = 13.921, *p* < 0.001, partial ƞ^2^ = 0.041], ∆ aLM [*F*(6, 1861) = 384.575, *p* < 0.001, partial ƞ^2^ = 0.554], ∆ aLM/BMI [*F*(6, 1861) = 6.004, *p* < 0.001, partial ƞ^2^ = 0.019], ∆ 5STS [*F*(6, 1790) = 4.226, *p* < 0.001, partial ƞ^2^ = 0.014] and ∆ 4‐m gait speed [*F*(6, 1949) = 2.184, *p* 0.042, partial ƞ^2^ = 0.007]. In the SPPB 8 or 9 group, a significant adherence × time interaction was observed for ∆ SPPB [*F*(6, 622) = 2.446, *p* = 0.024, partial ƞ^2^ = 0.023], ∆ aLM [*F*(6, 583) = 128.938, *p* < 0.001, partial ƞ^2^ = 0.570], ∆ aLM/BMI [*F*(6, 583) = 3.190, *p* = 0.004, partial ƞ^2^ = 0.032] and ∆ 4‐m gait speed [*F*(6, 618) = 2.274, *p* = 0.035, partial ƞ^2^ = 0.022].

## 4 Discussion

The present study examined the effects of adherence to a PA programme carried out on a mixed basis (study centre and home) on the incidence of mobility disability in older adults with PF&S. Our findings indicate that participants who meet PA recommendations by the ACSM had a lower risk of mobility disability, regardless of the presence of mobility limitations at baseline identified according to SPPB scores. In participants with poorer baseline performance (i.e., SPPB 3–7), higher adherence rates were associated with lower risk of mobility disability. In those with SPPB of 8 or 9, the multicomponent intervention did not reduce the risk of incident mobility disability compared with lifestyle education, regardless of the level of adherence to PA.

Our results indicate that older adults with PF&S and mobility limitations (SPPB 3–7) need to exercise at least twice weekly to reduce the risk of developing mobility disability. In contrast, those with SPPB of 8 or 9 and low adherence to PA may experience a greater risk of incident mobility disability compared with participants receiving lifestyle education.

According to the general adaptation theory [19], the response of the human body to stressful events (e.g., PA) [20] depends on the frequency of the stimulus that provokes allostasis. A low exercise frequency may be insufficient to elicit physiological gains, whereas repetitive and exhaustive PA sessions may reduce the time available to properly recover, thereby blunting beneficial adaptations [21].

Findings of the present study might be explained by the reinterpretation of Haskell's model proposed by Brahms et al. [22], according to which the degree of increase in physical function resulting from an exercise intervention is inversely related to an individual's initial level of function. Indeed, in participants with baseline SPPB of 3–7, greater gains in aLM, walking speed and SPPB scores were observed according to their adherence to the PA programme. When participants with SPPB of 8 or 9 were analysed, only the AR group had significantly improvements in aLM, walking speed and SPPB scores.

Our results are in line with several other studies that reported greater gains in physical performance and muscle mass in older adults who performed more weekly PA sessions. In a secondary analysis of the LIFE study, Fielding et al. [23] found a dose‐dependent relationship between attendance to PA sessions and increases in SPPB scores and gait speed performance from baseline to 24 months of follow‐up. Authors also found that the greater the increase in PA the lower the risk of incident mobility disability over 24 months [23]. Farinatti et al. [24] compared the effects of different training frequencies on muscle strength and physical function in physically active old women. Results indicated that participants with higher training frequencies (3 days/week) had greater gains in muscle strength and mobility relative to those who exercised once weekly. Comparable findings were reported by Turpela et al. [25] who observed that a higher training frequency was associated with greater improvements in lower extremity muscle strength and mobility. Along similar lines, Padilha et al. [26] showed that old women who exercised three times a week for 12 weeks experienced greater gains in upper extremity muscle strength than those who performed fewer sessions.

The effects of exercise on muscle mass in older adults are well established, but little is known on the influence of training frequency. Most evidence has been obtained from sensitivity analyses of systematic reviews, with mixed results. Kneffel et al. [27] found no effects of training frequency on muscle hypertrophy in apparently healthy older adults. Similar results were reported by Benito et al. [28] and Schoenfeld et al. [29] in healthy adults. In contrast, Schoenfeld et al. [30] observed a greater effect size for muscle hypertrophy in those with higher training frequencies [30]. Differences among studies might be explained by participants characteristics (e.g., age), training status (e.g., physically inactive vs. trained) and exercise training protocols.

Our study has several strengths. First, the condition of interest (PF&S) received empirical validation through an RCT [6]. Second, participants whose data were used for the study were enrolled as part of a multicentre international trial and were followed up for up to 36 months. Third, the PA programme was previously shown to be feasible by frail older adults and effective at reducing the risk of incident mobility disability [6]. Fourth, all physical performance and body composition measures are well‐established metrics for the evaluation of older adults.

Some limitations of our study need to be acknowledged. First, only training frequency was analysed as a moderator. Hence, the effect of other exercise parameters, such as intensity or modality (aerobic, strengthening, balance and flexibility), should be explored in future studies. Furthermore, exercise intensity was adjusted using the rating of perceived exertion (RPE) method using a Borg scale. Although this approach is well validated and widely used, employing more precise adjustment methods, such as maximum repetitions for strength exercises, and relative maximum cardiorespiratory capacity (% of heart rate) for the aerobic component, could lead to more accurate adjustments and potentially greater improvements in mobility outcomes. Second, a more detailed analysis of weekly PA levels could provide a better appreciation of participant's adherence to the home‐based PA programme. Third, home‐based training frequency was obtained from self‐report, which is susceptible to bias [31]. Fourth, participants were able to walk independently and had no important cognitive deficits; therefore, extrapolations to older adults with impaired cognition and/or different functional status should be made with caution.

## 5 Conclusions

In older adults with PF&S, a higher adherence to a multimodal PA programme is associated with a greater reduction in the risk of mobility disability. Changes in physical performance and body composition parameters related to PF&S are also dependent on exercise frequency, especially in those with baseline SPPB scores of 3–7. These findings support the need to prescribe PA interventions considering baseline functional status and targeting different levels of adherence to preserve independent mobility in frail older adults.

## Ethics Statement

The study protocol was approved by the ethics committee of the Università Cattolica del Sacro Cuore, Rome, Italy (protocol No 15611/15), and was subsequently ratified by the ethics committees of all participating institutions. All participants gave informed, written consent prior to their inclusion in the study. It has been performed in accordance with the ethical standards laid down in the 1964 Declaration of Helsinki and its later amendments. The authors comply with the ethical guidelines for authorship and publishing in the *Journal of Cachexia, Sarcopenia and Muscle* [32].

## Conflicts of Interest

All authors have completed the ICMJE uniform disclosure form at www.icmje.org/coi_disclosure.pdf and declare: The present work was funded by a grant from the Innovative Medicines Initiative Joint Undertaking: R.C., L.R.‐M., M.T., M.C., A.C., A.J.C.‐J., P.V.J., M.M., Fa.L., R.R.‐W., I.R., A.M.W.J.S., C.C.S., A.J.S., A.S., T.S., A.T., E.T., B.V., S.v.H., Fr.L. and E.M. received in‐kind support from the European Federation of Pharmaceutical Industries and Associations as part of the Innovative Medicines Initiative Joint Undertaking for the submitted work; A.Á.‐B. and H.J.C.J. have no conflicts of interest to declare; R.C. received personal fees from Abbott Nutrition outside the submitted work; A.C. received grants or contracts from Pfizer and Bristol Myers Squibb outside the submitted work; A.J.C.J. received personal fees from Abbott nutrition and Nutricia for lectures and from Nestlé Health Science Honoraria for lectures and manuscript writing outside the submitted work; R.R.‐W. received public funding from the EU (paid to university), personal fees from AstraZeneca, GSK, Nestlè, Nutricia/Danone, VAMED and Fresenius, support for attending meetings and/or travel from GSK and stock from Actimed, all outside the submitted work; C.C.S. declares to be EuGMS Past President, EICA President elect and Board of Directors Felix Platter Spital (Basel, Switzerland); A.T. received support from the CHU Limoges (France) outside the submitted work; T.S. declares to be the Chairperson of the National Dyslipidemia Guideline Group; received support from the University of Helsinki (Finland), Kela, Sohlberg Foundation; and personal payments from Nutricia advisory board: Valio and support for attending meetings and/or travel from EuGMS; E.T. received support from the Charles University in Prague and personal fees from Nestlè and Pfizer for lectures outside the submitted work; B.V. declares to be a leadership or fiduciary role in the ICFSR; S.v.H. received grants or contracts from Amgen, AstraZeneca, Boehringer Ingelheim, Pharmacosmos, IMI and the German Center for Cardiovascular Research (DZHK); personal payments from Amomed, AstraZeneca, Bayer, Boehringer Ingelheim, BRAHMS, Edwards Lifesciences, MSD, Novo Nordisk, Novartis, Pfizer, Pharmacosmos, Respicardia, Roche, Servier and Vifor; and stock from Actimed, all outside the submitted work; F.L. received personal fees from Abbott outside the submitted work; E.M. received personal fees from Pfizer outside the submitted work; no other relationships or activities that could appear to have influenced the submitted work.

## Supporting information


**Figure S1** Seven‐day actimetry by intervention arm according to baseline short physical performance battery (SPPB) score category. SE = standard error.
**Figure S2** Kaplan–Meier curves for incident mobility disability in participants with baseline short physical performance battery (SPPB) score of 8 or 9. The graph is truncated at 36 months, after which two additional mobility disability events were recorded in the multicomponent intervention group and one in the lifestyle education group. CI = confidence interval.

**Table S1** Probability of incident mobility disability according to baseline SPPB score category, removing deaths.
**Table S2**. Effect of adherence to physical activity on changes (∆) in physical performance tests and appendicular lean mass according to baseline SPPB score category.
**Table S3**. Effect of time on changes (∆) in physical performance tests and appendicular lean mass according to baseline SPPB score category.

## SPRINTT Consortium Partners

*Astellas Pharma Europe BV* (Leiden, The Netherlands): Andeleeb Dahy, Makoto Kashiwa; *Bluecompanion Ltd*. (London, UK): Susanna Del Signore; *Boehringer Ingelheim International GmbH* (Ingelheim am Rhein, Germany): Laurent Nicolas, Joachim Scholpp; *Caretek S.r.l*. (Turin, Italy): Gianluca Zia (co‐leader of the ICT Enabling Infrastructure and Operations work package), Cinzia Bertuzzi, Sabina De Giorgi, Luca Feletti, Alessandro Loria, Davide Mantovani, Elisa Marchioro, Francesco Mocci, Alberto Sacco, Maria Grazia Varesio; *Centre Hospitalier Universitaire de Limoges* (Limoges, France): Achille Tchalla (local principal investigator), Maxime Billot, Noëlle Cardinaud, Muriel Castelli, Marion Charenton‐Blavignac, Cecilia Ciccolari‐Micaldi, Thierry Dantoine, Caroline Gayot, Nicolas Giroult, Anael Larreur, Cécilie Laubarie‐Mouret, Delphine Marchesseau, Thomas Mergans, Thai Binh Nguyen, Arnaud Papon, Johann Ribet, Isabelle Saulnie; *Centre Hospitalier Universitaire de Toulouse* (Toulouse, France): Bruno Vellas (local principal investigator and SPRINTT co‐principal investigator for the academia), Gabor Abellan Van Kan, Virginie Biville, Lauréane Brigitte, Matteo Cesari (academic co‐leader of the Clinical Consensus over Indication, Target Population and Clinical Trial Design for Data Generation work package), Carole Cervera, Céline Cluzan, Muriel Croizet, Sophie Dardenne, Marie Dorard, Charlotte Dupuy, Emilie Durand, Catherine Faisant, Sophie Guyonnet, Rémi Mauroux, Agathe Milhet, Sylvie Montel, Pierre‐Jean Ousset, Cécile Picauron, Gaelle Soriano, Bernard Teysseyre; *Diabetes Frail Ltd*. (Droitwich Spa, UK): Alan Sinclair (local principal investigator), Sital Harris, Allison Ogborne, Sarah Ritchie, Harriet Sinclair, Lois Tirrell, Caroline Sinclair; *EU‐Open S.r.l*. (Veronella, Italy): Alfredo Cesario (co‐leader of the Regulatory Consensus work package), Barbara Cabin, Pim de Boer, Claire Ignaszewski, Ingrid Klingmann; *Friedrich‐Alexander‐Universität Erlangen‐Nürnberg* (Nurnberg, Germany): Cornel C. Sieber (local principal investigator), Tina Auerswald, Christof Engel, Anna Franke, Ellen Freiberger, Ulrike Freiheit, Susann Gotthardt, Karin Kampe, Robert Kob, Christine Kokott, Carolin Kraska, Christian Meyer, Veronika Reith, Hanna Rempe, Daniel Schoene, Gabrielle Sieber, Kerstin Zielinski; *University of Göttingen Medical Center* (Göttingen, Germany): Stefan D. Anker (academic leader of the Biomarkers Qualification work package), Nicole Ebner, Stephan von Haehling; *GlaxoSmithKline Research and Development Ltd*. (Brentford, UK): Michael Benecky; *Hospital Universitario de Getafe* (Getafe, Spain): Leocadio Rodriguez‐Mañas (local principal investigator), Karim Patricia Aguirre‐Cocha, Shirley Soledad Alamo‐Ascencio, Alejandro Álvarez‐Bustos, Raquel Bernabé Espiga, Betty Davies, Jimmy Gonzales Turin, Tania Guevara, Olga Laosa Zafra, Andrea Julia López Diez‐Picazo, Luis Lluch‐Lafuente, Irene Molina‐Hermosilla, Laura Pedraza Sepulveda, Juan Luis Sanchez‐Sanchez, Carlos Sanchez Puelles y Myriam Valdés Aragonés; *Hospital Universitario Ramón y Cajal—IRYCIS* (Madrid, Spain): Alfonso J. Cruz‐Jentoft (local principal investigator and academic co‐leader of the Clinical Study Implementation and Operations work package), Juan Álvarez‐Santos, Belén Fernández‐Jiménez, Jesús Mateos‐del Nozal, Beatriz Montero‐Errasquín, Beatriz Ponce‐Moreno, Cristina Roldán‐Plaza, Alfonso Romera‐de Vicente, Vicente Sánchez‐Cadenas, Carmen Sánchez‐Castellano, Elisabet Sánchez‐García, María Nieves Vaquero‐Pinto; *INSERM/Université Toulouse III—Paul Sabatier UMR1027* (Toulouse, France): Sandrine Andrieu, Alessandro Blasimme, Cedric Dray, Emmanuelle Rial‐Sebbag, Philippe Valet; *Institut de Recherches Internationales Servier IRIS* (Suresnes, France): Carmen Gorostiaga Ayestarán (EFPIA co‐leader of the Clinical Study Implementation and Operations work package), Laurence Laigle, Itziar Martinez‐Melchor, Belen Surroca; *IRCCS INRCA* (Ancona, Italy): Fabrizia Lattanzio (local principal investigator), Stefania Ambrosi, Renato Baldoni, Serena Bernabei, Anna Rita Bonfigli, Silvia Bustacchini, Barbara Carrieri, Antonio Cherubini (leader of the Stakeholder Information and Results Dissemination work package), Anna Rita Costantini, Michela Cucchi, Giuseppina Dell'Aquila, Emma Espinosa, Luciano Izzo, Massimiliano Fedecostante, Michela Mannoni, Antonella Mengarelli, Marino Modestino, Emanuele Monterubbianesi, Stefano Piomboni, Antonia Scrimieri, Eddy Severini, Fabiana Mirella Trotta, Lorella Vece, Susanna Venere, Elisa Zengarini; *Landspitali National Hospital and University of Iceland* (Reykjavik, Iceland): Pálmi V. Jónsson (local principal investigator), Milan Chang, Hrafnhildur Eymundsdóttir, Ólöf Guðný Geirsdóttir, Steinn Baugur Gunnarsson, Steinunn Guðnadóttir, Alfons Ramel, Konstantín Shcherbak; *Medizinische Universität Graz* (Graz, Austria): Regina Roller‐Wirnsberger (local principal investigator), Gerhard Wirnsberger; *Novartis Pharma AG* (Basel, Switzerland): Ronenn Roubenoff (Scientific Coordinator of SPRINTT and EFPIA co‐leader of the Clinical Consensus over Indication, Target Population and Clinical Trial Design for Data Generation work package), Sheena Kao, Ram R. Miller (EFPIA co‐leader of the Biomarkers Qualification work package), Romain Barnouin; *Roessingh Research and Development BV* (Enschede, The Netherlands): Lex van Velsenm, Miriam Vollenbroek‐Hutten; *Sanofi‐Aventis Recherche & Développement* (Chilly‐Mazarin, France): Philippe Bordes (project coordinator and EFPIA co‐leader of the Project Management and Oversight work package), Christian Asbrand, Raphael Bejuit (EFPIA co‐leader of the Evaluation of Results work package), Sandrine Durand, Florence Joly (EFPIA co‐leader of the Health Technology Assessment work package), Klaus Flechsenhar, Harmonie Goyeau, Régis Le Lain (EFPIA co‐leader of the Regulatory Consensus work package), Jerome Mshihid, Aurèle Ndja (EFPIA co‐leader of the ICT Enabling Infrastructure and Operations work package); *Silesian Hospital in Opava* (Opava, Czech Republic): Ingrid Rýznarová (local principal investigator), Ivana Drastichova, Eva Hasaliková, Radim Hucko, Seget Jakub, Monika Janácová, Michaela Kilmková, Martina Parízková, Kristyna Pavelková, Michaela Redrova, Petra Rusková; *Università Cattolica del Sacro Cuore* (Rome, Italy): Roberto Bernabei (SPRINTT principal investigator for the academia), Francesco Landi (local principal investigator and academic co‐leader of the Clinical Study Implementation and Operations work package), Michele Basile, Damiano Biscotti, Claudio Boni, Vincenzo Brandi, Marianna Broccatelli, Riccardo Calvani, Carilia Celesti, Americo Cicchetti (academic co‐leader of the Health Technology Assessment work package), Hélio Jose Coelho‐Junior, Agnese Collamati, Silvia Coretti, Emanuela D'Angelo, Mariaelena D'Elia, Eugenio Di Brino, Giovanni Landi, Luca Mariotti (project manager and academic co‐leader of the Project Management and Oversight work package), Anna Maria Martone, Emanuele Marzetti (academic co‐leader of the Evaluation of Results work package), Elena Ortolani, Teodosio Pafundi, Cecilia Pantanelli, Anna Picca, Matteo Ruggeri, Filippo Rumi, Sara Salini, Giulia Savera, Elisabetta Serafini, Matteo Tosato, Davide L. Vetrano, Fabio Vitale; *Università degli Studi di Firenze* (Florence, Italy): Mauro Di Bari (academic co‐leader of the Evaluation of Results work package); *Università degli Studi di Parma* (Parma, Italy): Marcello Maggio (local principal investigator), Elisa Adorni, Fulvio Lauretani, Yari Longobucco, Giovanna Maria Pelà, Sara Tagliaferri; *Université de Paris* (Paris France): Thomas Rapp (academic co‐leader of the Health Technology Assessment work package), Yves Arrghi, Bastian Ravesteinj, Jérôme Ronchetti, Quittterie Roquebert, Jonathan Sicsic, Nicolas Sirven; *Universiteit Maastricht* (Maastricht, The Netherlands): Annemie M. W. J. Schols (local principal investigator), Harry Gosker, Jos M. G. A. Schols, Lisanne Schuurman, Nick Smeets, Coby van de Bool, Claire Weling; *University of Helsinki and Helsinki University Hospital* (Helsinki, Finland): Timo Strandberg (local principal investigator), Katja Hallikas, Marjatta Herranen, Laura Hyvönen, Kirsi Ikonen, Satu Jyväkorpi, Anne Karppi‐Sjöblom, Kaisa Karvinen, Tarja Kindstedt, Saana Leirimaa, Hanna Öhman, Kaisu Pitkälä, Anja Punkka, Anna‐Maria Saavalainen, Tuulia Salo, Katja Sohlberg, Reijo Tilvis, Annele Urtamo, Hannu Vanhanen; *Univerzita Karlova v Praze* (Prague, Czech Republic): Eva Topinková (local principal investigator), Lucie Bautzká, Tereza Gueye, Ilona Juklíčková, Pavla Mádlová, Helena Mejstříková, Helena Michálková, Eva Klára Novotná, Tereza Vágnerová; *Uniwersytet Jagiellonski Collegium Medicum* (Krakow, Poland): Anna Skalska (local principal investigator), Ewa Blaszczyk‐Bebenek, Marcin Cwynar, Joanna Czesak, Paulina Fatyga, Malgorzata Fedyk‐Lukasik, Tomasz Grodzicki, Paulina Jamrozik, Zbigniew Janusz, Ewa Klimek, Sylwia Komoniewska, Maria Kret, Maciej Ozog, Agnieszka Parnicka, Katarzyna Petitjean, Anna Pietrzyk, Karolina Piotrowicz, Barbara Skalska‐Dulinska, Damian Starzyk, Katarzyna Szczerbinska, Borys Witkiewicz, Anna Wlodarczyk, Wieslawa Zgud.
