# Supplementary material for: Adherence to Physical Activity and Incident Mobility Disability in Older Adults With Mobility Limitations
Source: J Cachexia Sarcopenia Muscle. 2025 Jun 18;16(3):e13870. doi: 10.1002/jcsm.13870 (PMC12176070; doi:10.1002/jcsm.13870)
Supplement: Supplementary file 1 — Figure S1 Seven‐day actimetry by intervention arm according to baseline short physical performance battery (SPPB) score category. SE = standard error. Figure S2 Kaplan–Meier curves for incident mobility disability in participants with baseline short physical performance battery (SPPB) score of 8 or 9. The graph is truncated at 36 months, after which two additional mobility disability events were recorded in the multicomponent intervention group and one in the lifestyle education group. CI = confidence interval. [file JCSM-16-e13870-s002.pdf]

Supplementary appendix

TABLE OF CONTENTS

SPRINTT trial protocol (only version).......... 2

Statistical analysis plan (only version).......... 80

SPRINTT trial sites.......... 126

SPRINTT consortium partners.......... 127

Figure S1.......... 131

Figure S2.......... 132

Sarcopenia and Physical fRailty IN older people:
multi-componenT Treatment strategies

SPRINTT

A randomised clinical trial comparing the efficacy of a multicomponent intervention (based on long-term structured physical activity, nutritional counselling and information & communication technology intervention) versus a Healthy Aging Lifestyle Education (HALE) programme for preventing incident mobility disability in community-dwelling older persons with physical frailty and sarcopenia

Protocol

Table of Contents

1. Synopsis 5
2. Summary 12
3. Background and Rationale 13
4. Overview of Trial Design 15
4.1. Design 15
4.2. Primary hypothesis 15
4.2.1 Primary Objective 15
4.2.2. Primary outcome 16
4.3. Secondary Hypotheses 16
4.3.1. Secondary Objectives 16
4.3.2. Secondary outcomes 16
5. Study Population 17
5.1. Eligibility criteria 17
5.1.2. Inclusion criteria 17
5.1.2.1. Demographic characteristics 17
5.1.2.2. Physical function, body composition and willingness to participate 17
5.1.3. Exclusion criteria 17
5.1.3.1. Informed consent 18
5.1.3.2. General 18
5.1.3.3. Clinical conditions 18
5.1.4. Temporary suspension of the screening procedures 19
5.2. Establishing Eligibility 19
5.3. Justification of the main eligibility criteria 19
5.3.1. Poor physical performance 20
5.3.2. Low muscle mass 20
5.3.3. Absence of mobility disability 20
6. Interventions 21
6.1. Intervention Overview 21
6.2. Multi-component Intervention 21
6.2.1. Physical Activity Intervention 21
6.2.1.1. Intensity of training 22
6.2.1.2. Mode and Frequency 22
6.2.1.3. Participant's familiarisation with the PA intervention 22
6.2.1.4. PA phases 23
6.2.1.4.1. Adoption phase (weeks 1-52) 23
6.2.1.4.2. Maintenance phase (week 53 through the end of the trial) 23
6.2.2. Nutritional assessment and intervention 23
6.2.2.1. Dietary assessment and nutritional intervention 24
6.3. Healthy Aging Lifestyle Education (HALE) programme 24
6.3.1. Mode and Frequency 24
6.3.2. General Content and Structure of Intervention Modules 25
7. Measures and procedures 25
7.1. Informed Consent 25
7.2. Measures 25
7.3. Timeline and Table of Investigations 25
7.3.1. 400 Meter Walk Test 28
7.3.2. Short Physical Performance Battery (SPPB) 28
7.3.3. Hand Grip Strength 29
7.3.4. Pepper Assessment Tool for Disability 29

7.3.5. Self-reported physical activity 29
7.3.6. Vital signs and anthropometric measures 30
7.3.7. Medication Inventory 30
6.3.8. ECG 30
7.3.9. Cognition 30
7.3.9.1. Mini Mental State Examination (MMSE) 30
7.3.9.2. Trail Making Test (Part A and B) 30
7.3.10. Health-related Quality of Life 31
7.3.11. Center for Epidemiologic Studies Depression Scale (CES-D) 31
7.3.12. Nutrition 31
7.3.13. Sociodemographic, economic and behaviour information 31
7.3.14. Comorbidity 31
7.3.15. Screening for sarcopenia 31
7.3.16. Health Care Utilisation 32
7.3.17. Data collection using technological devices 32
7.3.18. Standard blood analysis 32
7.4. Procedures 33
7.4.1. Bio-bank establishment 33
7.4.1.1. Collection of biological samples 33
7.4.1.2. Conditions of sampling 33
7.4.1.3. Coding and labelling procedures 33
7.4.1.4. Storage 33
7.4.1.5. Objective of the bio-bank 33
7.4.2. Dual energy X-ray absorptiometry 34
7.4.2.1. DXA operations manual 34
7.4.2.2. Training of DXA technicians 34
7.4.2.3. DXA standardisation 34
7.4.3. Randomisation 34
7.4.3.1. Final Eligibility Assessment 34
7.4.3.2. Randomisation algorithm 35
7.4.3.3. Masking or Blinding 35
8. Information and Communication Technologies (ICT) 35
8.1. ICT Architectural Design 35
8.2. Clinical Knowledge Hub (CKH) 37
8.3. eCRF 37
8.3.1. eCRF User support 38
8.4. Software maintenance 38
8.5. Quality Assurance 38
8.6. Data management 39
8.7. DXA data flow 40
8.8. Nutritional follow-up 41
8.9. Biomarkers 42
8.10. Adamo watch 42
8.11. Devices validation 44
8.12. Devices deployment 44
8.13. Packages quality assessment 45
9. Recruitment and Retention 45
9.1. Recruitment 45
9.2. Screening Process 46
9.3. Retention and drop-out recovery 46
9.3.1. Identifying Secondary/Proxy Contacts 46
9.3.2. Retention Promotion Efforts 46
9.3.3. Drop-out Recovery Efforts 47
9.3.4. Monitoring Recruitment and Retention 48
9.3.5. Retention and Efforts to Maintain Contact with Inactive Participants 48
9.3.6. Efforts to Maintain Contact with Inactive Participants 48

9.3.7. Monitoring and Quality Control of Recruitment and Retention 48
10. Sample size considerations and statistical analysis 49
10.1. Sample size considerations... 49
10.2. Statistical analysis 50
10.2.1. Primary efficacy endpoint... 50
10.2.2. Secondary efficacy endpoints 51
11. Safety considerations..51
11.1. Participant Safety and Confidentiality 51
11.2. Data Safety Monitoring Board 51
11.3. Medical Problems Detected During the Study Assessments... 52
11.4. Safety Considerations for Study Assessments 52
11.5. Safety Considerations for the Physical Activity Intervention 55
11.6. Pre-Physical Activity Safety Screening 55
11.6.1. Reasons for not performing physical activity stress testing. 55
11.7. Safety Measures during Physical Activity 56
11.8. Safety considerations for DXA examination... 57
11.9. Adverse Events 57
11.10. Confidentiality.. 58
12. Mitigation plans 60
13. Ethics..61
13.1.Institutional Review Board(s)/Independent Ethics Committee(s)... 61
13.2. Study conduct.. 61
13.3. Modification of the information and consent form 61
14. Study sites 63
14.1. Procedures for selection of study sites 63
14.2. Brief description of the SPRINTT study site... 64
14.2.1. Catholic University of the Sacred Heart - Centre for Geriatric Medicine 64
15. References...66
16. Appendices 71
Appendix 1: World Medical Association Declaration of Helsinki 72

1. Synopsis

Title of study: SPRINTT (Sarcopenia and Physical fRailty IN older people: multi-componenT Treatment strategies)
A randomised clinical trial comparing the efficacy of a multicomponent intervention (based on long-term structured physical activity and nutritional counselling) versus a Healthy Aging Lifestyle Education (HALE) programme for preventing incident mobility disability in community-dwelling older persons with physical frailty and sarcopenia

Protocol code: **SPRINTT**

Coordinators
International Coordinators: Pr. Cruz-Jentoft (Spain), Pr. Landi (Italy)
National Coordinators per regional area:

- Eastern Countries: Pr. Topinkova (Czech Rep) & Pr. Skalska (Poland),
- France: Pr. Vellas
- Germany & Austria: Pr. Sieber
- Italy: Pr. Landi
- Nordic Countries & The Netherlands: Pr. Strandberg (Norway) & Pr. Schols (The Netherlands)
- Spain: Pr. Rodriguez-Manas
- UK: Pr. Sinclair

Study centre*(s)*
Multicentre study = total number of centres: 14 (7 regional areas with 2 centres each)
total number of countries: 9
number of centres/country: 1 or 2

Study period	Type of study:
Study duration for the participant: 24 months of interventions (+ possible additional follow-up according to the time of inclusion in the study and interim power reassessment)<br>Study initiation date (planned date of first visit of first participant):<br>• *September 2015 (TBC)*<br>Study completion date: (planned date of last visit of last participant):<br>• *September 2018 (TBC)*	**Phase III**

Study objective(s)

Primary objective:

- To evaluate the effectiveness of a multicomponent intervention (MCI) programme (physical activity [PA], nutritional counselling/dietary intervention, and information & communication technology [ICT] intervention) compared with a healthy aging lifestyle education (HALE) programme on the hazard rate of mobility disability, in non-disabled older people with physical frailty and sarcopenia (PF&S).

Secondary objectives:

- To evaluate the effect of the MCI programme compared with the HALE programme on relevant health-related outcomes (e.g., changes in physical performance, muscle mass, cognitive function, incidence of falls, quality of life, use of healthcare services, mortality, etc.), in non-disabled physically frail and sarcopenic older people;
- To refine the definition of PF&S by identifying those variables predictive of progression towards disability in the HALE group;
- To describe non-responders participants in the MCI group by identifying variables predictive of poor response to the intervention in order to characterise the subgroup of participants who might be eligible from additional treatment(s), including drugs;
- To evaluate the role of different variables in predicting outcomes;

- To evaluate the safety and tolerability of MCI and HALE interventions;
- To qualify biomarkers for PF&S;
- To develop a health economic model for the clinical take-in-charge of PF&S.

Methodology

International, multicentre, single-blind, 2 parallel groups, randomised trial.

Number of participants

Total: 1500 included participants across 7 regional European geographic areas (2 centres per region); For each group: 750 participants

Diagnosis and criteria for inclusion
Inclusion criteria

Demographic characteristics:

- Men and women aged ≥ 70 years.

Physical function, body composition and lifestyle criteria:

a. Short Physical Performance Battery (SPPB) score between 3 (included) and 9 (included);
b. Able to complete the 400-m walk test within 15 min without sitting down, help from another person, use of a walker, or stopping for more than 1 minute at a time;
c. Presence of low muscle mass according to results from a Dual Energy X-Ray Absorptiometry (DXA) scan. In agreement with the Foundation for the National Institutes of Health Sarcopenia Project (FNIH) report [32], low muscle mass will be defined as:
    i. Body mass index-adjusted appendicular lean mass (aLM; i.e., the sum of lean mass from both arms and legs): <0.789 in men, and <0.512 in women, OR
    ii. aLM <19.75 kg in men and <15.02 kg in women
d. Willingness to be randomised to either intervention group and to follow the study protocol.

Exclusion criteria

Informed consent

a. Unable or unwilling to provide informed consent or accept randomisation to either study group

General

a. Plans to relocate out of the study area within the next 2 years or plans to be out of the study area for more than 6 consecutive weeks in the next year
b. Residence in long-term care
c. Household member enrolled in the study

Clinical conditions:

a. Current diagnosis of schizophrenia, other psychotic or bipolar disorder. Depression is not an exclusion criterion
b. Consumption of more than 14 alcoholic drinks per week
    - *One alcoholic drink (equal to 14.0 grams of pure alcohol) corresponds to:*
        - *36 cc of beer (5% alcohol content)*
        - *24 cc of malt liquor (7% alcohol content)*
        - *15 cc of wine (12% alcohol content)*
        - *4.5 cc of distilled spirit or liquor (40% alcohol content)*
c. Difficulty communicating with the study personnel due to speech, language, or (non-corrected) hearing problems
d. Mini Mental State Examination (MMSE) lower than 24/30
e. Severe osteoarthritis (e.g., awaiting joint replacement) that would interfere with the ability to participate fully in either study arm
f. Cancer requiring treatment in the past 3 years, except for non-melanoma skin cancers or cancers that have an excellent prognosis (e.g., early stage breast or prostate cancer)
g. Lung disease requiring regular use of supplemental oxygen

h. Inflammatory conditions requiring regular use of oral or parenteral corticosteroid agents
i. Severe cardiovascular disease (including New York Heart Association [NYHA] class III or IV congestive heart failure, clinically significant valvular disease, history of cardiac arrest, presence of an implantable defibrillator, or uncontrolled angina)
j. Upper and/or lower extremity amputation
k. Peripheral arterial disease Lériche-Fontaine 3 or 4
l. Parkinson's disease or other progressive neurological disorder
m. Renal disease requiring dialysis
n. Chest pain, severe shortness of breath, or occurrence of other safety concerns during the baseline 400-metre walk test
o. Current participation in a structured PA program, physical therapy or cardiopulmonary rehabilitation
p. Current enrolment in another RCT involving lifestyle, nutrition, or pharmaceutical interventions
q. Other medical, psychiatric, or behavioural factors that in the judgment of the principal investigator may interfere with the study participation or the ability to autonomously follow either the MCI or the HALE programmes
r. Other illness of such severity that life expectancy is expected to be less than 12 months
s. Clinical judgment concerning safety or non-compliance

Temporary exclusion criteria

Subjects excluded for one of the temporary medical conditions listed below may be rescreened after a period that is considered clinically appropriate by the local study physician. The participant will maintain the same informed consent form and will not change his/her participant ID. Nevertheless, all the eligibility criteria (not only those having temporarily suspended the eligibility validation) will be checked. This approach will maximise the efficacy of the recruitment strategies and (in parallel) avoid double versions of signed informed consent forms and participant's ID for the same individual.

a. Uncontrolled hypertension (systolic blood pressure >200 mm Hg, or diastolic blood pressure >110 mm Hg)
b. Uncontrolled diabetes with recent weight loss, diabetic coma, or frequent hypoglycaemia
c. Hip fracture, hip or knee replacement, or spinal surgery in the past 6 months
d. Serious cardiac conduction disorder (e.g., third-degree heart block), uncontrolled arrhythmia, new Q waves within the past 6 months or ST-segment depression (>3 mm) on the ECG
e. Myocardial infarction, major heart surgery (i.e., valve replacement or coronary bypass graft), stroke, deep vein thrombosis, or pulmonary embolism in the past 6 months
f. Use of growth hormone, oestrogens, progesterone, or testosterone supplementation in the past 3 months

Withdrawal criteria

At any time, a participant can stop her/his participation in the study.
The reasons for premature discontinuation of the study are:

a. occurrence of any event that, according to the investigator, may interfere with the study participation or create an unjustified risk for the participant;
b. illness or a condition leading to prolonged immobilisation;
c. major deviation(s) to protocol incompatible with continuation in the study;
d. any medical event requiring administration of an unauthorised concomitant treatment;
e. non-medical reason (to be carefully described);
f. lost to follow-up: when the investigator has no news of the participant, he/she must make every effort to contact him/her, to establish the reason for study discontinuation, and to suggest the participant comes to an end-of-study visit. If all these attempts fail, the investigator can then declare the participant is "lost to follow-up". The investigator should document all these attempts in the corresponding file.

Multi-Component Intervention - MCI group

The MCI programme will consist of following elements:

- a PA program (including aerobic, strength, flexibility and balance training);
- a full nutritional assessment and dietary counselling;

- an ICT intervention.

The PA component will be performed both at the clinical centre and at home according to the training schedule described in chapter 5. Home-based sessions could be replaced by an additional centre-based session in case of non-compliance at home or if preferred by the participant.
The nutritional intervention will comprised a complete nutritional assessment, followed by dietary counselling. The two components will be administered at all clinical visits.
The ICT component will involve the implementation of a devoted technological device that will be used to record data pertinent to the study outcomes (fall events and PA levels). In particular, an actimeter will be employed to record PA levels/variations. This information will be used to support the elaboration of a personalised training programme.

HALE group

The HALE programme will consist of:

- regular meetings in small groups according to the schedule described in chapter 5;
- short (5-10 min) instructor-led program of upper extremity stretching exercises at the end of the meeting;
- education on healthy diet + information of the participant's primary care physician in case of pathological nutritional parameters are found at the clinical visits.

Vitamin D supplementation

During the study, participants in both groups could be prescribed vitamin D supplementation in case of insufficiency or deficiency, in accordance with their primary care physician. A serum 25-hydroxyvitamin D (25-OH-D) concentration of 30 ng/mL (75 nmol/L) will be considered the minimum goal.

Duration of the MCI and HALE programs

24 months; an extension up to 36 months may be proposed based on study power consideration, as described in chapter 9.

Criteria for evaluation

<u>Efficacy measurements</u>:

Primary efficacy endpoint:

- Incidence of mobility disability defined as incident inability of the participant to complete a 400-m walk within 15 min without sitting, help of another person, use of a walker, or stopping for more than 1 minute at a time.

Secondary efficacy endpoints:

a. Changes in physical performance measures
    - SPPB
    - Handgrip strength
    - ADL
    - Instrumental ADL (IADL)
    - Usual gait speed over 4 metres
    - Pepper Assessment Tool for Disability (PAT-D)
b. Changes in body composition measures (assessed using DXA), anthropometric parameters (e.g., body mass index [BMI], mid-arm circumference, calf circumference), and nutritional status (Mini Nutritional Assessment-Short Form, MNA-SF)
c. Changes in cognitive function (assessed using MMSE) and mood (i.e., Center for Epidemiological Studies-Depression scale, CES-D)
d. Incidence of falls (assessed using self-reported questionnaire and a dedicated technological device)
e. Modifications of quality of life (measured using the EuroQoL-5D instrument)
f. Differences in use of healthcare services (including outpatient clinics, emergency room admissions, hospitalisations, institutionalisations)
g. Mortality rate

Safety measurements:
- Assessment of adverse events;
- Clinical examination parameters (i.e. systolic and diastolic blood pressure, pulse rate, body weight, BMI);
- Electrocardiogram (ECG);
- Assessment of biological parameters (i.e. standard blood biochemistry, haematology, urinalysis).

Statistical methods

It is planned to include in the study 1200 participants with an SPPB <8, in whom the effects of the intervention would be primarily evaluated, plus 300 participants with an SPPB ≥ 8 for exploratory purpose. A hierarchical procedure will be applied for the analyses. Primary analyses will be limited to participants with an SPPB score <8 and, if the p value for this primary comparison is below 5%, a subsequent round of analysis will be performed in the entire sample of 1500 participants.

Sample size

The sample size calculation is based on the LIFE-M study database. Specifically, survival analyses were run according to different levels of SPPB score (lower than 7, 7, 8 and 9). The outcomes considered were major mobility disability (i.e., inability to complete the 400-m walk test). The effect of PA on major mobility disability was negligible in participants with a baseline SPPB score ≥8 (hazard ratio=0.94; 95% CI: 0.72-1.23; p=0.65). Conversely, the hazard ratio was statistically significant in participants with a baseline SPPB score lower than 8 (hazard ratio=0.75; 95% CI: 0.59-0.94; p=0.012).

A sample of 1,200 older persons with SPPB score < 8 is estimated to provide 85% power (434 events) to detect a 25% reduction in the hazard of major mobility disability over 24 months of follow-up, considering a dropout rate of 25% over 2 years and a log rank test with a 5% two-sided alpha level. An interim blinded sample size reassessment after 11 months from the beginning of recruitment will allow taking immediate actions to preserve the study power in case the efficiency of enrolment or the number of observed events is not coherent with the original assumptions.

The inclusion in the SPRINTT RCT of participants with a baseline SPPB score of 8 and 9 would decrease the study power and, hence, the probability of success of the trial. On the other hand, restricting the enrolment to older people with SPPB score lower than 8 would jeopardize the aim of project to characterise the PF&S condition. Hence, it was decided to include a convenience sample of 300 older adults with SPPB score between 8 and 9 in the study and formulate a hierarchy for comparison: first, we will compare the effects of the interventions in participants presenting an SPPB score lower than 8 and, only in case of significant result (i.e., two sided p<5%), the comparison will extended to the whole study population.

Primary efficacy endpoint:

The primary comparison of intervention groups with respect to the distribution of time until the first post-randomisation occurrence of the primary outcome will be based on log-rank stratified by study site and gender. The primary comparison will be conducted only in study participants with baseline SPPB <8. If this primary analysis is statistically significant (p<0.05), the comparison will be extended to include the remaining 300 participants with baseline SPPB 8-9. An additional binary indicator variable will be introduced denoting a baseline SPPB score <8 or ≥8 and its interaction with the intervention group. Hazard ratio and confidence intervals will be computed for the whole population only if this interaction term can be ignored (i.e., if it is not statistically significant). The hazard ratio between intervention groups and the corresponding confidence interval will be computed using a Cox proportional hazard model with site and gender as co-variables for comparison in subjects with a SPPB score <8 and by adding the SPPB score ≥8 flag and corresponding interaction term as co-variables for the comparison in the whole study population.

The time to event is defined as the time from randomisation to the date of first occurrence of disability. Participants who do not meet this criterion will be censored at the time of their last primary outcome evaluation. In secondary analyses, in order to take into account additional covariates, a Cox proportional hazard model will be used if the underlying assumptions appear warranted. The

proportional hazards assumption will be verified by testing the treatment-by-time interaction in the Cox model.<br><br>**Secondary efficacy endpoints:**<br>For the assessment of secondary efficacy endpoints, specific models will be used for continuous outcomes (e.g., changes in physical performance, nutritional status, functional status, cognitive function, and quality of life). Specifically, changes over time between groups will be assessed by a repeated-measure mixed model with terms for intervention, time, baseline score, baseline score by intervention interaction, and time by intervention interaction. For outcomes such as incidence of falls and mortality rate, the analysis defined for the primary efficacy outcome will be replicated (except for the secondary analysis with competitive risk for the mortality rate). Additional analyses (e.g., subgroup composite endpoint analyses) will be defined in agreement with the Managing Board and the Data Safety Monitoring Board and specified in the statistical analysis plan that will be produced prior to the beginning of the analyses.
**Health economic assessment**<br>Estimation of the cost for running the two interventions: operating cost, start-up costs (investment in personal computers for the electronic system and maintenance of the systems, investment in exercise training equipment, ICT equipment), variable costs (ICT support, salary for instructors, travelling time costs for home visits), additional time for implementation of the intervention (feedback of a subset of clinicians, counselors, and ancillary personnel), and aggregate costs (individual component and various clinical and ancillary personnel).<br><br>- Cost-effectiveness analysis: comparison of the cost of running the intervention to several effectiveness measures defined by clinicians (e.g., ADL, IADL, MMSE, SPPB, QoL, etc.).<br>- Cost-utility analysis: comparison of the cost of running the intervention by quality-adjusted life years (QALYs) (use of the EuroQol-5D questionnaire to compute QALYs).<br><br>Socioeconomic determinants of PF in the cohort: a questionnaire including economic variables (e.g., monthly income, job status, job category, source of revenue, education level) and demographic variables (e.g., number of children, marital status, place of residency) will be designed.<br><br>Information dealing with potential informal caregivers will be collected using a modified version of the Resource Use in Dementia (RUD) questionnaire (adapted to measure the involvement of informal caregivers for physically frail people, and collect additional information dealing with these caregivers, such as age, education, marital status, job status, job category, income, and number of children). These analyses will explore how these variables act the level of PF&S observed in the study population.
**Biomarkers**<br>The objective of this substudy is to investigate and establish novel biomarkers, or a panel of biomarkers, to better characterise older persons with PF&S in terms of the verification of their clinical diagnosis, prognosis, and clinical course and response to the treatment under investigation. For the purposes of diagnosis of PF&S, an ancillary study will be conducted for which a control sample of older adults without PF&S will be enrolled. For use in the project, a biomarker is any quantitatively or qualitatively measurable parameter that is able to assist in clinical decision making.<br>Examples of biomarkers that could be assessed during the study include:<br>- D3-labelled urine creatine for muscle mass estimation;<br>- Urine proteomics of muscle protein breakdown;<br>Others biomarkers might be considered based on the scientific knowledge at the time of the study completion.<br>Some biomarkers might be assessed in a subgroup of participants only.

**Name of the sponsor:**			
Contractual signatories			
I, the undersigned, have read the foregoing protocol and the "Participant information and consent form" document attached to the protocol and agree to conduct the study in compliance with such documents, GCP and the applicable regulatory requirements.	**NAME**	**DATE**	**SIGNATURE**
INVESTIGATOR:			

CENTER NUMBER	

**XXX:**		**Dr XXX**	
**XXX:**		**Dr XXX**	

2. Summary

Physical function decreases with ageing leading to a wide spectrum of negative outcomes in the elderly, such as mobility disability, falls, social isolation, reduced quality of life, dependency and institutionalisation. The age-related loss of physical performance often results from multiple clinical and subclinical conditions [1].

In order to develop interventions against the disabling cascade in older persons, over the last decade special interest has been paid to the geriatric syndrome of frailty. Frailty has been defined as a "multidimensional syndrome characterized by decreased reserve and diminished resistance to stressors" in older persons [2]. If frailty is considered a pre-disability condition, it may then well serve as a target for preventive interventions.

Unfortunately, while the theoretical concept of frailty is largely agreed upon, its translation into clinical practice still presents some limitations due to the existence of multiple (and largely non-overlapping) operational definitions. Multiple instruments have been developed in order to capture this condition and make it objectively measurable. Fried and colleagues [3] hypothesized some core clinical presentations of frailty, which were then operationalised into an instrument (i.e., the frailty phenotype) validated within the Cardiovascular Health Study [4]. Differently, Rockwood and colleagues [5] used the Canadian Study of Health and Aging to develop and validate the so-called Frailty Index. During the last few years, several other instruments to measure frailty have been proposed, frequently building on these two models [6-11]. Interestingly, although the agreement among existing instruments assessing frailty is quite poor, each of them presents a strong predictive value for negative outcomes [12, 13]. In other words, each of them can be considered as legitimate and appropriately serving for the identification of the older persons at risk of negative outcomes, but consensus about a possible "gold standard" has not yet been achieved. Such major methodological issue limits the clinical identification (hence treatment) of high-risk older subjects with significant unmet need, and prevents the development of treatments for frailty [14].

The clinical picture of frailty (especially when assessed using instruments particularly focused on the physical domain) presents substantial overlap with that of sarcopenia (“a syndrome characterized by progressive and generalized loss of skeletal muscle mass and strength with a risk of adverse outcomes such as physical disability, poor quality of life and death” [15]). In other words, sarcopenia might be considered both as the biological substrate for the development of physical frailty as well as the pathway through which the negative health-related outcomes of frailty ensue [16].

Although physical frailty encompasses only a part of the wide spectrum of frailty, the identification of a definite pathophysiological basis (i.e., decline in skeletal muscle mass and function) opens new avenues for the development of interventions to slow or reverse the progression of this condition [16]. To identify clinical improvement, it is necessary that all of the components characterising a possible “Physical Frailty & Sarcopenia” (PF&S) condition (as the one proposed in the present project) are measurable and quantifiable. The conceptualisation of a PF&S condition resulting from a qualitative and quantitative decline of skeletal muscle will possibly promote significant advancements over traditional approaches for such age-related conditions. This may allow operationalising a potential condition of interest, identifying it in older subjects, and translating the research model into clinical healthcare practice.

Up to date, no large-scale intervention study specifically targeting frail European older persons has been conducted. In this scenario, the “Sarcopenia and Physical Frailty in Older People: Multicomponent Treatment Strategies” (SPRINTT) trial represents the first attempt to 1) identify a precise subset of frail elderly with unmet medical needs, and 2) implement a multi-component intervention (MCI) aimed at preventing incident disability and major negative health-related events.

SPRINTT bases its development plan on the findings of the successful Lifestyle Interventions and Independence for Elders (LIFE) trial [17]. LIFE (to be considered a reference experience in the field) is a phase III randomised controlled trial (RCT) that demonstrated the effectiveness of a physical exercise intervention at preventing mobility disability in sedentary community-dwelling older persons.

The SPRINTT trial is part of a larger project being conducted under the Innovative Medicine's Initiative (IMI), and will focus on PF&S in older persons. In addition to the planned clinical trial, the project aims at addressing academic, regulatory, and operational challenges associated with the study of this population with respect to the development of new medicinal products for this unmet medical need. Amongst other important development elements, the project intends to address these issues by reaching a clinical consensus, developing a regulatory work-stream, and sponsoring a RCT (which will compare the effects of a MCI (based on physical exercise, nutritional counselling, and Information & Communication Technology [ICT]) in community-dwelling older persons presenting PF&S). The trial should allow clear characterisation of this condition and support the identification of those individuals who may most benefit from a therapeutic intervention.

It is noteworthy that the SPRINTT project, including the RCT methodology, was approved by IMI after a thorough scientific and administrative evaluation process. The project was also ratified by the Committee for Medicinal Products for Human Use (CHMP) of the European Medicines Agency (EMA) with regard to:

1) *Operationalization of the PF&S condition*
2) *Identification of the target population*
3) *Primary and secondary outcome measures*
4) *Statistical analysis methodology*

The letter by EMA-CHMP supporting the approach adopted in the SPRINTT project is included in the Appendix of the present document.

3. Background and Rationale

The demographic transition Europe has experienced over the last several decades poses an unprecedented challenge for both a societal and healthcare perspective. The existing healthcare systems built around the traditional medical paradigm of patients suffering from a single acute illness are largely unprepared to face the increasing demands for health services that can specifically address the medical needs of older, multi-morbid people [18]. It follows that, on the one hand, a large and growing segment of the older European population is currently suffering from medical conditions that cannot be efficiently managed by the existing healthcare services. On the other hand, although prolongation of life remains an important public health goal, of even greater significance is that extended life includes preservation of the capacity to live independently and function well. Indeed, disabling conditions have shown to be extremely burdening for the individual as well as for the sustainability of healthcare systems [19]. In this scenario, the geriatric syndrome of frailty gains special interest and importance.

Based on a recent consensus definition, frailty would consist in "a multidimensional syndrome characterized by decreased reserve and diminished resistance to stressors" [2]. Simplified, frailty is "an expression of the lack of adaptive capacity of the organism" [20]. From this perspective, frailty may be viewed as a dynamic process of accelerated aging [21], which, in its early phase, is characterised by the absence of disability [22]. According to the Survey of Health, Aging and Retirement in Europe (SHARE) study, the prevalence of pre-frailty and frailty among 18,227 randomly selected community-dwellers aged >65 years

was 42.3% (40.5%-44.1%) and 17.0% (15.3%-18.7%), respectively [23]. In the absence of targeted interventions, the progression of frailty is marked by increased morbidity, disability, frequent and often inappropriate healthcare use, nursing home admission, and poor quality of life [24]. Detecting and contrasting frailty are therefore of outstanding importance for impeding the progression of the syndrome and preventing its detrimental consequences [14]. Indeed, once disability has emerged, the restoration of an adequate level of functioning is unlikely, especially when the age of the subject, the degree of disability or its duration increase [25]. Unfortunately, to date, no healthcare programs or pharmacological treatments are available for frail older people. This is largely due to the current lack of a precise, universal definition of frailty, which in turn is linked to the multidimensional nature of the condition [26]. It is therefore not by accident that the syndrome is not yet nosographically recognised (e.g., it is not present in the International Classification of Diseases 10; ICD-10) [27]. Moreover, the existing gaps in knowledge are reflected by the absence of effective interventions (either pharmacological or behavioural) against frailty. Such a barrier may be overcome by developing and validating a robust conceptual framework of frailty to achieve a practical operationalisation of the syndrome [14]. This conceptualisation should also improve the definition of the pathophysiological and clinical foundations of frailty to assist in the design and implementation of specific interventions aimed at restoring robustness or delaying the onset of adverse events (in particular, disability).

Multiple instruments have been developed to capture frailty and render the condition objectively measurable. Fried and colleagues [3] hypothesised some core clinical presentations (the frailty phenotype), which were then operationalised into an instrument evaluating exhaustion, involuntary weight loss, slow gait speed, weak muscle strength, and low energy expenditure. This instrument was subsequently validated in the Cardiovascular Health Study [4]. Rockwood and colleagues [5] used the Canadian Study of Health and Aging to develop and validate the Frailty Index, measuring the age-dependent accumulation of deficits: symptoms, signs, conditions, diseases, and disabilities. Several other instruments to measure frailty have been proposed, frequently building on these two models [6-11].

The frailty phenotype proposed by Fried and colleagues [4] is surely the most widely used and presents a better-characterised pathophysiological background [28]. The physical frailty condition depicted by the frailty phenotype has shown to be predictive of major negative health-related outcomes, including mobility disability, disability in activities of daily living (ADLs), institutionalisation, and mortality. However, the disparate elements that may underlie the frailty phenotype have led to concern that it is too heterogeneous for direct application to clinical trials, especially those with a pharmacological agent, or in the clinical setting. At the same time, it cannot be ignored that PF presents substantial overlaps with sarcopenia. In fact, many of the adverse outcomes of frailty are probably mediated by sarcopenia [29]. Therefore, sarcopenia may be considered both as the biological substrate for the development of PF and the pathway through which the negative health outcomes of frailty ensue. Although PF encompasses only a part of the frailty spectrum, the identification of a definite biological basis (i.e., skeletal muscle decline) opens new venues for the development of interventions to slow or reverse the progression of this condition. In this regard, it is noteworthy that all of the components characterising a possible PF and sarcopenia (PF&S) are measurable and quantifiable. Such a conceptualisation of PF will possibly promote significant advancements over the traditional approaches to this syndrome by enabling the precise operationalisation of the condition, a clear identification of the affected population and the rapid translation of findings to the clinical arena.

The recognition of sarcopenia as a major component of PF implies that

interventions specifically targeting the skeletal muscle may provide therapeutic and preventive advantages against frailty and its clinical correlates. However, although observational studies and some RCTs have suggested a positive effect of regular physical activity (PA) and nutritional interventions on improving physical function and/or reducing symptoms of disability in healthy older individuals and those at risk for mobility disability, definite evidence from high-quality, large-scale clinical trials is still lacking. In this regard, it is important to recognise that short-term gains in intermediate outcomes of PA programmes, such as strength and aerobic capacity, are insufficient to prove that such programmes can truly prevent frailty and disability.

The largest and longest study in this field is the LIFE study [17, 30], a multicentre RCT conducted in the United States comparing a PA programme with a successful aging educational programme in more than 1,600 sedentary older persons at risk for disability. Results showed that a structured, moderate-intensity PA programme reduced the incidence of major mobility disability (expressed as incapacity to walk 400 metres) over 2.6 years. These findings strongly suggest mobility benefit from such a programme in vulnerable older adults. However, one of the major limitations of LIFE is the lack of a detailed assessment and intervention over the nutritional domain of frailty. In fact, addressing the possible unhealthy diet of the older person may potentially help at maximising the already important benefits of PA [31].

The SPRINTT long-term clinical trial is part of a larger a project being conducted under the auspices of IMI, and will focus on PF&S in older persons. The SPRINTT project is geared to produce significant advancements in the management of frail elders by promoting a consensus among academia, regulators, industry, and patients' representatives. The SPRINTT project will produce novel and meaningful data on a large sample of "real-practice" older adults to define reference values specific for the European population to be potentially used in the future for regulatory and research purposes.

4. Overview of Trial Design

4.1. Design

The SPRINTT trial is a multicentre single-blind RCT involving a MCI (based on PA and nutrition counselling/dietary nutrition) versus a healthy aging lifestyle education (HALE) programme in 1,500 non-disabled, community-dwelling persons age 70 years and older with PF&S across nine European countries (14 recruitment sites across Europe).

The primary outcome (i.e., incident mobility disability) and the PA protocol adopted in SPRINTT are based on the previous experience of the LIFE study [17, 30]. Looking at this prior data, it is expected a limited number of primary outcome events occurring during the first months of follow-up. In order to maximise the statistical power of the analyses without modifying the maximum duration of the trial (3 years), each participant will have a different length of follow-up according to the date of his/her recruitment. In other words, the end of the follow-up will occur at the same time for all the participants. Those randomised at the beginning of the recruitment phase (12-month long) will have a longer follow-up duration (up to 3 years) compared with those included at the end of recruitment (minimum 2 years of follow-up). Hence, an average follow-up of 2.7 years (24-36 months) is foreseen.

4.2. Primary hypothesis

Compared with a HALE programme, a long-term structured PA programme sustained by a regular nutritional counselling reduces the risk of major mobility disability, defined as incapacity to walk 400 metres within 15 minutes, without sitting, help from another person or use of a walker.

4.2.1 Primary Objective

The main objective of this study is to evaluate the effectiveness of a MCI programme (PA plus nutritional counselling/dietary intervention) compared with a HALE programme on the hazard rate of mobility disability, in non-disabled PF&S older people.

4.2.2. Primary outcome

In agreement with what already tested in the LIFE study [17, 30], the SPRINTT consortium has selected the 400-metre walk test as the primary outcome measure for the full-scale trial time to the onset of major mobility disability. The objective component of the major mobility disability outcome is defined as the inability to complete a 400-metre walk test within 15 minutes, without sitting, help from another person or use of a walker. Individuals who require more than 15 minutes to complete the walk have an extremely slow pace (<0.45 m/sec), which would make their walking capacity of little utility in daily life. Selecting a higher cut-point, such as 30 or 60 minutes, makes the objective assessment impractical and does not add to the clinical significance of the outcome.

4.3. Secondary Hypotheses

Compared with random assignment to a HALE programme, random assignment to a long-term structured MCI programme reduces the risk of adverse health-related outcomes in non-disabled PF&S older people.

4.3.1. Secondary Objectives

Secondary objectives are to evaluate the effect of the MCI programme compared with the HALE programme on relevant adverse health-related outcomes, in particular:

a. Changes in physical performance measures
b. Changes in disability status
c. Changes in body composition, anthropometric and nutritional measures
d. Changes in cognitive function and mood
e. Incidence of falls and injurious falls
f. Modifications of quality of life
g. Differences in use of healthcare services
h. Differences in mortality

4.3.2. Secondary outcomes

a. Changes in physical performance measures
    - Short physical performance battery (SPPB)
    - Handgrip strength
    - Usual gait speed
b. Changes in disability status
    - Pepper Assessment Tool for Disability (PAT-D)
    - Incidence of persistent mobility disability (operationalised as the failure of completing the 400-metre walk test in two consecutive 6-month visits)
c. Changes in body composition measures (assessed using Dual Energy X-Ray Absorptiometry, DXA), anthropometric parameters (e.g., body mass index, mid-arm circumference, calf circumference), and nutritional status (Mini Nutritional Assessment-Short Form, MNA-SF)
d. Changes in cognitive function (assessed using Mini Mental State Examination, MMSE) and mood (i.e., Center for Epidemiological Studies-Depression scale, CES-D)

e. Incidence of falls (assessed using self-reported questionnaire as well as via a technological device) and injurious falls
f. Modifications of quality of life (measured using the EuroQoL-5D instrument)
g. Differences in use of healthcare services (e.g., outpatient clinics, emergency room admissions, hospitalisations, institutionalisations)
h. Mortality rate

5. Study Population

SPRINTT plans to recruit physically frail and sarcopenic, community-dwelling older persons aged 70 years and older. The specific inclusion and exclusion criteria are summarised below. These criteria are intended to select a population that is at higher risk of experiencing the major mobility disability outcome, would most likely benefit from the MCI, and would most likely comply with the intervention and assessment protocols. This age group is selected because it is at high risk of major mobility disability, and expected to have a sufficiently long life expectancy to participate in a trial that lasts up to 3 years.

5.1. Eligibility criteria

The eligibility criteria in this study are aimed at identifying persons who are physically frail and sarcopenic, that is have the clinical and biological hallmarks of functional limitation (as assessed by a battery of physical performance tests and DXA). At the same time, candidates for the SPRINTT RCT will also be non-disabled, as documented by their ability to walk 400 metres without sitting or the help of another person. Targeting this subset of the population makes it possible to recruit a non-disabled but at-risk population for a clinical trial of disability prevention. Interestingly, the eligibility criteria to be adopted in SPRINTT are very similar to those already implemented in the recently concluded LIFE study. This will not only allow the positioning of SPRINTT on the solid bases of LIFE, but also possible future comparisons between the two populations and adopted interventions.

5.1.2. Inclusion criteria

5.1.2.1. Demographic characteristics

Men and women aged ≥70 years. This age group is selected because it is at high risk of major mobility disability, and it may have a sufficiently long life expectancy to participate in a full-scale RCT, which would have duration up to 3 years.

5.1.2.2. Physical function, body composition and willingness to participate

e. SPPB score between 3 (included) and 9 (included);
f. Able to complete the 400-mtre walk test within 15 minutes without sitting down, the help with another person or the use of a walker;
g. Presence of low muscle mass according to results from a DXA scan. In agreement with the Foundation for the National Institutes of Health Sarcopenia Project (FNIH) reports [32], low muscle mass will be defined as:
    iii. Body mass index-adjusted appendicular lean mass (aLM; i.e., the sum of lean mass from both arms and legs): <0.789 in men, and <0.512 in women, OR
    iv. aLM <19.75 kg in men and <15.02 kg in women
h. Willingness to be randomised to either intervention group and to follow the study protocol.

5.1.3. Exclusion criteria

The exclusion criteria proposed in SPRINTT are mainly aimed at 1) excluding persons with specific clinical conditions that may render the intervention unsafe (i.e., severe diseases, unstable health status), and 2) avoiding the inclusion of individuals whose adherence to the protocol might be low due to clinical (e.g., cognitive impairment, dialysis) and non-clinical (e.g., plans to relocate) reasons.

5.1.3.1. Informed consent

a. Unable or unwilling to provide informed consent or accept randomisation in either study group

5.1.3.2. General

a. Plans to relocate out of the study area within the next 2 years or plans to be out of the study area for more than 6 consecutive weeks in the next year
b. Nursing-home residence
c. Household member enrolled in the study

5.1.3.3. Clinical conditions

t. Current diagnosis of schizophrenia, other psychotic or bipolar disorder.
u. Consumption of more than 14 alcoholic drinks per week
    - *One alcoholic drink (equal to 14.0 grams of pure alcohol) corresponds to:*
        - *36 cc of beer (5% alcohol content)*
        - *24 cc of malt liquor (7% alcohol content)*
        - *15 cc of wine (12% alcohol content)*
        - *4.5 cc of distilled spirit or liquor (40% alcohol content)*
v. Difficulty communicating with the study personnel due to speech, language, or (non-corrected) hearing problems
w. MMSE lower than 24/30
x. Severe arthritis (e.g., awaiting joint replacement) that would interfere with the ability to participate fully in either study arm
y. Cancer requiring treatment in the past 3 years, except for non-melanoma skin cancers or cancers that have an excellent prognosis (e.g., early stage breast or prostate cancer)
z. Lung disease requiring regular use of supplemental oxygen
aa. Inflammatory conditions requiring regular use of oral or parenteral corticosteroid agents
bb. Severe cardiovascular disease (including New York Heart Association [NYHA] class III or IV congestive heart failure, clinically significant valvular disease, history of cardiac arrest, presence of an implantable defibrillator, or uncontrolled angina)
cc. Upper and/or lower extremity amputation
dd. Peripheral arterial disease Lériche-Fontaine 3 or 4
ee. Parkinson’s disease or other progressive neurological disorder
ff. Renal disease requiring dialysis
gg. Chest pain, severe shortness of breath, or occurrence of other safety concerns during the baseline 400-metre walk test
hh. Current participation in a structured PA program, physical therapy or cardiopulmonary rehabilitation
ii. Current enrolment in another RCT involving lifestyle, nutrition, or pharmaceutical interventions
jj. Other medical, psychiatric, or behavioural factors that in the judgment of the principal investigator may interfere with the study participation or the ability to autonomously follow either the MCI or the HALE programmes

kk. Other illness of such severity that life expectancy is expected to be less than 12 months
ll. Clinical judgment concerning safety or non-compliance

5.1.4. Temporary suspension of the screening procedures

In many cases, participants may have conditions that would preclude participation in the study that could resolve. Therefore, we also define a set of criteria temporary suspending the procedures for validation of the subject's eligibility. Participants presenting such conditions may be re-contacted later during the recruitment period for completing the evaluation on a second time (in order to maximise the recruitment activities and avoiding losing the inclusion of potential participants due to temporary conditions).

This approach has already been adopted in previous RCTs, such as the LIFE [17, 30] and the DO-HEALTH (http://do-health.eu) studies.

Subjects excluded for one of the temporary medical conditions listed below can be rescreened after a period that is considered clinically relevant by the local study physician. The participant will maintain the same informed consent form and will not change his/her participant ID. Nevertheless, all the eligibility criteria (not only those having temporarily suspended the eligibility validation) will be checked. This approach will maximize the efficacy of the recruitment strategies and (in parallel) avoiding double versions of signed informed consent forms and participant's ID for the same individual.

h. Uncontrolled hypertension (systolic blood pressure >200 mmHg, or diastolic blood pressure >110 mmHg)
i. Uncontrolled diabetes with recent weight loss, diabetic coma, or frequent hypoglycaemia
j. Hip fracture, hip or knee replacement, or spinal surgery in the past 6 months
k. Serious cardiac conduction disorder (e.g., third-degree heart block), uncontrolled arrhythmia, new Q waves within the past 6 months or ST-segment depression (>3 mm) on the ECG
l. Myocardial infarction, major heart surgery (i.e., valve replacement or coronary bypass graft), stroke, deep vein thrombosis, or pulmonary embolism in the past 6 months
m. Use of growth hormone, oestrogens, progesterone, or testosterone supplementation in the past 3 months

5.2. Establishing Eligibility

Eligibility is established in a multi-step process maximising cost-effectiveness of the screening procedures. The first step could be an at-distance (telephone and/or mail) screen to assess specific inclusion and exclusion criteria. This is followed by an on-site assessment, including the administration of the SPPB, the 400-metre walk test, the MMSE, and an interview. Finally, the potential participant receives an examination by the study physician, physician assistant or nurse practitioner, who determines if conditions are present that meet exclusion criteria. The last step in the process verifying the eligibility of the subject is the body composition evaluation by DXA scan.

5.3. Justification of the main eligibility criteria

The main feature of the SPRINTT target population is represented by the PF&S syndrome, that is defined by poor physical performance (here defined as an impairment at the SPPB [33]) and low muscle mass (here defined according to the criteria proposed by the FNIH initiative [32]) in the absence of mobility disability (as incapacity to complete a 400-metre walk test).

5.3.1. Poor physical performance

In SPRINTT, the physical frailty domain will be characterised by a score ranging between 3 (included) and 9 (included) for the SPPB. Although the SPPB was not originally designed to measure the frailty status (since this condition was not conceptualised at that time), it still adequately responds to the need of detecting older persons with an increased vulnerability to stressors and exposed to higher risk of negative health-related events (including disability and death). In the absence of a "gold standard" assessment tool for frailty, it was recently proposed to restrict the focus to the very inner core of the frailty syndrome and to one of the primary outcomes of geriatric medicine: physical disability [34]. There is increasing consensus around the adoption of physical performance measures (such as the SPPB for instance [33, 35]) in addition to muscle mass to objectively assess the vulnerability of older people to endogenous and exogenous stressors (i.e., frailty) [36].

By convention, an older person with an SPPB score >9 is considered robust [33]. Alternatively, a SPPB score ≤9 identifies frail individuals and the lower the score, the worse the physical performance of the individual. An SPPB score ≤7 is commonly used to define a subgroup of particularly frail individuals [17]. In SPRINTT (consistently with the LIFE trial [17, 30]), we are proposing the use of an SPPB score ranging between 3 (included) and 9 (included). This will allow excluding from the trial too robust or too dependent subjects. At the same time, a relevant number of elders within this range of physical performance still maintain their mobility capacity (variable of primary interest for the SPRINTT trial).

In the SPRINTT trial, special attention will be given to the frailest participants (those presenting an SPPB score ≤7) because they are more likely to express the largest effect size from the intervention [17]. For this reason (and consistently with what already did in the LIFE study [30]), participants with an SPPB ≤7 will be oversampled (i.e., 80% of the recruited population).

5.3.2. Low muscle mass

In SPRINTT, the definition of the muscle mass component of the sarcopenia domain of the PF&S syndrome will rely on the operational criteria proposed by the FNIH initiative (see inclusion/exclusion criteria) [32]*.* The FNIH initiative recommended two alternative gender-specific measures used to define low muscle mass [32]. The first FNIH definition (i.e., ALM_{BMI}) is the one recommended by the FNIH project, while the second (i.e., crude ALM) is proposed as an alternative.

The ALM_{BMI} definition may enrich our target population in sarcopenic obese individuals, potentially increasing the statistical power of future analyses (due to the higher risk of events expected in this subpopulation). On the other hand, sarcopenia in subjects with normal-to-low BMI might more easily remain undetected with this criterion.

As a consequence, it was decided to follow the FNIH recommendations. Thus, each potential participant will be considered "eligible" only if presenting an ALM_{BMI} below the gender-specific cut-points indicated in the FNIH reports. When this first recommended criterion is not fulfilled, the individual will be tested with the alternative criterion (based on the crude ALM) to verify the absence of a sarcopenic phenotype. The combination of the two criteria following the FNIH recommendations will lead to the recruitment of participants with a sufficiently wide spectrum of body composition profiles.

5.3.3. Absence of mobility disability

In SPRINTT, the definition of mobility disability (primary outcome of the study) will be based on results of the 400-metre walk test. Since the outcome of the SPRINTT trial will be the prevention of mobility disability (i.e., incapacity to complete a 400-metre walk test within 15 minutes [37]), being able to pass this test represents the primary inclusion

criterion in the operationalisation of the target condition. The choice of the 400-metre walk test as a measure of the primary outcome in SPRINTT is justified by several reasons. This test provides a dichotomous result (i.e., capacity/incapacity to complete the task), which accurately reflects a specific and clinical relevant condition, which is mobility disability [37]. This definition of mobility disability has already been used in major clinical trials, in particular the LIFE pilot and full-scale studies [17, 38]. The clinical rationale behind the choice of the 400-metre walk test is that mobility disability represents the first clinically relevant step in the disabling cascade and is predictive of major health-related events.

6. Interventions

6.1. Intervention Overview

The primary aim of the SPRINTT trial is to evaluate the effect of a MCI programme [through the combination of PA and personalised nutritional counselling] compared with a HALE programme, on the hazard rate of the first occurrence of mobility disability in non-disabled older people with PF&S.

Participants will be randomised to either the MCI or the HALE programme. The investigation schedule (including all examinations, assessments to be performed, questionnaires to be answered, etc.) will be the same for all participants regardless of the randomisation group. The only difference between the two groups will be the intervention programme.

Intervention goals will be individualised based on each participant’s level of physical fitness, dietary habits or preferences, and can be modified in response to illnesses, injury, or physical symptoms. Based on previous experience, these interventions can be successfully delivered to older individuals, including frail persons, and can result in sustained participation rates, improved physical function and overall wellness.

6.2. Multi-component Intervention

6.2.1. Physical Activity Intervention

The PA intervention is based on the exercise protocol of the LIFE study [17], which has been shown to be safe and effective for the prevention of mobility disability [17] and PF [39].

The PA intervention will be of moderate intensity and consists of aerobic, strength, flexibility, and balance training [17, 30, 39]. Walking will be the primary mode of PA for preventing/postponing the outcome of major mobility disability, given its widespread popularity and ease of administration across a broad segment of the older adult population [40]. The target duration of walking will be 150 min per week. This goal will be gradually approached on the basis of the Borg’s scale [41], taking into account perceived exertion (see paragraph 5.2.1.1 "Intensity of training"). Other forms of endurance activity (e.g., stationary cycling) may be utilised on a limited basis when regular walking is contraindicated either medically and behaviourally. Each session will be preceded by a brief warm-up and followed by a brief cool-down period. In light of current clinical guidelines, participants are instructed to complete flexibility PA following each bout of walking. Moreover, two times per week, following a bout of walking, participants will be instructed during the initial phase of the programme to complete a 10-min routine focused on strengthening exercises for lower extremity muscle groups by using variable weight ankle weights. This will be followed by a brief lower extremity stretching routine. Supplementary instructional materials will be supplied to participants to reinforce the strength training occurring during centre-based instruction, so that it can be generalised to the home environment. Balance training [42] will be introduced during the adoption phase of the programme as a complement to the aerobic and strength components. The intervention will also involve encouraging participants to increase all forms of PA

throughout the day (e.g., leisure sports, gardening, use of stairs as opposed to escalators, and leisurely walks with friends).

6.2.1.1. Intensity of training

The participants will be introduced to the activities of the PA intervention in a structured way, such that they begin with lighter intensity and gradually increase the intensity over the first 2-3 weeks of the intervention. SPRINTT will promote walking for exercise at a moderate intensity and will rely on rating of perceived exertion (i.e., Borg's scale) as a method to regulate the PA intensity [41]. Accordingly, participants will be asked to walk at an intensity of 13 on the Borg scale (range 6 to 20), corresponding to activity perception “Somewhat hard”. They will be discouraged from exercising at levels that approach or exceed 15 (“Hard) or drop to a rating of 11 (“Fairly light) or below. Lower extremity strengthening exercises will be performed at an intensity of 15 to 16 for the strength training component of the program.

6.2.1.2. Mode and Frequency

The PA component is designed to be performed both at the centre and at home. During the intervention, participants will train at the centre twice a week under direct supervision of instructors. The supervised setting will allow instructors to better tailor the programme to individual needs and abilities, so as to prevent early dropout and facilitate the building of self-efficacy and support, which have been found to be key to long-term PA maintenance.

As shown in the PA schedule reported below, centre-based sessions will be supplemented, in a progressive fashion, with home-based exercises as a means of facilitating PA in multiple settings, adopting a healthier behaviour, and promoting long-term adherence. The home-based sessions could be replaced by an additional centre-based session if the participant is not compliant at home or prefers returning to the centre.

The total amount of PA will be monitored on a continuous basis by the AdamoWatch (see Chapter 7 “Information and Communication Technology”). At specified timeframes (at baseline and every 6 months) and on-demand by the investigator, the study staff will monitor the adherence to PA using such information and provide personalised feedback/tips to the participant. As back-up plan and in support of the AdamoWatch, investigators will also have results of actimetry from the ActivPAL^{TM} device (measured at baseline and every 6 months [±2 weeks]).

**Intervention staff contacts for the PA group**		
Week	***Centre-based PA***	***Home-based PA***
Adoption<br>(weeks 1-52)	2 times each week	1 time/week (weeks 1-4)<br>2 times/week (weeks 4-8)<br>Up to 3-4 times/week (weeks 8-52)
Maintenance<br>(weeks 53 – end of the trial)	2 times each week	Up to 3-4 times/week

6.2.1.3. Participant’s familiarisation with the PA intervention

As undertaken in other programmes with older adults, including the LIFE study [17, 43-45], each participant randomised to the PA group will receive a 45-min individualised introductory session. During this session, the programme is described, questions are answered, and results from each individual’s baseline assessment is utilised to tailor the programme with respect to PA progression, as well as optimise safety and participation. By recording the amount of PA of each participant, the intervention will be progressively

adapted over the course of the study. This will also allow the tracking and promotion of PA both at the centre and off-site.

6.2.1.4. PA phases

The PA intervention will comprise an adoption and a maintenance phase.

6.2.1.4.1. Adoption phase (weeks 1-52)

Two centre-based exercise instruction sessions per week will be conducted in a supervised setting. These sessions will be used to introduce the participants to the PA programme in a safe and effective manner. As previously stated, the supervised setting will allow a better tailoring of the PA programme to the participant's needs and capabilities. This approach will also represent part of the retention strategies designed to prevent early dropout and facilitate the building of self-efficacy and support. These exercise sessions will involve 40-60 minutes of exercise instruction. For those participants who miss two consecutive PA class sessions, without informing the exercise staff of their absence, exercise staff will call the participant to problem-solve ways to get him/her back to class.

In addition, the centre-based sessions will be supplemented, in a progressive fashion, with home-based exercises to promote long-term adherence and enhance the overall PA level. This has been found to be a key feature of sustained PA participation among older as well as younger adults[17, 40].

6.2.1.4.2. Maintenance phase (week 53 through the end of the trial)

The maintenance phase will consist of continued twice-per-week centre-based group exercise sessions offered to each participant, and progression of home-based PA to 3-4 times per week. For those participants who miss two consecutive PA class sessions, without informing the exercise staff of their absence, exercise staff will call the participant to problem-solve ways to get him/her back to class.

6.2.2. Nutritional assessment and intervention

Nutritional status is of course a major determinant of the person's wellbeing. Evidence suggests that nutrition represents an important and modifiable factor potentially affecting the frailty status of the older person[20, 31]. Nutrition is not only involved in the direct assessment of frailty [46], but may also play a role in the definition of the interventions aimed at restoring robustness and contrasting sarcopenia. Given its capacity to provide beneficial effects on multiple systems and at biological, clinical, and social levels, nutrition may be considered as a MCI *per se* [47]. Notably, the combination of nutritional interventions and physical exercise appears to be the most effective strategy presently available for the management of sarcopenia [31].

For a nutritional intervention to be effective against frailty and sarcopenia, it should: a) provide an adequate caloric intake; b) ensure the provision of appropriate nutrients, taking into account age, sex, health status, PA level, and comorbidities; c) provide the adequate quality and quantity of nutrients at the right time, that is, when physiologically needed [31].

In SPRINTT, the multifactorial nature of nutrition to support the beneficial effect of PA on PF&S will be exploited through the combination of individual nutritional assessment and personalised dietary recommendations. SPRINTT mostly aims at achieving two predefined nutritional targets:

- a daily total energy intake of 25 to 30 kcal/kg body weight [48];
- an average protein daily intake at least in the range of 1.0 to 1.2 g per kilogram of body weight [49].

These nutritional goals are based on expert recommendations on the topic [48, 49]. As

recommended by expert panels, nutritional targets will be adjusted according to the participant's current nutritional status and eventual comorbidities that may deserve specific dietary strategies (e.g., severe kidney dysfunction, obesity, diabetes). Nutritional targets may be achieved through dietary advice, including supplements if deemed necessary [48, 49]. Moreover, vitamin D supplementations will be recommended to participants in both groups in whom serum levels of 25OH-vitamin D are deficient or insufficient, in accordance with their primary care physician.

As recommended by the American Geriatrics Society Consensus Statement on Vitamin D for Prevention of Falls and Their Consequences [50], a serum 25 hydroxyvitamin D (25-OH-D) concentration of 30 ng/mL (75 nmol/L) should be a minimum goal to achieve in older adults, particularly in frail subjects who are at higher risk of falls, injuries, and fractures.

6.2.2.1. Dietary assessment and nutritional intervention

In each study centre, the local dietician/nutritionist (D/N) will train each participant randomised to the MCI group on how to complete a 3-day dietary record. The 3-day dietary record will be collected from each participant in the intervention group at baseline and every 12 months. In the case of incomplete data collection or implausible data reported on the diary, the D/N will perform plausibility-check for assuring the completeness of the provided information. The macro- and micronutrient composition of the diet will be determined locally by the D/N through the use of nutritional software or national dietary databases, consistently with standard assessments conducted in clinical practice. This assessment will then support the elaboration of personalised nutritional recommendations by the local D/N, in agreement with national and international guidelines (as currently done in the standard clinical practice).

Nutritional reports (e.g., translation of the 3-day dietary record in English) will be centralised in the SPRINTT Nutrition Frontend application web-system. For more details about the related data privacy, transfer and centralisation, see section “Information and Communication Technologies”. The RCT coordinating centre (i.e., Catholic University of Sacred Heart, Rome, Italy) will perform random quality checks and will be available as an additional counselling resource for complex cases. The coordinating centre will setup periodic teleconferences with local D/Ns to discuss dietary benchmarks and special cases, share nutritional solutions implemented by individual centres, and suggest nutritional strategies based on successful experiences of individual centres.

The local D/N will regularly monitor the adherence to dietary prescription, eventually proposing additional *in itinere* assessments according to clinical needs.

6.3. Healthy Aging Lifestyle Education (HALE) programme

In the LIFE trial [17], it was considered unethical the allocation of at-risk individuals to a group where no intervention was proposed (i.e., standard care). For this reason, the LIFE study control group followed a programme of education to healthy lifestyle. The same approach will be followed in SPRINTT.

6.3.1. Mode and Frequency

The HALE programme arm will meet in small groups (approximately 10-20 participants per group), two times per month with required participation at least once per month. Telephone calls will be made after missed contact to problem-solve barriers to attendance and encourage regular participation. As undertaken in the LIFE study, those participants randomised to the HALE programme will receive an individual 45-minute introductory session, by a health educator, during which time the programme is described and questions are answered.

6.3.2. General Content and Structure of Intervention Modules

The HALE programme will be based on a workshop series. Participants will receive information on a variety of topics of relevance to older adults (e.g., recommended preventive services and screenings at different ages, where to go for reliable health information, etc.). The programme also includes a short instructor-led programme (5-10 minutes) of upper extremity stretching exercises or some relaxation techniques that will be performed at the end of each class. The rationale for this “placebo exercise” activity is that it helps foster adherence to this arm of the study and increases the perceived benefit of the HALE workshop series to the participants without directly affecting the study outcomes.

7. Measures and procedures

7.1. Informed Consent

Informed consent must be obtained before participants are screened. Verbal consent will be acquired prior to the administration of the telephone screen.

7.2. Measures

In this section, the description and timeline of the measures of interest for the SPRINTT trial are provided. All data will be collected via an electronic Case Report Form (eCRF) and automatically transferred to a central system (see section 7 "Information and Communication Technology").

7.3. Timeline and Table of Investigations

The administration of the following questionnaires and measures will follow the timeline presented below. If a participant needs or decides to withdraw from the study, an end-of-study visit will be proposed with the aim of collecting key information about the subject's health status (prioritising the conduction of the 400-metre walk test for the assessment of the primary outcome).

	**PS**	**S1**	**S2**	**V1**	**V2**	**V3**	**V4**	**V5**	**V6**	**V7**	**V8**	**V9**
Type of contact	*Phone*	*Visit*	*Visit*	*Visit*	*Visit*	*Visit*	*Visit*	*Visit*	*Visit*	*Visit*	*Visit*	*Visit*
Time (D=days, M=Months)	*-D60-T0*	*-D30-T0*	*-D30-T0*	*T0*	*M3*	*M6*	*M12*	*M18*	*M24*	*M30*	*M36*	*EoS*
Verbal consent	X											
First-contact interview	X											
Informed consent		X										
Medical history		X										
Physical exam		X										
Medication inventory		X			X	X	X	X	X	X	X	X**
SPPB		X			X	X	X	X	X	X	X	X**
MMSE		X					X		X		X	X**
ECG		X				X	X	X	X	X	X	X**
Blood pressure		X			X	X	X	X	X	X	X	X**
400-m walk test		X	X*		X	X	X	X	X	X	X	X**
SARC-F		X										
Anthropometry		X		X	X	X	X	X	X	X	X	X**
DXA		X	X*				X		X		X	X**
Study eligibility checklist				X								
Planning of follow-up		X		X	X	X	X	X	X	X	X	X**
Update contact information		X		X	X	X	X	X	X	X	X	X**
Randomisation				X								
Provision of ICT kit to the participant				X								
Blood drawn				X	X		X		X		X	X**
Urine sampling				X	X		X		X		X	X**
Sociodemographic characteristics				X								
Comorbidity				X			X		X		X	X**
CIRS				X			X		X		X	X**
Motivation questionnaire				X			X		X		X	X**
EuroQoL-5D				X	X	X	X	X	X	X	X	X**
Handgrip strength				X		X	X	X	X	X	X	X**
PAT-D				X	X	X	X	X	X	X	X	X**
TMT-A and TMT-B				X			X		X		X	X**
CES-D				X		X	X	X	X	X	X	X**
MNA-SF				X		X	X	X	X	X	X	X**
Self-reported PA questionnaire				X	X	X	X	X	X	X	X	X**
HE questionnaire				X	X	X	X	X	X	X	X	X**
Accelerometry (7-day Physical Activity report)				X		X	X	X	X	X	X	X**
Standard blood biochemical assessment				X			X		X		X	X**
Dietary assessment (3-day record, intervention only)				X			X		X		X	
Incident outcomes assessment					X	X	X	X	X	X	X	X**

SPPB: Short Physical Performance Battery; MMSE: Mini-Mental state Examination; ECG: electrocardiogram; SARC-F: SARC-F questionnaire: DXA: dual energy X-ray absorptiometry; ICT: Information and Communication Technology: CIRS: Cumulative Illness Rating Scale; PAT-D: Pepper Assessment Tool for Disability: TMT: Trial Making Test: CES-D: Centre for Epidemiological Studies-Depression scale; MNA: Mini-Nutritional Assessment-Short Form; PA: Physical activity; HE: Health economics

* The assessment may be conducted during a second screening visit

** If the participant decides to withdraw from the study an end of study visit (EoS) will be conducted. Every effort will be made in order to collect the largest amount of information about his/her health status. The assessment of the primary outcome will be prioritised. DXA scan will be reacquired if the previous examination has been performed more than 6 months

earlier. All tests and questionnaires will be performed if previous assessments have conducted more than 3 months earlier

7.3.1. 400 Meter Walk Test

The 400-m walk test will be assessed using standardised conditions [51]. A 20-m track will be marked using colour cones. Participants will be asked to walk 400 metres at their usual pace and without overexerting themselves. During testing, they may not use any assistive devices (except for a cane). Subjects will be instructed to begin the test at the start line from a standing position, to walk down the corridor, turn around the cone in a continuous loop, and repeat the course 10 times to complete the 400-metre walk. After each lap, the observer will announce the number of laps completed and the number remaining. During the walk, if participants feel the need to stop and rest, they will be allowed to stand in one place and rest without sitting. They will be instructed to resume walking as soon as they will be able to do so. If they will be unable to continue after a 60-second rest stop or if they need to sit down, the test will be stopped. There will be no limits to the number of allowable rest stops, as long as the participant can complete the walk within 15 minutes without sitting. The test will be discontinued after 15 minutes, a time that corresponds to a very slow walking speed (0.45 m/s), and that translates into a walking capacity that has little utility in daily life. Participants unable to complete the test within 15 minutes will be considered to be mobility disabled. Heart rate will be recorded at the beginning and end of the test. The number, timing, and reasons for the rest stops (fatigue, chest pain, feeling faint or dizzy, shortness of breath, or other) will be recorded. The inability to complete the 400-metre walk has already been used as the defining criterion for mobility disability in major clinical studies, in particular the LIFE pilot and full-scale trials.

7.3.2. Short Physical Performance Battery (SPPB)

The SPPB was developed to objectively measure lower-extremity physical performance [33]. The test takes about 10-15 minutes to administer. The battery has an excellent safety record. It has been administered to over 20,000 persons in various studies and no serious injuries are known to have occurred. The components of the battery are as follows:

- *Standing balance.* For the test of standing balance, participants are asked to maintain balance in three positions, characterised by a progressive narrowing of the base support: with feet together (side by side position), the heel of one foot beside the big toe of the other foot (semi tandem position), and the heel of one foot in front of and touching the toes of the other foot (tandem position). For each of the three positions, participants are timed to a maximum of 10 seconds. Scores are summed for the measure of balance for a range of 0 to 30 seconds.

- *Usual gait speed.* Gait speed is assessed by asking the participants to walk at their usual pace over a 4-metre course. Participants are instructed to stand with both feet touching the starting line and to start walking after a specific verbal command. Participants are allowed to use a cane if necessary, but not the assistance of another person or other devices. At the screening visit, those participants who must use a walker will be excluded. The faster of two walks is used to compute walking speed.

- *Chair stands.* The repeated chair stands test is performed using a straight-backed chair, which is placed with its back against a wall. Participants are first asked to stand once from a sitting position without using their arms. If they are able to perform the task, they are then asked to stand up and sit down five times, as quickly as possible. The time to complete the task is recorded.

Each of the three performance measures is assigned a score ranging from 0 to 4, with 4 indicating the highest level of performance and 0 the inability to complete the test. For the test of balance, participants are assigned a score of 1 if they can hold a side-by-side standing position for 10 seconds but are unable to hold a semi-tandem position for 10 seconds; a score of 2 is assigned if they can hold a semi-tandem position for 10 seconds,

but are unable to hold a full-tandem position for 3 seconds; a score of 3 is assigned if they can stand in a full-tandem position for 3 seconds but less than 10 seconds; a score of 4 is assigned if they can stand in a full-tandem position for 10 seconds. Four categories are computed for walking speed and chair stands, according to standardised and previously published cut-points [35]. A summary score ranging from 0 (worst performers) to 12 (best performers) is calculated by adding walking speed, chair stands and balance scores.

7.3.3. Hand Grip Strength

Hand grip strength is a commonly used measure of upper-body skeletal muscle function and has been widely used as a general indicator of frailty with predictive validity for both mortality and functional limitation [52, 53]. Grip strength is measured in the dominant hand using a hydraulic grip strength dynamometer. If the participant reports current flare-up of pain in the dominant arm, or has undergone fusion, arthroplasty, tendon repair, synovectomy, or other related surgery of the dominant arm in the past 3 months, the other hand should be tested. Other than possible temporary discomfort during the test itself, there are no known risks for the participant.

7.3.4. Pepper Assessment Tool for Disability

Self-Reported Physical Function/Disability is assessed with a modified version of disability instrument that was used in the LIFE-P and LIFE trials, now called the Pepper Assessment Tool for Disability (PAT-D) [54, 55]. The instrument includes 19 items covering 3 domains: 1) basic ADLs (moving in and out of a chair, moving in and out of a bed, gripping with hands, using toilet, dressing, getting in and out of a car, and bathing); 2) mobility (walking several blocks, lifting heavy objects, walking one block, lifting/carrying 5 kg, climbing several flights of stairs, and climbing 1 flight of stairs); and 3) instrumental ADLs (light housework, participating in community activities, managing money, visiting with relatives or friends, using the telephone, and taking care of a family member).

For each item, respondents answer whether they experience 1) no difficulty, 2) a little difficulty, 3) some difficulty, 4) a lot of difficulty, 5) unable to do or, 6) did not do for other reasons. Answers are averaged across the items in order to better assess the overall perceived disability burden by a person.

As in LIFE-P and LIFE, two items will be added to the disability instrument, giving a total of 21 items. These are “walking across a small room” and "walking 400 meters”. These two items have been used previously as the single outcome of interest for studies on mobility disability. In addition, for the basic ADLs (including walk across a room, but not gripping with hands), we plan to ask whether the participant receives help from another person to complete the task. This will allow us to calculate a Katz ADL score [56].

A proxy ADL questionnaire is administered when a participant is not available to complete a follow-up assessment.

7.3.5. Self-reported physical activity

PA (defined as any bodily movement produced by skeletal muscles resulting in an expenditure of energy) will be assessed through an interviewer-administered questionnaire [57]. Subjects will be asked to indicate their average level of PA during three age periods in life: 20 to 40 years, 40 to 60 years, and the past year. One of the following five response categories that incorporates duration, frequency, and intensity of PA could be chosen for each age period: 1) minimal PA, 2) light PA performed 2 to 4 hours per week not accompanied by sweating (e.g., walking), 3) moderate PA performed 1 to 2 hours per week accompanied by sweating or light PA not accompanied by sweating for >4 hours per week, 4) moderate PA performed ≥3 hours per week accompanied by sweating, and 5) physical exercise performed regularly that required maximal strength and endurance

several times per week. This instrument has been used in several cohort studies conducted in older persons, including the "Invecchiare in Chianti" (Aging in the Chianti geographic area; InCHIANTI) [57] and the "Invecchiamento e Longevità nel Sirente" (Aging and longevity in the Sirente geographic area; ilSIRENTE) [58] studies.

7.3.6. Vital signs and anthropometric measures

Prior to randomisation and before the clinical visits, data on sitting blood pressure, heart rate, and main anthropometric measures (including body weight, calf circumference, waist circumference, hip circumference, and mid-arm circumference) will be collected. Body height will be measured once prior to randomisation.

7.3.7. Medication Inventory

Many older adults use both prescription and non-prescription pharmaceutical products. The use of these products is of interest for several reasons. Their use is an important indicator of overall health, and the nature of the drugs taken is a strong indicator of clinically manifest disease. The response to the intervention may be enhanced or diminished by some drugs. Finally, individuals who use nutritional supplements, herbs or other complementary products may have a stronger sense of health self-efficacy, and thus the use of these products could be related to study adherence.

All participants will be asked to bring all prescription and non-prescription medications taken in the past two weeks at each clinical visit. The name, strength and formulation of each product are transcribed. These medications are coded according to formulation for use in subsequent data analyses. This method of drug assessment has been shown to be valid in older adults [59].

6.3.8. ECG

A 12-lead ECG is performed at the initial visit for safety purposes. The ECG is reviewed by the study physician at each field centre to assess potential exclusion criteria. In addition, to assess silent myocardial infarction, ECG will be performed at each follow-up and closeout visit, except that at 3 months.

7.3.9. Cognition

Cognitive function is assessed every year and close-out visit in all participants. Participants with known dementia or who score below 24/30 on the MMSE will be excluded from the SPRINTT trial during the screening visit.

7.3.9.1. Mini Mental State Examination (MMSE)

MMSE was originally proposed by Folstein [60] and is widely used for the assessment of cognition worldwide.

7.3.9.2. Trail Making Test (Part A and B)

The Trail Making Test (TMT) is a test of visuo-motor speed (part A) and executive function (part B) [61]. In TMT-A, participants are asked to draw a line connecting circled numbers from 1 to 25 as fast and accurately as possible. The score corresponds to the time (0–300 seconds) required to connect the circles. In TMT-B, participants are asked to draw a line connecting ascending, alternating, numbers and letters (1-A-2-B-3-C, etc). The score corresponds to the time (0–300 seconds) required to complete the task. The test is stopped if participants cannot complete it in 300 seconds or if five errors are made, in which case, the scores of 300 seconds and five errors are recorded.

7.3.10. Health-related Quality of Life

In SPRINTT, participants' quality of life will be assessed at each clinical visit using the EuroQoL-5D instrument [62-64]. The EuroQoL-5D is a 5-item questionnaire exploring various domains of quality of life. In particular, subjects are asked to describe their current health status in terms of 1) mobility capacity, 2) self-care (e.g., washing, toileting, dressing), 3) usual activities (e.g., work, study, hobbies), 4) pain, 5) anxiety or depression. Each of these domains is described by choosing one out of five possible degrees of impairment/severity. The EuroQoL-5D score is calculated according to algorithms weighted on the European population, adjusting the value of the answers according to social and cultural influences of specific countries. A negative EuroQoL-5D score indicates a quality of life perceived as worse than death.

The EuroQoL instrument also includes a 10-cm long visual analogue scale. Participants will thus be asked to draw a line from a box to the point on the thermometer-like scale (ranging from 0 [worst quality of life] to 100 [best quality of life]) "corresponding to their health-related quality of life".

7.3.11. Center for Epidemiologic Studies Depression Scale (CES-D)

Depressive symptoms will be assessed with the 11-item version of the CES-D [65], which queries about depressive symptoms experienced in the previous week. Scores are transformed using the procedure recommended by Kohout and colleagues [66] to make it compatible with the full 20-item instrument. Total scores range from 0 to 60, with higher scores indicating more depressive symptoms.

7.3.12. Nutrition

Nutritional status of the participant will be assessed using the MNA-SF [67]. MNA-SF is a well-established instrument that allows the rapid assessment of nutritional status in elderly patients in outpatient clinics, hospitals, and nursing-homes. It has been translated into several languages and validated in many clinics around the world. The test is composed of simple measurements and brief questions that can be completed in about 10 minutes.

7.3.13. Sociodemographic, economic and behaviour information

For descriptive purposes, the following participant's characteristics will be collected: age, gender, race, living situation, household composition, marital status, educational level, smoking status, alcohol consumed, employment status, occupation, volunteer work, and income level. Self-rated motivation at adhering to healthy lifestyle will also be measured.

7.3.14. Comorbidity

The Cumulative Illness Rating Scale (CIRS) [68] will be used to measure the chronic medical illness burden while taking into account the severity of chronic diseases.

7.3.15. Screening for sarcopenia

The SARC-F questionnaire has been developed as a possible rapid diagnostic test for sarcopenia [69]. There are five SARC-F components: Strength, Assistance with walking, Rise from a chair, Climb stairs and Falls. The scores range from 0 to 10, with 0 to 2 points for each component. Preliminary studies have suggested that a score equal to or greater than 4 is predictive of sarcopenia and poor outcomes [69].

This instrument will be used in the screening phase in order to avoid as much as possible the DXA assessment in subjects unlikely to be sarcopenic (i.e., SARC-F <3).

7.3.16. Health Care Utilisation

Health care utilisation will be assessed at each clinical visit and all 6-month phone contacts occurring during the follow-up using a self-administered Health Economy questionnaire. This questionnaire will allow the exploration of the following items:

- *Estimation of the cost for running the two interventions.* This will include operating cost, start-up costs (investment in personal computers for the electronic system and maintenance of the systems, investment in exercise training equipment, ICT equipment), variable costs (information technology support, salary for instructors, travelling time costs for home visits), additional time for implementation of the intervention (feedback of a subset of clinicians, counsellors, and ancillary personnel) and aggregate costs (individual component and various clinical and ancillary personnel);
- *Cost-effectiveness analysis.* A comparison of the cost of running the intervention to several effectiveness measures defined by clinicians (e.g., PAT-D, MMSE, SPPB, EuroQoL, etc.) will be conducted;
- *Cost-utility analysis.* This will consist comparison of the cost of running the intervention by quality-adjusted life years (QALYs) (use of the EuroQol-5D questionnaire to compute QALYs).
- *Socioeconomic determinants of physical frailty.* These analyses will be based on economic variables (e.g., monthly income, job status, job category, source of revenue, education level) and complete the already collected demographic information.

Information dealing with potential informal caregivers will be collected using a modified version of the Resource Use in Dementia (RUD) questionnaire [70], adapted to measure the involvement of informal caregivers for physically frail people, and collect additional information dealing with these caregivers (e.g., age, education, marital status, job status, job category, income, and number of children).

7.3.17. Data collection using technological devices

A devoted technological device will be used for data collection in SPRINTT. In particular, an actimeter embedded in the AdamoWatch (a non-medical device) will be used for recording PA levels/variations and eventual fall events throughout the study follow-up. For details, see Chapter 7 “Information and Communication Technologies”.

In addition to the actimetry parameters collected on a continuous basis during the follow-up by the adopted ICT devices (see chapter 7 “Information and Communication Technology”), a 7-day Physical Activity recording will be conducted using an activPAL™ device (Glasgow, Scotland) within 2 weeks from the scheduled clinical visits. The activPAL™ (a non-medical device) is capable of recording for in excess of seven days continuously. The stored activity profile will be retrieved and processed using a PC at the local site. Subsequently, the collected information will be included in the eCRF and centralised in the SPRINTT database.

7.3.18. Standard blood analysis

Part of the blood samples collected at baseline, 12-, 24-, and 36-month visits are sent to local diagnostic testing laboratories for the assessment of main clinical biomarkers (triglycerides, total cholesterol, HDL-cholesterol, LDL-cholesterol (calculated), haemoglobin, complete blood cell count, albumin, ALT, AST, creatinine, calcium, glucose, potassium, sodium, total bilirubin, total protein, urea nitrogen) and vitamin D concentrations.

7.4. Procedures

7.4.1. Bio-bank establishment

Biological samples for future assessment of biomarkers in ancillary studies are collected in the early morning, after an 8-hour fast at baseline, 12-, 24-, and 36-month visits. An operations manual will be produced defining the procedures for collecting, handling, storage, and shipment of biological specimens.

7.4.1.1. Collection of biological samples

In addition to the blood collected for standard analysis and vitamin D determination, a total of 48.7 ml of blood per visit will be collected via venipuncture as follows:

- 2 samples of 8.5 mL serum (with gel) content [gold/orange top]
- 2 samples of 10 mL EDTA plasma [purple top]
- 1 sample of 4 mL lithium heparin [green top]
- 1 sample of 2.7 mL citrate plasma [yellow top]
- 2 samples of 2.5 mL whole blood EDTA

The total amount of blood collected for standard blood work and the establishment of the biobank will be approximately 60 ml per visit.

Urine samples for future assessment of biomarkers in ancillary studies are collected at baseline, 12-month, and 24-month assessment visits. The urine is collected via clean-catch midstream technique. Participants are instructed on the technique and escorted to the restroom where they will provide at least 10 cc of urine into a urine collection cup. Participants will be provided with sterile containers (200 mL).

7.4.1.2. Conditions of sampling

The local technician will immediately bring the biospecimens to the local laboratory where they will be handled and temporarily stored from -20°C to -80°C. In the absence of a -80°C freezer, the stored biospecimens will be retrieved and transferred (without thawing) to the central repository site (University of Göttingem, Germany) at least every month.

7.4.1.3. Coding and labelling procedures

Each tube will have a label reporting the unique ID code of the participant as well as the collection date. The laboratory will be in charge of labelling the tubes with the received ID. A label with code and date will also be applied to the urine biospecimen.

7.4.1.4. Storage

Biological samples are stored at the individual field centres and shipped on a regular basis at the central repository site at the University of Göttingem (Germany).

7.4.1.5. Objective of the bio-bank

Because of the dynamic nature of the field of biomarkers related to PF&S, we have decided not to pre-specify a list of biological variables to be studied. Rather, we have identified biological pathways of special interest for their relevance to PF&S:

- Inflammation (e.g., C-reactive protein, pro-inflammatory cytokines)
- Oxidative damage and antioxidant status (e.g., carotenoids, isoprostanes)
- Muscular anabolism (e.g., pro-collagen-3 N-terminal peptide).

There will be no DNA analysis.

We plan to utilise state-of-the-art analytic approaches to examine these pathways when the biological samples are analysed at study end.

7.4.2. Dual energy X-ray absorptiometry

Total body fat mass and total bone-free lean mass (in kg) will be acquired from total body scans using fan-beamed DXA using standardised protocols [71, 72]. Appendicular lean mass will be derived from the sum of lean mass in both upper and lower extremities. The DXA scanners accepted for the SPRINTT trial are the Hologic (Waltham, MA, USA) or Lunar (Madison, WI) models.

7.4.2.1. DXA operations manual

A DXA operations manual will be produced defining the minimum criteria of adequacy for the DXA scan. Examples of inadequate DXA scan will be provided. The procedures to follow in case of low-quality DXA scan after central reading will be described (e.g., discontinuation of the subject's participation, standardised correction of abnormalities, reacquisition of the scan). Circumstances requiring retraining of DXA technicians will also be defined.

7.4.2.2. Training of DXA technicians

DXA technicians will be trained before the initiation of the RCT and every time the technician is replaced to ensure high-quality data acquisition throughout the study. Technicians will be trained to perform quality control of the recorded data as soon as they are acquired. This will allow repeating the test if needed. Besides regular calibration, the DXA scanner will also be calibrated as soon as a potential abnormality is detected.

7.4.2.3. DXA standardisation

Central reading of DXA results (conducted at the Catholic University of Sacred Heart, Rome, Italy) will be organised in order to limit variability in body composition parameters across study sites. After the participant is randomised based on local DXA reading, the scan output will be read centrally within two weeks. This will allow the reacquisition of the DXA scan should its quality not be adequate. The central reader will regularly provide feedback to local readers in order to guarantee optimal and constant adherence to the RCT protocol. A special phantom for the calibration of the DXA instrument, will be sent once to each study site. All the study sites submitting scans to the central reading unit will complete a separate phantom calibration. It will be necessary to perform this scan before the study start. The data obtained from scanning this phantom will allow the central reading unit to analyse the data from each study site with better accuracy. Specific instruction for performing a scan of the phantom will be included with the phantom when shipped. The data from this scan will be sent back to the central reading unit and the phantom sent to the next study site in line to receive it.

7.4.3. Randomisation

7.4.3.1. Final Eligibility Assessment

Data related to eligibility and key measures will be entered prior to randomisation. A computerized check will be performed to confirm that all required elements are entered and are within range prior to randomization.

Eligibility is dependent on screening data being collected within a set timeframe: all screening data have to be collected within 60 days (i.e., the time between the date of the telephone screening interview and the date of randomisation cannot exceed 60 days), including key clinical and performance measures (400-metre walk; weight). The computerised eligibility check does not permit randomisation if dates of data collection are

outside of these ranges. If a screenee is ineligible, staff will determine whether this may be due toa temporary condition (e.g., blood pressure out of range or too young of age) and discuss this with the participant. Re-screening can be conducted at a later date in such situations.

The allowable time from the date of randomisation to the date of the first individual intervention will be two months. Randomisations will be timed at the clinic sites to allow these deadlines to be met.

7.4.3.2. Randomisation algorithm

Each eligible participant will be randomised to one of the two arms of the clinical trial (MCI or HALE group) using a ratio 1:1, with permuted-block randomisation list through a centralised treatment arms allocation system.

Every time an individual meets the eligibility criteria, the local investigator will send through an *ad hoc* web-based system (see Chapter 7 “Information and Communication Technology”) the following information:

- Study site (for guaranteeing an equal sample size in each of them. Moreover, this is necessary because the cohorts assembled by the centres may differ due to local population characteristics and recruitment plans).
- Participant’s ID;
- Gender, in order to provide an equal distribution of men and women in the two groups;
- SPPB score. The randomisation algorithm will consider an oversampling (i.e., 80%) of participants with SPPB <8 for each SPRINTT trial recruitment site, consistently with results from the LIFE study [17]. This randomisation factor will avoid that the most efficient sites might have an advantage in the recruitment by the earlier inclusion of less frail and less sarcopenic individuals;

Randomisation number and allocation assignment will be attributed by an automated process, and known in real time by the investigators.

7.4.3.3. Masking or Blinding

Masking, which is used synonymously with the term “blinding”, refers to structured attempts to limit the disclosure of study data and participant status to as few persons (both study personnel and participants) as possible. It is generally recommended that access to all types of study data be limited. This includes access to clinic and laboratory measurements, intervention group assignment, and measures of adherence to interventions. Many examples exist in the medical literature to demonstrate that knowledge of some aspects of a participant’s status can subjectively lead to differences in how data are collected and interpreted. For these reasons, the assessment team will be blinded to the intervention assignment.

8. Information and Communication Technologies (ICT)

8.1. ICT Architectural Design

The ICT infrastructure is based on the following components:

- The Communication Hub, centred on the AdamoWatch and deployed in the participant home environment, to monitor his/her physical activity (PA) and collect PA related data;
- The ECRF, SPRINTT Nutrition Frontend, SPRINTT DXA Frontend applications, deployed at the study centres and used by SPRINTT investigators and HealthCare Providers (HCPs);
- The Clinical Knowledge Hub (CKH), the infrastructure component that will

aggregate all data generated by DXA, Nutrition, eCRF and Adamo in a common database. All data collected during the study operations will be transferred and aggregated in the CKH.

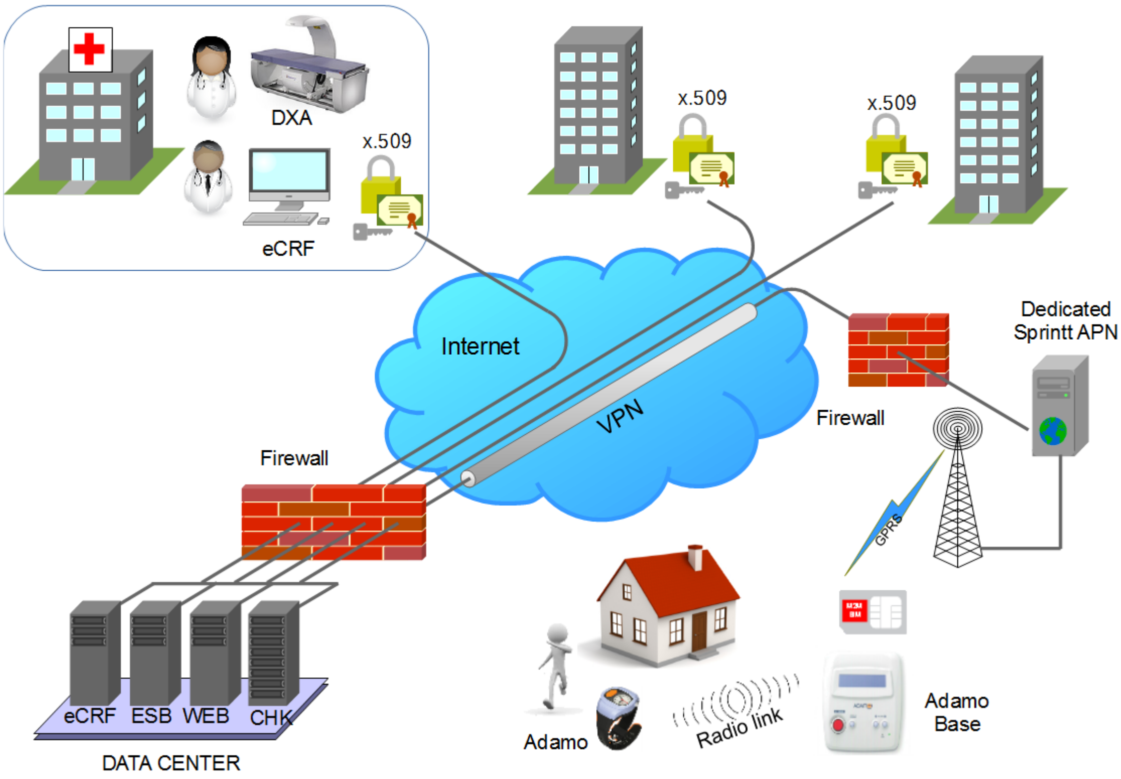

Figure 1. ICT Architectural Design

In order to meet data security, traceability and flexibility requirements, a further infrastructural component is needed. This component is the Enterprise Service Bus (ESB). The ESB governs all communication between modules, therefore it makes possible:

- tracking who is sending data and which data are transferred
- filtering data flow based on the user authorization profile
- managing data encryption
- governing data flow in a centralized way
- decoupling modules in order to reduce each other's dependency.

The ESB will allow easy update of the ICT system design, avoiding rebuilding the existing application when adjustments are needed.

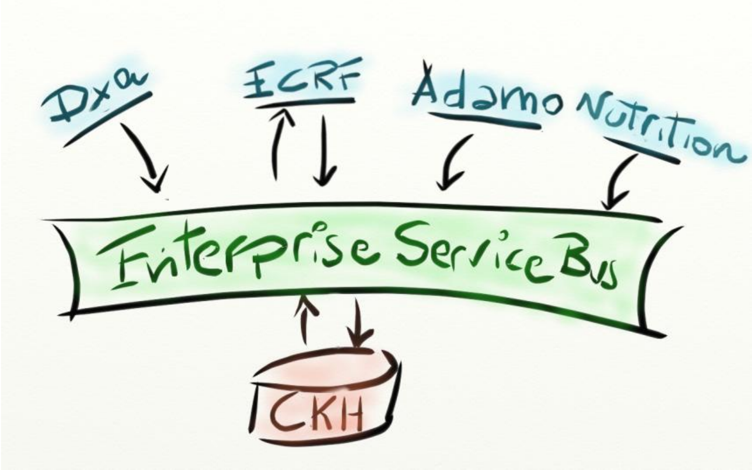

Figure 2. The Enterprise Service Bus

8.2. Clinical Knowledge Hub (CKH)

The Clinical Knowledge Hub is the component of the infrastructure that will aggregate all data generated in the clinical trial. Indeed, the CKH will be able to merge heterogeneous data from different sources [e.g. biological data from lab experiments or recorded by devices, data provided by monitoring devices (Adamo), and data entered through the eCRF (electronic Case Report Form) at the study site or at the participant's home via a tele-monitoring platform.

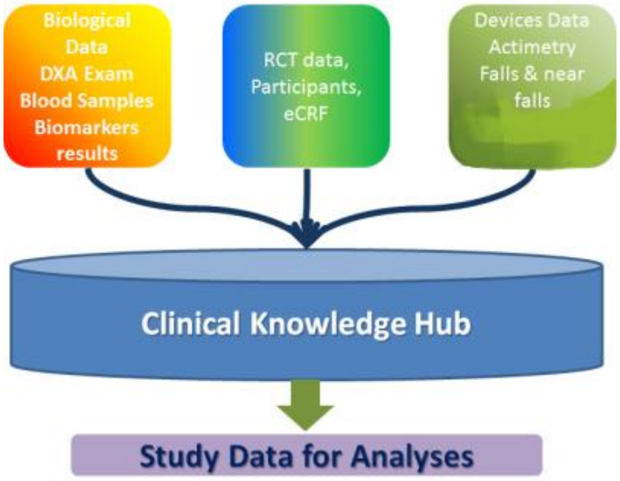

The eCRF and the CKH will be compliant with the requirements of Regulatory Agencies, in particular in terms of user access management, data security and data versioning. All actions will be recorded and documented by audit trail.
All stored data will be exploitable for statistical analyses.

8.3. eCRF

An electronic data capture system will be used for handling clinical data for the study. An electronic case report form (eCRF) will allow recording of all data required according to the protocol and collected by the investigators. The eCRF will be implemented using the CleanWEB™ software, developed by Telemedicine, a web based ICT tool. Cleanweb™ complies with the following standards regarding the treatment of clinical data:

- ICH E6-E9, ICH Q9-Q10,
- 21 CFR part 11, GCP,
- EU Directive 2001/20/EC / Directive 2005/28/EC, Annex 11, cGMP.

Cleanweb™ main features are:

- Data entry by investigators or clinical research technicians (EDC – Electronic Data Capture) with full audit trail,
- On-line help and edit checks of data entry
- Validation and data cleaning,
- Dashboards easy to implement
- Generation of pre-completed documents,
- Automatic generation of reminders & alerts (study timeline dashboard, emails, SMS, DataFAX)
- On-line randomization and stratification
- Project management & monitoring (CTMS – Clinical Trials Management System) including user accounts and access profiles management,
- Generation of descriptive statistics and graphics
- Monitoring reports
- Monitoring of protocol deviations, inclusions, document collection (agreements, consents, etc…) and archiving in the Trial Master File (TMF)
- Investigation centres recruitment & monitoring
- Data management (queries and edit-checks management, double data entry),
- Incremental data extraction (SAS compliant format) etc…

8.3.1. eCRF User support

After a validated training session, CARETEK will be in charge of the First Level Support and TELEMEDICINE will provide a Second and Third Level helpdesk service, via CARETEK reporting. A helpdesk call line will be made available as follows:

- Office hours (9h00 – 18h00 GMT+1). Phone number will be provided at study initiation.
- Outside office hours: on duty personnel (mobile phone).

Additionally, CTK maintains an off-the-shelf platform enabling remote access to a workstation where an issue is reported. This platform may be used to provide a direct service, with prior authorization and under end-user's supervision.

8.4. Software maintenance

Software maintenance includes both maintenance of system source code and maintenance of the eCRF configuration (via the CleanWEB™ Designer). Software maintenance includes both corrective and perfective maintenance.

Corrective maintenance delivers new versions of the software to fix novel bugs and non-compliant use. *Perfective* maintenance delivers new software functionalities or features as resulting from the implementation of the CleanWEB™ Product Road Map.

All software releases are subject to the prior publication of a release note. The Contractor maintains a software development and delivery process fully compliant with the quality assurance requirements applicable to clinical trials.

8.5. Quality Assurance

The eCRF workflow includes a whole quality insurance process ensuring users access of a validated application, through to integrated test phases in the software application development phases.

Quality process applies to software development and to the eCRF set-up.

Quality procedures edition and implementation are based on the GAMP5 methodology and Good Clinical Practices (ICH – GCP).

Delivered documents:

- Users specifications
- Risk analysis and traceability matrix
- Operational qualification protocol
- Operational qualification report

CARETEK will implement the eCRF with support by Telemedicine. CARETEK will train investigator and other designated persons from the investigational centre for the use of the eCRF via Webinars.

Data will be entered in the web-based eCRF directly on line at the investigator’s site by the investigator or by the designated person from their team. The investigator is responsible of the completeness and accurateness of data captured into the eCRF. An internet connection is required in the site.

Data will be automatically transferred via internet from the study centre to the CKH and study database.

The investigator or the designated person from his/her team will agree to complete the eCRF at each participant’s visit, and all other documents provided by the sponsor (e.g. documents related to the clinical interventions.

All data corrections on the eCRF should be made by the investigator or his/her designated person using electronic data clarifications according to the instructions provided. All data modification will be recorded by the audit trail feature of Cleanweb™ software, including date, reason for modification and identification of the person who made the change.

In order to ensure confidentiality and security of data, usernames and passwords will be used to restrict system access only to authorised personnel, whether resident within the investigator’s sites, the sponsor or third parties.

The data manager must make certain that all data are completed on the eCRF and will request correction/clarification from the investigator using electronic data clarifications that should be answered and closed as quickly as possible.

After the participant's last visit, the investigator or co-investigator must attest the authenticity of the data collected in the eCRF by entering his/her user name and password.

After the database lock, the investigator will receive a CD-ROM containing participant data of his/her centre for the study file.

8.6. Data management

Data are collected via the web-based eCRF and stored in a secured database.

For data collected on paper, the investigator or the designated person will be responsible for data processing. All the data collected on paper will be stored in the investigation centre.

The data manager must make certain that all data is completed on the eCRF and will request correction/clarification from the investigator using electronic data clarifications that should be answered and closed as quickly as possible.

For data collected on the eCRF, the investigation centre will be responsible for data processing including data validation performed according to a specification manual describing the checks to be carried out. Based on data validation, previously entered data may require changes. An electronic data clarification form will be sent to the investigator who will be required to respond to the query and make justified changes. All data are

centralised in the DB and all the data transfers will be automatically managed by Service Bus according to a transfer protocol issued by the data manager.

All the following coding activities medical / surgical history, adverse events, procedures and ECG abnormalities will be performed centrally using MedDRA and for medications using WHO-DD.

The coding process will be described in a specification manual.

When data validation is achieved, a *(blind)* review of the data will be performed according to the SPRINTT standard operating procedure. When the database is completed and accurate, it will be locked and the treatment codes will be unblinded and made available for data analysis.

8.7. DXA data flow

To collect DXA files generated by GE-Lunar or Hologic systems (RAW data and PDF) a personalized application will be used for this study (the SPRINTT DXA Frontend application). In order to ensure confidentiality and security of the data, usernames and passwords will be used to restrict system access only to authorized personnel.

This application will be integrated with the Credential Security Provider defined in eCRF (to assure the same security credentials for all the SPRINTT applications in the study) and also with the Clinical Knowledge Hub through the SPRINTT Enterprise Service BUS.

The investigator or the designated person may have secure access to the SPRINTT DXA Frontend application from his/her computer using a web browser and an active internet connection. Once connected, the investigator or the designated person will upload the DXA files (specifying Patient ID, Randomization ID and date of DXA exam) from his/her computer on the Clinical Knowledge Hub.

The information system will check the accuracy and consistency of data and will save the DXA imaging row data on the Clinical Knowledge HUB. Once DXA data is uploaded on the Clinical Knowledge HUB, the information system calculates the DXA Result parameters and sends them to the eCRF through the SPRINTT Enterprise Service BUS. Additionally, a supervisor at the Catholic University of the Sacred Heart (UCSC) will be allowed through a secure access to the SPRINTT DXA Frontend application and could request a secure transfer of specific DXA imaging row data on his/her computer.

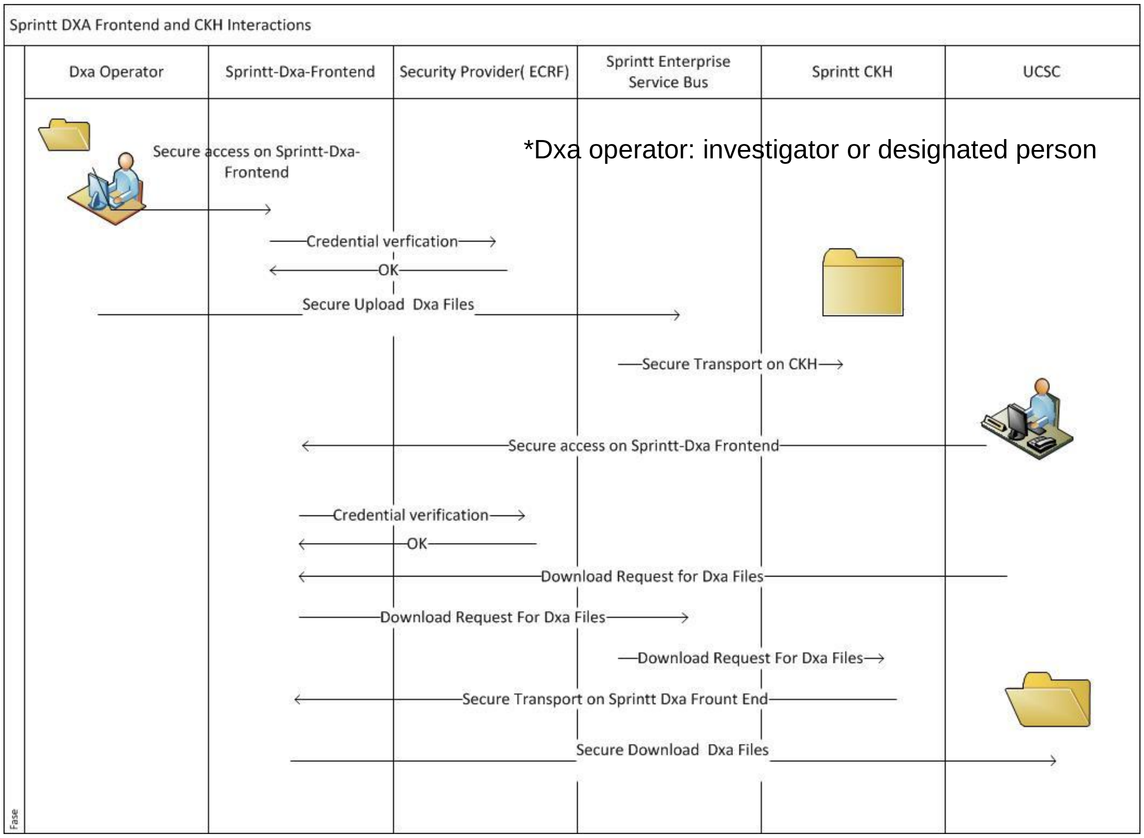

Figure 3. The DXA Frontend and CHK uploading/access

8.8. Nutritional follow-up

Participants in the intervention group will receive regular nutritional counselling from a D/N during the study follow-up. This will also be done having a 3-day dietary record as support of the clinical recommendations that the D/N may provide to the participants randomized to the intervention. Therefore, before each visit, pre-printed and blank forms will be given to participants in the intervention group. In this form, participants will record the diet they followed during the 3 days before the clinical visit. In particular, for any food/beverage taken during the three days, participants will record in writing:

- Time (date and hour)
- Name of food / beverage
- Quantity (portion, weight, volume)

To record the nutritional data a personalized application will be used for this study (the SPRINTT Nutrition Frontend application). In order to ensure confidentiality and security of the data, usernames and passwords will be used to restrict system access only to authorised personnel.

This application will be integrated with the Credential Security Provider in common with eCRF (to maintain the same security credentials for all the SPRINTT applications in the study) and also with the Clinical Knowledge Hub through the SPRINTT Enterprise Service BUS.

The investigator or the designated person may get secure access to the SPRINTT Nutrition Frontend application from their computer by using a web browser and an active internet connection.

Once connected, the investigator or the designated person will compile in English on the Clinical Knowledge Hub the nutrition facts obtained from the participant paper diary, completing with the Patient ID, Randomization ID, the date and the number of visit.

Energy (Kcal) and protein content (g/kg body weight) data are calculated by investigators based on their routine nutritional application, and entered by the investigator in the appropriate eCRF form. In the same way, the UCSC investigator or the designated person will be allowed to secure access to the SPRINTT Nutrition Frontend application and request a secure view of the 3-day nutritional intake diary on their computer to check calculated values and send back notes to the investigator or the designated person in the inclusion centre, as applicable.

All the participant 3-day dietary record forms will be archived in the investigator centre.

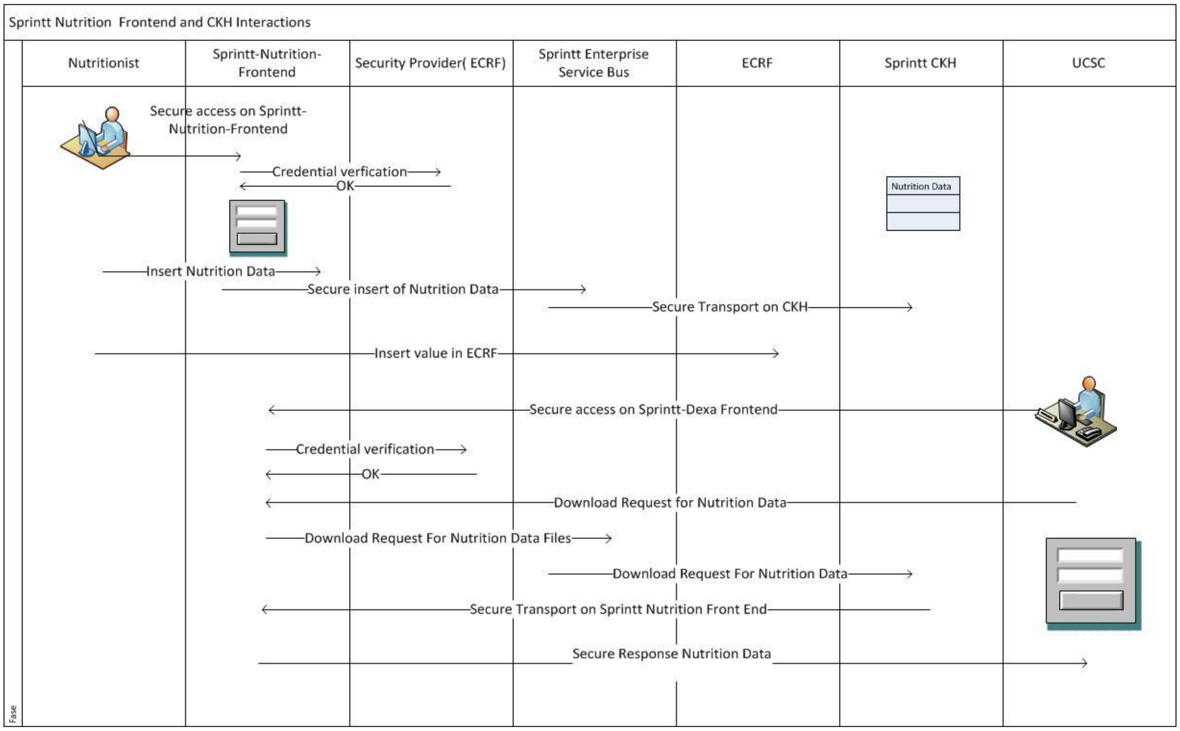

Figure 4. The nutritional intake data workflow

8.9. Biomarkers

All the biomarkers results provided by Consortium partners will be adequately transferred to the CKH in compliance with data privacy regulations. The CKH will store biological results for further bioanalyses requested by the work package dedicated to the bioanalyses (WP8). A specific procedure will be developed including biological samples labels to secure accordance between samples and patients.

8.10. Adamo watch

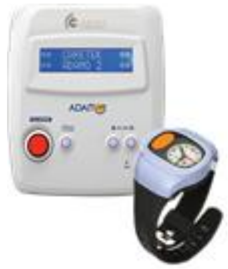

The Adamo watch is a wearable device aimed to acquire patient physical activity (*actimetry*) information during the trials.

Adamo is able to record and transmit data without interfering with the user’s everyday activities. The watch is designed, developed and produced by CARETEK and has been customized in order to specifically fit the SPRINTT clinical trial requirements. The watch is designed according strict criteria of usability and acceptability. No

technical handling or specific interaction with the CHK is required by the participant. The participant is simply expected to wear the device. Although the participant may need to remove the watch, he/she is invited to wear it all the time. The device is water resistant, therefore is not necessary to remove it for washing hands, etc.

Adamo shall be distributed to each training centre in numbered packages, each containing:

- One Adamo watch
- One AC/DC transformer
- One base station already equipped with a machine-to-machine (M2M) mobile sim card.

Each package shall be assigned to a patient after randomization, to be installed at home, following customized written instructions.

Adamo activation is performed by the investigator at the moment the kit is delivered to the elderly participant. The base station operates plugged to a normal electric outlet. The system turns on and automatically registers onto a 2G data mobile network. The watch has to be worn and activated by pushing the main button 4 times. From this moment on, the system is able to acquire the actimetry information of the individual user.

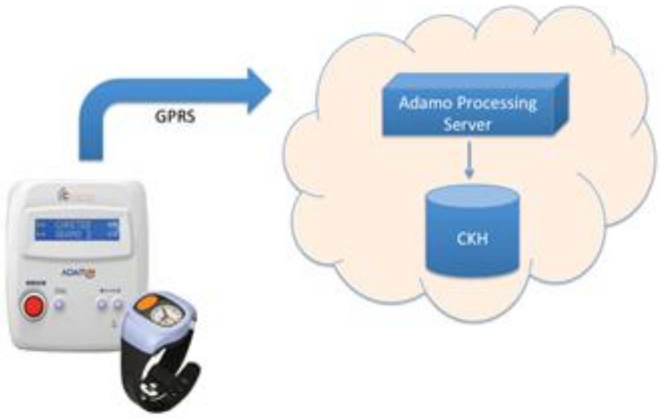

Figure 5. Adamo watch data workflow

More precisely, the watch records raw data from its sensors and process them, updating inside recorded monitoring parameters. Every 10 minutes, it transmits the processed information to the base-station (placed at the participant's home) by means of a short-range radio protocol. If the base station cannot be reached, the watch keeps on updating the monitoring parameters into its own memory and retries a new radio transmission after 10 minutes.

The base station forwards such information to the clinical knowledge hub using the 2G mobile network (GPRS). Recorded data are automatically sent to an intermediate server that adds information exploiting further processing and updating the Clinical Knowledge Hub. No real time monitoring or alarm will be handled, data will be collected and stored to be analysed at the end of the trial.

A Telecom provider, member of the globalm2massociation consortium, will provide the M2M SIM used in SPRINTT. The M2M SIM can connect to any mobile operator in EUROPE also via international roaming. Access to all operators per country will guarantee the best possible mobile network coverage.

Data transfer between the watch and its base-station is encrypted and encoded using the "Idea" algorithm and transferred over the 869.22 MHz frequency band, which is

the European frequency band (869.2 MHz – 869.25 MHz) dedicated to radio terminals for social alarms. Data transfer from the Adamo base-station to the Adamo Server is performed upon a secure TCP socket channel based connection. Data transfer between the Adamo Server and the knowledge Hub is performed using SW tools (Web Services, Syslog, etc.) relaying over a secure data channel.

The DATA transfer from Adamo Server will be performed regularly once à day, all those data will be accessible in the CKH. The data automatically recorded by the watch will include the level of participant's physical activity as well as some outcome measures (i.e. falls events). No geo-localization data will be recorded at any time during the study. All the data will be de-identified, and only the link between the device and the participant's ID will be retained.

Sensors onboard the watch:

**Sensor**	**Measurement**	**Model**
Accelerometer	3 axis acceleration	Analog Devices ADXL346
Barometer	Absolute pressure	Bosch BMP180
Worn watch sensor	Watch is worn or not	Embedded capacitive sensor

Parameters acquired by the watch:

**Monitoring parameters**	**Description**
Packet type	Detect if watch is worn or not
Battery	Battery level
Steps	Number of steps taken since watch activation
Falls	Number of falls detected since watch activation
Mobility index	Regional parameter quantifying the level of activity on five different level: low, medium-low, medium, medium-high, high

8.11. Devices validation

The Adamo device was validated at the SUISM, University of Turin (Italy). Applied validation procedures verified the measurements of the device with respect to:

Walking	A 25 m distance was measured in a quiet hallway in the facility.
Stairs climbing	Participants ascended and descended 19 stairs with 18 cm in height and 30 cm in depth, for a total vertical displacement of 3.42 meters.
Step test	All participants performed stepping trials at three different self-paced speeds: slow, normal, and fast in order: 1) slowly up and down stepping rate; 2) free up and down stepping rate; 3) fast up and down stepping rate.
Fall detection	Participants performed eight different types of simulated falls and activity of daily life (ADL). As it was not appropriate to subject elderly people to simulated falls, the first study involved young subjects performing simulated falls, in a safe controlled environment, under the supervision of a researcher.

A report summarizing all tests performed is available on file.

8.12. Devices deployment

CTK will take care of the Adamo packages quality and manage logistics:

Deliveries of Participant Kits will be scheduled according to a plan taking into account the needs of participant's inclusion by investigational centre and project main objectives:

- Ensuring that each centre has the sufficient number of kits/ devices to be provided to the included participants;
- Availability of a minimum of 2 extra (spare) Adamo packages for allowing kit replacement in the event of hardware failure;
- Avoid the need to store and stock a large number of Adamo packages.
- At the beginning of the inclusion, CTK shall send 12 patient kits to each Centre. More packages shall be provided accordingly to the trial needs: as soon as a training centre supply falls below a defined quantity, CTK will arrange a new stock delivery.

The clinical centres shall act as focal points for deployment and distribution. Maintenance and support will not be handled directly between CTK and each patient, but through the training centres personnel as intermediate. Thus, a technical reference for each centre will be defined and made available.

Each Adamo package will be distributed pre-assembled, configured and checked in order to be "plug and play". In this way, they can be easily managed and distributed, with no extra effort on the part of the training centres personnel.

User and installation manuals shall be provided at the study initiation. They will be easily understandable, readable, with large font. Manuals will be translated in respective national languages.

Each Adamo package is made of an Adamo watch, a base station, an AC/DC transformer and a mobile M2M SIM card.

- A unique ID number flashed into its own nonvolatile memory identifies the Adamo watch. The ID is also reported on the packaging box, thus identifying each watch during shipping and into eCRF.
- Each Adamo base Station is identified by the IMEI code of its own communication module.
- The SIM card is identified by its CLI and UUID code.

Those unique identification codes will be recorded by CTK in the CKH in order to manage and track the logistic activity per center and ensure that each site has enough participant kits for incoming inclusions.

Help-desk telephone support will be provided in English from Monday to Friday 9:00 A.M - 5:00 PM Greenwich time. An e-mail contact will be provided as well for technical support.

8.13. Packages quality assessment

Each involved device shall be validated in CTK by means of a specific and recorded validation process, thus assessing the highest quality of each component.
No sampling check is performed. All devices are individually and specifically tested.

9. Recruitment and Retention

9.1. Recruitment

The recruitment goal of the study is to enrol 1,500 participants, approximately 107 at each of the 14 clinical sites. Participants will be recruited over a 12-month period. It is a recruitment goal of the SPRINTT RCT that at least 1,200 of participants (i.e., 80%) have a

baseline SPPB score of 7 or below. All recruitment related activities will be supervised by the Coordinating Centre located at the UCSC (Rome, Italy). UCSC will also support the study sites in the preparation of recruitment materials.

Each site will develop a site-specific recruitment plan, in order to accommodate the variability across centres in catchment area characteristics, media market outlets, and access to older participants. Recruitment strategies include the use of newspapers, radio and television advertisements, direct mail, and presentations at health fairs, senior centres, medical clinics, and places of worship. Participants of previous studies may also be approached (according to SPRINTT eligibility criteria) and subjects failing the recruitment will be asked to inform acquaintances potentially interested in the study.

9.2. Screening Process

The purpose of the staged screening process is to identify and verify eligible participants over a series of contacts. Interested participants will first be screened by phone or mail. The phone interview/mail questionnaire is designed to exclude individuals who are clearly ineligible or unlikely to benefit from participation in the study. At the first screening clinic visit, medical and functional exclusions will be assessed, including those based on lower extremity physical function. A second clinic visit might be planned if medical information is considered insufficient at the first one, in order to definitively determine the eligibility prior to randomisation.

The exclusion criteria likely to have the largest impact on eligibility will be the score of 10 or higher at the SPPB. The DXA scan will be the last assessment for determining the eligibility of the candidate.

9.3. Retention and drop-out recovery

9.3.1. Identifying Secondary/Proxy Contacts

Although not a criterion for enrolment in the trial, SPRINTT will attempt to identify a proxy respondent for all participants. A proxy respondent and two additional contact persons will be identified and may be contacted to provide supplemental information on the participant.

9.3.2. Retention Promotion Efforts

During the study, participants will be informed about clinically relevant test results.

Before enrolment, preventive measures will be taken to minimise participant's non-compliance related to data collection. Because the study requires a dedicated commitment to examination schedules, only those subjects who fully understand these commitments and appear likely to follow the study protocol will be enrolled. The judgment of the local study staff will be essential in determining overall eligibility with respect to adherence.

Providing clear, easy-to-follow, written instructions about when to return for follow-up visits is important. Reviewing these instructions with the participant periodically during follow-up will be a priority, especially if demonstrated compliance problems exist. Involving the subject's spouse or other family members in these reviews can be useful. Attempts will be made to maintain continuity of follow-up care, so that, whenever possible, the same staff member sees the subject throughout the study. Every attempt will be made to render all clinic visits pleasant. Minimising waiting time and facilitating transportation for the clinic assessment visits, and comfortable waiting room facilities will make the visits more pleasant, thereby enhancing participant retention in follow-up appointments. Gadgets may also be provided to study participants during the study for further enhancing adherence to the protocol.

During the follow-up phase, participants will be contacted over the phone every 6 months (mainly to retrieve information about incident health-related events) and asked to

attend clinic visits every twelve months. If they will be unable to come to the clinic, home or institutional visits as well as phone contacts might be scheduled. Telephone or proxy interviews will be scheduled if in-person visits cannot be completed. Attendance at scheduled visits will be documented by completion of specific data collection forms. Study centres are advised to keep detailed records of rescheduled and broken appointments for each participant. Participant retention will be monitored, and efforts will be made to identify those individuals who need support and encouragement. Records of participants consenting to only a portion of the follow-up procedures, i.e., partial compliance, will also be maintained. Summary reports of such difficulties will help identify problems. Critical review of such problems may offer potential solutions.

9.3.3. Drop-out Recovery Efforts

The following procedures will be implemented (as appropriate in each recruitment site) to carefully document and monitor missed clinic or home visits:

- Preparing for the next visit at the end of each current visit by making the appointment and giving instructions for the next visit.
- Sending out pre-visit reminders (e.g., postcards and phone calls).
- Establishing a mechanism to chart and monitor local clinic attendance, so that clinic staff would be immediately alerted to a missed visit.
- Immediately contacting participants (usually by telephone) when they miss a visit.
- Rescheduling the visit within the same time window, if possible.

Some randomised participants will probably not actively participate in the study, perhaps by not adhering to the intervention and/or not attending the clinic. Regardless of the reason(s) for non-adherence to the study, these participants will be followed until the end of the study, and clinic staff will attempt to make contact every 6 months after the baseline assessment. These contacts will be intended to remind the participant that they are welcome to fully rejoin the study at any time. Considerable effort will be expended to collect main outcome data at appropriate times.

The following guidelines will promote adherence to the protocol, in terms of intervention adherence and clinic attendance. The availability of local clinic resources will determine which techniques should be privileged.

- Participant-staff relationship. A key element contributing to participants’ continued commitment to the trial involves fostering positive, respectful relationships between study subjects and individual members of the staff.
- Continuity of care. In general, participants' appointments should be scheduled so that they can be seen by the same clinic staff members during each visit.
- Clinic environment. The clinic environment which is warm and pleasant, and oriented to the comfort of the participant.
- Participant-staff communications. Good and consistent communication is essential. Instructions are clear and interactions are friendly and individualised. The participant is reminded of the benefits of study participation. Written reminders about clinic appointments further enhance communication efforts. Unmasked clinic staff meets regularly with intervention staff to reinforce the importance of consistency of communications across intervention groups.
- Convenience and accessibility. An easily accessible clinic location, availability of transportation, and convenient clinic hours all serve to facilitate study adherence. Recruitment centres make study visits as easy as possible for participants, a factor critical to the success of the study. All sites take steps to ensure that clinic attendance is not compromised by a lack of transportation, unsuitable hours of clinic operation, or any similar circumstance. If necessary, participants are

reimbursed for or are provided transportation to the clinic assessment visits.

- Time in clinic. Total clinic visit time is kept to a minimum, consistent with maintaining quality. If waiting is necessary, the situation is explained to the participant and, if possible, an offer is made for the participant to see another staff member, or to reschedule the visit. On the other hand, participants are not rushed or made to feel unwelcome. Clinic staff is trained to take time to visit with participants.
- Appointment reminders. Appointment reminders are used to prompt participants to come for clinic visits. These written reminders are mailed to participants so that they receive them one to two weeks before their scheduled visit date.

9.3.4. Monitoring Recruitment and Retention

The Coordinating Centre will routinely monitor screening and recruitment yields, and compare them to preset gender and SPPB score benchmarks for each site. If these benchmarks will not be attained, the main reasons for exclusion of subjects will be analyzed and the recruitment strategies accordingly modified. The Coordinating Centre will also recommend changes in the protocol, if needed. Reports on recruitment will be regularly generated and reviewed by the Steering Committee and the Data and Safety Monitoring Board.

9.3.5. Retention and Efforts to Maintain Contact with Inactive Participants

Retention is promoted by:

1. Examining and attempting to remove barriers (e.g., by addressing parking and other transportation issues, adjusting clinic hours);
2. Incorporating a variety of methods to promote contact with all participants and provide social support for all participants, including those in the HALE control arm;
3. Providing all staff and investigators who have contact with participants with training and regular re-training in motivational methods;
4. Ensuring that participants' concerns are identified and addressed before they express a desire to reduce their involvement in the study.

9.3.6. Efforts to Maintain Contact with Inactive Participants

SPRINTT has the goal of maintaining some form of contact (e.g., phone, e-mail) with participants who are unable to continue full engagement in the study and to foster some form of continued contact (e.g., even an agreement to allow future contact) with participants who are inactive in the study. The greatest importance is given to attending annual assessment visits; even participants who are unwilling to continue attending intervention sessions are strongly encouraged to attend these assessment visits.

9.3.7. Monitoring and Quality Control of Recruitment and Retention

The Data Management Centre (in close relationship with the Coordinating Centre) will collect data to monitor recruitment and retention activities, the number of potential participants contacting each site, how potential participants indicate that they heard about the study, the yield at the various screening steps, and follow-up rates. Regular web-based reports will be available to field centres and the Coordinating Centre. Regular phone contacts between recruitment sites and Coordinating Centre will be maintained in order to:

1. Review recruitment goals and yields for all centres participating on each call,
2. Review the recruitment plan and progress in achieving the objectives outlined in the plan,
3. Share successful and unsuccessful recruitment methods, and

4. Review retention.

If centres encounter difficulties in recruitment, the Coordinating Centre will propose a graduated set of assistance responses based on the degree of recruitment shortfall. The possible inclusion of pre-selected back-up recruitment sites will be considered in the presence of major immitigable difficulties a centre might experience. Ad hoc amendment will be submitted in this case.

10. Sample size considerations and statistical analysis

10.1. Sample size considerations

The primary efficacy endpoint is the time from randomization to the date of first occurrence of inability to complete the 400-m walk test in less than 15 min.

Sample size calculation is based on the LIFE study database. Specifically, survival analyses were run according to different baseline levels of SPPB score (lower than 8 vs. 8 and 9) for the primary endpoint. As depicted in Fig. 6, the effect of a long-term structured PA programme on major mobility disability was negligible in participants with baseline SPPB score ≥8 (hazard ratio = 0.94). Conversely, the hazard ratio was clinically and statistically significant in enrolees with baseline SPPB <8 (hazard ratio = 0.75; 95% CI [0.59;0.94]; p=0.012).

A total of 434 events are required to provide 85% power to detect a 25% reduction in the hazard of major mobility disability using a log-rank test performed at a two-sided alpha level of 5%. In order to achieve the 434 targeted events, 1200 subjects with baseline SPPB <8 will need to be randomised over a 12-month accrual period, with a maximal follow-up time of 3 years and a common exponential dropout rate of 25% over two years. The inclusion in the SPRINTT RCT of participants with a baseline SPPB score of 8 and above would decrease the study power and, hence, the probability of success of the trial. On the other hand, restricting the enrolment to older people with SPPB <8 would limit the aim of study to characterise the whole spectrum of PF&S. Hence, a convenience sample of 300 older adults with baseline SPPB 8 or 9 will be included in the study. An ad hoc hierarchical testing procedure has been formulated to preserve the study power (see below).

Three different plans have been developed, based on recruitment efficiency, incidence of events and length of follow-up. A blinded sample size reassessment based on number of events is planned after 11 months from the beginning of recruitment to ensure that 434 events will be reported at the end of study (3 years after the first study participant has been enrolled). This interim evaluation will allow taking immediate actions to preserve the study power in case the efficiency of enrolment or the number of observed events is not coherent with the original assumptions.

1) Scenario 1: recruitment starts at a rate of 125 participants/month (8-9 participants/centre/month). Based on a log-rank test with a 5% two-sided alpha level, a sample size of 1,500 participants (750 per treatment arm; 80% participants with SPPB <8 and 30% with SPPB >30%), with a total number of 35 events at 11 months and 43 at 12 months, will provide 83% power to detect a 30% reduction in the hazard rate over 24 months of follow-up (247 events).

2) Scenario 2: recruitment progresses as outlined above, but the number of events recorded at month 11 is lower than expected. A follow-up extension (up to 36 months) should assure that the study power is preserved (at least 80%) to detect a 30% reduction in the hazard rate with a sample size of 1,500 participants. An additional power reassessment may be performed 12 months after the end of recruitment in order to establish the optimal length of follow-up, based on the number of events recorded thus far.

3) Scenario 3: recruitment efficiency is suboptimal at the very beginning and

increases over time (from 6 to 160 participants/month). The duration of the recruitment will be extended by 6 months and the follow-up of participants enrolled earlier continued beyond 24 months (the follow-up duration initially planned), up to 36 months, which will be maintained as the maximum duration of the study. A sample size of 1,500 participants will provide 85% power to detect a 30% reduction in the hazard rate, with an expected 281 total number of events at the end of the trial. At the 11-month interim evaluation, the number of enrolees is expected to be around 475, with around 9 events recorded.

Figure 6. Effect of a long-term structured PA programme on the onset of major mobility disability in older persons with baseline SPPB score ≥8 (A) or <8 (B).

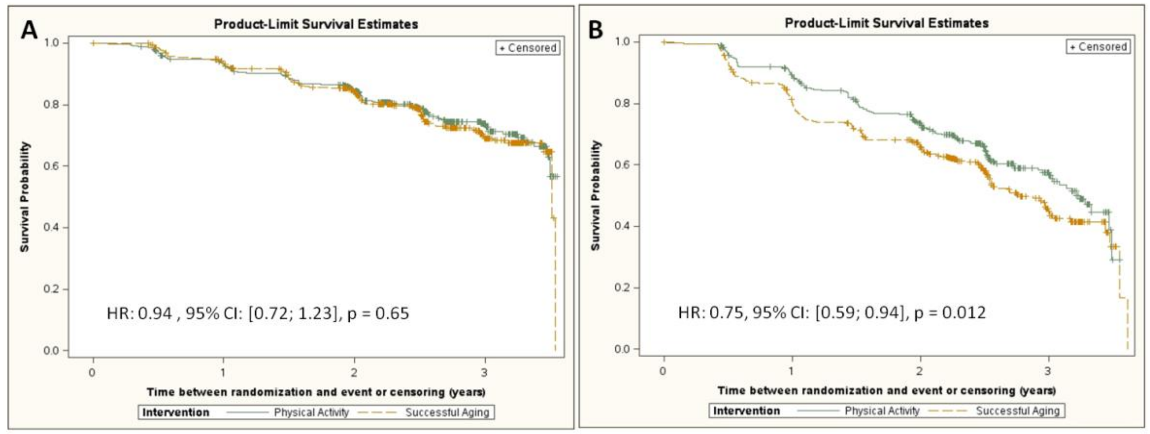

10.2. Statistical analysis

Analyses of intervention effects will be based on the intent-to-treat principle. This implies that data from participants allocated to a treatment group will be analysed as part of that group irrespective of their compliance with treatment.

First approach analyses will always be dedicated to the evaluation of the subsample of participants with SPPB <8 (in agreement with results from the LIFE study and the present sample size calculations). Secondary analyses will subsequently be conducted considering the overall sample of the SPRINTT population (i.e., n=1,500) as well as the 300 participants with SPPB ≥8.

All tests of significance will be two-sided with a maximal type I error risk of 5%.

10.2.1. Primary efficacy endpoint

The primary comparison of intervention groups with respect to the distribution of time until the first post-randomisation occurrence of the primary outcome will be based on log-rank stratified by study site and gender. The primary comparison will be conducted only in study participants with baseline SPPB <8. If this primary analysis is statistically significant ($p<0.05$), the comparison will be extended to include the remaining 300 participants with baseline SPPB 8-9. An additional binary indicator variable will be introduced denoting a baseline SPPB score <8 or ≥8 and its interaction with the intervention group. Hazard ratio and confidence interval will be computed for the whole population only if this interaction term can be ignored (i.e., if it is not statistically significant). The hazard ratio between intervention groups and the corresponding confidence interval will be computed using a Cox proportional hazard model with site and gender as co-variables for comparison in subjects with a SPPB score <8 and by adding

the SPPB score ≥8 flag and corresponding interaction term as co-variables for the comparison in the whole study population.

The time to event is defined as the time from randomisation to the date of first occurrence of disability. Participants who do not meet this criterion will be censored at the time of their last primary outcome evaluation. In secondary analyses, in order to take into account additional covariates, we will use a Cox proportional hazards model if the underlying assumptions appear warranted. The proportional hazards assumption will be verified by testing the treatment-by-time interaction in the Cox model.

10.2.2. Secondary efficacy endpoints

For the assessment of secondary efficacy endpoints, specific models will be used for continuous outcomes (e.g., changes in physical performance, nutritional status, functional status, cognitive function, and quality of life). Specifically, changes over time between groups will be assessed by a repeated-measure mixed model with terms for intervention, time, baseline score, baseline score by intervention interaction, and time by intervention interaction. For outcomes such as incidence of falls and mortality rate, the analysis defined for the primary efficacy outcome will be replicated (except for the secondary analysis with competitive risk for the mortality rate). Additional analyses (e.g., subgroup composite endpoint analyses) will be defined in agreement with the Managing Board and the Data Safety Monitoring Board and specified in the statistical analysis plan that will be produced prior to the beginning of the analyses.

Please note that within this section, we have limited the description of the analysis plan to the major SPRINTT hypotheses. Nevertheless, the relevance and amount of the data collected during the project will certainly lead to ancillary and secondary analyses always within the scopes and objectives of SPRINTT. Such secondary plans will be developed after formal approval and validation by the Managing Board of SPRINTT.

11. Safety considerations

11.1. Participant Safety and Confidentiality

The study monitors the medical safety of participants. One aspect of this monitoring is to evaluate potential participants at screening to determine whether it is safe for them to participate in the planned interventions. Another aspect is monitoring of safety during study assessments. A third area is safety during PA, both supervised and unsupervised. Also, if a volunteer has a medical or surgical illness, the safety of continuing or resuming participation in interventions is ascertained by the medical staff at the local centre in cooperation with the participant’s primary care physician. Finally, the study monitors adverse events, assesses their potential relationship to the intervention and reports events to the Data Safety Monitoring Board (DSMB).

11.2. Data Safety Monitoring Board

A DSMB is established with responsibility to monitor and manage the safety aspects of the study. Local study physicians report to the DSMB for issues related to participants safety.

The DSMB has the following charges:

- Review the study protocol with regard to recruitment, randomisation, intervention and subject safety, in order to identify the relevant data parameters and the format of the information to be regularly reported.
- Review data (including masked data) over the course of the trial relating to intervention effects and subject safety.
- Identify problems relating to safety over the course of the study and inform study PI

via written report, who, in turn, ensures that all local PIs receive this report.

- Identify needs for additional data relevant to safety issues and request these data from the study investigators.
- Propose appropriate analyses and periodically review developing data on safety and endpoints.
- Make recommendations regarding recruitment, intervention effects, retention, compliance, safety issues and continuation or temporary arrest of the study, necessary amendments.
- Send the Directorate and local PIs written reports following each DSMB meeting. These reports may address all (blinded) issues reviewed by the DSMB. The PIs then send the DSMB report to their respective Ethics Committees. The study PI is responsible for sending the reports to individual site PIs, who in turn are required to distribute the report to their local Ethics Committees.

At any time, the DSMB may recommend discontinuation of any component/intervention group of the study for any of the following reasons:

1) Compelling evidence from this or any other study of an adverse effect of the study intervention(s) that is sufficient to override any potential benefit for the interventions to the target population.
2) Compelling evidence from this (or any other) study of a significant beneficial effect of the study intervention(s), such that its continued denial to other study group(s) would be unethical.
3) A very low probability of addressing the study goals within a feasible time frame.

The DSMB may convene an executive session at any time. The study Management Board and Institutional Review Board will make the final decision on whether or not to accept the DSMB’s recommendation about discontinuation of any component of the study. Any serious adverse events that might be due to the study intervention are reported to the DSMB, the Institutional Review Board, and to the SPRINTT Management Board.

11.3. Medical Problems Detected During the Study Assessments

Medical problems that increase risk of study participation are assessed through structured telephone interviews and in-person physical examinations during the initial subject evaluation, prior to randomization. The goal of these assessments is to detect conditions by history, such as recent major surgery, symptomatic conditions such as angina or weight bearing pain and asymptomatic conditions, such as valvular heart disease or abdominal aortic aneurysms. Such persons are excluded from further participation and are referred to their primary care physician for further care.

11.4. Safety Considerations for Study Assessments

All study assessments are done by trained and certified staff. Safety precautions are taken during the 400-metre walk test by applying standardized stopping criteria. If the participant reports chest pain, tightness or pressure, significant shortness of breath or difficulty breathing, or feeling faint, lightheaded or dizzy the test is stopped. During the 400-metre walk tests a semiautomatic defibrillator is available. Onsite staff is trained to provide basic life support and to provide immediate care when faced with medical emergencies. Also, institutional and community Emergency Medical System (EMS) services are activated if needed.

It is anticipated that some medical problems might occur during the course of the study while some participants are in the clinic. The following is a summary of a plan of action based on level of acuity of the problem.

Emergent problems and problems that are life threatening or require life saving

attention should be dealt with using the local EMS. Clinical staff may provide basic life support as an interim measure when appropriate until EMS personnel arrive. All personnel involved in the assessment of physical function and administration of the PA intervention will receive cardiopulmonary resuscitation (CPR) training, according to the BLS-D protocol. The certificate of BLS-D training will be kept by the local PI and renewed annually. The study staff is responsible for notifying the participant's family or designated contacts and the participant's primary care provider.

Urgent medical problems and problems that require immediate attention but that do not require life-saving attention are dealt with by taking measures to ensure the participant's comfort and offering first aid, as appropriate. Disposition plans should be made with the participant, clinic staff, investigators, family, and primary care provider. The clinic staff may arrange transportation of the participant to another medical care site for definitive care. The primary care provider and family or designated contacts should always be notified.

General medical problems or those problems that require attention when feasible should be dealt with by contacting the primary care provider. The clinic staff should follow the primary care provider's directions regarding disposition and follow-up. The participant should be advised regarding the primary care provider's instructions and documentation of the problem and actions should be placed in the participant's record on a progress note. A follow-up letter to the primary care provider documenting the problem and actions taken should be sent by clinic staff.

There are several types of alerts in SPRINTT. The table below describes a summary of alerts and the appropriate action.

ALERT	ACTION
Blood Pressure<br>SBP > 140mm/Hg or<br>DBP > 90mm/Hg	Clinic staff inform the participant
Blood Pressure<br>SBP > 170mm/Hg or<br>DBP > 100mm/Hg	Qualified staff should talk to participant, and encourage participant to seek additional follow-up and/or evaluation.
Resting Pulse<br>Rate > 100 or < 40 beats/min	Qualified staff should talk to participant, and encourage participant to seek additional follow-up and/or evaluation.
Abnormal results at the Center for Epidemiological Studies-Depression Scale and/or Mini Mental State Examination questionnaires	Qualified staff should talk to participant, and encourage participant to seek additional follow-up and/or evaluation.
MNA-SF score <8/14 (identifying malnutrition)	The result will be forwarded to the participant's PCP regardless of group assignment. For participants randomised to the multi-component intervention, personalised nutritional recommendations will be elaborated and followed up by the local D/N with support from the study physician
ECG meets exclusion criteria.<br>Serious conduction disorder (e.g., 3rd degree heart block), uncontrolled arrhythmia, or new Q waves or ST-segment depressions (>3 mm) on ECG.	Qualified staff should talk to participant, and encourage participant to seek additional follow-up and/or evaluation.
No posterior tibial arterial signal present in either leg	The participant will be examined by a qualified staff member who will evaluate the participant for

	signs of critical limb ischemia. The qualified staff member will re-check for presence of a posterior tibial artery signal and will check for presence of a dorsalis pedis arterial signal.<br>Appropriate follow-up will be recommended. If critical limb ischemia is determined to be present, the study physician will be notified.
Serum glucose <60 during intervention session in diabetic on hypoglycaemic agent	Intervention staff provide immediate care such as offering beverage with sugar and advise participant to contact primary care physician regarding adjustment of hypoglycaemia medication

The following cut-points will be used to determine abnormal values for blood tests:

**Test**	**Value out of reference range**<br><br>*Participant should be notified at a routine visit or within two weeks by a qualified staff member that the value is out of the normal range for all adults but may or may not be medically important and could be discussed with primary care physician*	**Value may be medically significant**<br><br>*Participant should be notified at a routine visit or within two weeks by a qualified staff member that the value is potentially medically significant and strongly recommend that it be discussed with primary care physician*	**Value requires immediate notification**<br><br>*A qualified staff member should contact the participant within one working day and request permission to contact a primary care physician. Assuming that the participant has normal vital signs and is feeling well, there is no need to invoke emergency medical systems*
Triglycerides (mg/dL)	>199		
Total cholesterol (mg/dL)	>199		
HDL (mg/dL)	<40		
Haemoglobin (g/dL)	M <13 or > 16<br>F <12 or > 15	M < 12 or > 16.5<br>F < 11 or > 16	< 8 or > 18
WBC count (/mm^3)	< 4000 or >11000	< 3000 or > 12000	< 2000 or > 20000
Platelet count (/mm^3)	< 130000 or > 400000	< 100000 or > 500000	< 30000 or > 1000000
Sodium (mEq/L)	< 135 or > 146	< 130 or > 155	< 125 or > 160
Potassium (mEq/L)	< 3.5 or > 5.3	< 3.0 or > 5.7	< 2.6 or > 6.2
Calcium (mEq/L)	<8.5 or > 10.3	< 8.0 or > 11.5	< 7.0 or > 13.0
Glucose (mg/dL)	< 70 or > 125	< 60 or > 140	< 50 or > 400
BUN (mg/dL)	> 30	> 40	> 80
Creatinine (mg/dL)	> 1.4	M >2.0<br>F > 1.6	> 3.5
Albumin (g/dL)	< 3.5	< 3.0	
Vitamin D (ng/mL)	< 30		

11.5. Safety Considerations for the Physical Activity Intervention

Appropriately designed and implemented PA interventions have been shown to be safe and efficacious in older adults. The literature on PA training in the frail elderly in nursing homes contains no reports to date of serious cardiovascular incidents, sudden death, myocardial infarction, or exacerbation of metabolic control or hypertension. A recent review concluded that an appropriately prescribed resistance PA program is a safe form of PA for the majority of the population and is associated with minimal risk of cardiovascular events, even in those with previous myocardial infarction or chronic congestive heart failure. Furthermore, the recently published LIFE study reported no differences in serious adverse events between participants on PA intervention and those randomised to a successful aging educational programme [17]. This latter evidence, coupled with the documented efficacy of the LIFE exercise protocol, prompted the SPRINTT Consortium to adopt an exercise program very similar to LIFE.

11.6. Pre-Physical Activity Safety Screening

To maximise the participants' safety, we follow a standardised screening protocol (Figure 7). Accordingly, all potential participants undergo screening for cardiovascular and other major diseases by means of a health questionnaire, medication inventory, ECG and physical exam, which are initially collected and reviewed by a qualified assessor. In cases where there are no potential alert values on any of the forms and no unexpected or unusual symptoms or conditions, the study physician may approve performance of the 400-metre walk. In all other cases, the study physician must review and approve the participant for performance of the 400-metre walk. Those with overt cardiovascular diseases (or other severe diseases) that meet the exclusion criteria as determined by the study physician are excluded. Prior to randomisation of a potential participant, all medical information must be reviewed by a study physician, who is ultimately responsible for determining study eligibility and approving randomisation. Next, otherwise eligible persons undergo the 400-metre walk test. According to a protocol to evaluate cardiovascular reserve similar to the one suggested by Gill and colleagues [73], persons who develop chest pain or substantial shortness of breath during the 400-metre walk test are also excluded. Those who are not excluded are randomised to the MCI or the HALE group.

11.6.1. Reasons for not performing physical activity stress testing.

Participants do not undergo PA stress testing. This decision is based on the following considerations:

- The recommendations published by Gill and colleagues [73] advised that a screening protocol based on a simple cardiovascular reserve test (400-meter), is more suitable for screening older adults than a protocol based on stress PA testing.
- The American Heart Association (AHA) and the American College of Sports Medicine (ACSM) joint position statement advised that "apparently healthy persons of all ages and asymptomatic persons at increased risk may participate in moderate-intensity physical activity without first undergoing a medical examination or a medically supervised, symptom-limited physical activity test" [74]. In this context, SPRINTT participants will not exceed moderate intensity (Borg scale=13) as per protocol.
- The AHA Scientific Statement on Exercise Standards for Testing and Training by Fletcher et al. [75], advised that "for older, apparently healthy persons desiring to participate in a low to-moderate intensity activity such as walking, an exercise test may not be required", and that "the role of exercise testing among the elderly (>75 years) as a guide to identifying the high-risk patient for primary prevention requires further study".
- The majority of older persons (>75%) are unable to satisfactorily complete a treadmill

exercise test [76], which makes its utility as a screening tool in the elderly population questionable.

- Older persons have a high prevalence of ECG abnormalities (e.g., left bundle branch block) [77], which diminish the diagnostic accuracy of treadmill exercise testing.
- Participants with potential cardiac contraindications to the PA programme are identified and excluded by means of the screening process described above.
- PA of moderate intensity is conducted in a supervised environment.
- A maximal or near maximal exercise test on a treadmill is an unpleasant for sedentary and unfit adults. Requiring an exercise stress test may deter older persons from participating in the trial.
- Regular exercise and PA may actually reduce the overall risk of MI and death among older persons [78, 79], possibly through improvements in cardiac risk factors and overall fitness [80].

In summary, exercise stress testing provides little additional information, is not necessary to protect the safety of participants, and is disliked by sedentary and unfit participants. The PA intervention protocol also requires that the centre-based sessions at the beginning of the study include careful monitoring of cardiac and other signs and symptoms by trained staff.

11.7. Safety Measures during Physical Activity

Centre-based interventions are conducted at a central location and all sessions are conducted and supervised by trained interventionists, who monitor potential adverse experiences and symptoms. During the PA sessions, a defibrillator and on-site trained staff are available to deal with medical emergencies. Also, institutional and community EMS services are activated if needed. As indicated previously, participants are taught the importance and proper method of warming-up prior to and cooling-down following structured activity sessions. If at any point during a PA session, participants develop chest pain, shortness of breath, or dizziness, they are instructed to rest and to contact the centre and their physicians if these symptoms persist or recur with further PA. The implementation of the PA sessions is consistent with the recommendations published by Fletcher and colleagues [75] for older adults who may have stable cardiovascular disease.

Blood pressure and heart rate are monitored before and after the walking activity at each centre-based intervention session. Blood pressure and heart rate are measured every 5 minutes during walking at each centre-based session in participants who had experienced any of the following at a previous physical activity session:

- Resting blood pressure systolic ≥200 mm Hg or diastolic ≥100 mm Hg
- Decrease in systolic blood pressure ≥20 mm Hg following the activity
- Increase in systolic blood pressure to ≥250 mm Hg or in diastolic blood pressure ≥115 mm Hg following the activity
- Resting heart rate ≥120 beats/min or ≤45 beats/min
- Increase in heart rate ≥90% of age predicted maximum
- Unusual or severe shortness of breath
- Chest pain or discomfort, or heartburn
- Palpitations
- Light headedness, dizziness or feeling about to faint
- A PA session had to be discontinued because of other symptoms, excluding musculoskeletal symptoms (e.g., knees, ankles, hips), reported by the participant.

If any of the above occurs, the individuals are instructed to seek their physician's permission before continuing with the PA programme.

Very few persons are expected to drop out for this reason based on previous experience

(e.g., LIFE study) and cardiac-based exclusion criteria.

Procedures to minimise discomfort include warm-up and cool-down activities that include light walking or cycling and flexibility exercises. The participants are also introduced to the intervention activities in a structured way, such that they begin with lighter resistance and gradually increase over the course of the first 2-3 weeks of the intervention. During the intervention visits, participants are supervised at all times and instructed on correct PA techniques. Participants are instructed to talk with the interventionists about any muscle soreness.

If for any reason the participant reports an injury, chest pain, shortness of breath, or dizziness, they are referred to their doctor, or the study clinician calls the doctor or other health care provider. In addition, specific criteria for suspending or stopping PA are developed to adjust the program for intercurrent illness.

11.8. Safety considerations for DXA examination

As part of DXA evaluations, subjects are exposed to radiation. The maximum radiation dose for the total body measurement will be approximately 5 mrem, the amount a person receives during 5 days of normal background radiation. The overall radiation exposure by DXA during the whole trial will be much lower than one single standard chest X-ray. Most institutions have radiation safety committees that review clinical protocols in which subjects are exposed to any amount of radiation. Each institution has different requirements that need to be met. It is the responsibility of the site to meet all the local requirements to conduct these tests.

11.9. Adverse Events

Serious adverse events in SPRINTT are defined to include: death, a life-threatening adverse experience, inpatient hospitalisation, a persistent or significant disability/incapacity, or a clinically significant laboratory or clinical test result. Important medical events that may not result in death, be life-threatening, or require hospitalisation may be considered serious adverse experiences if they might jeopardise the participant or might require medical or surgical intervention to prevent one of the outcomes in the definition. An example of this in SPRINTT is an injurious fall resulting in a fracture that occurred during walking for PA.

In SPRINTT, an adverse event or experience is defined as any health-related unfavourable or unintended medical occurrence that happens during the process of screening or after randomisation. Certain adverse events may be protocol-defined outcomes (serious fall injury). Minor adverse events are defined as conditions that may be unpleasant and bothersome to the participant, such as sore muscles, but that do not require discontinuing the study intervention or components of the intervention. Examples of minor adverse events include but are not limited to the following: anxiety, fatigue, decreased appetite, insomnia, dizziness, muscle or joint stiffness, muscle strain or soreness, ankle or knee pain, foot pain, and other minor symptoms that may have restricted the participant’s usual activities for at least ½ day like a head cold, flu or allergy problems. Minor adverse events should be reported on an annual basis to each site’s own Ethics Committee.

Potential adverse events for study related activities and interventions are explained to each participant by trained study personnel during the informed consent process. Each participant is instructed to report the occurrence of an adverse event at scheduled data collection times (scheduled clinical exams or phone interviews). Participants also have access to study clinic personnel at other times to report serious adverse events or concerns about the safety of participating in the SPRINTT study.

Expected serious adverse events related to the exercise intervention include in

rare instances, heart attack, stroke, and death. Cardiovascular events are assessed using standard protocol measures including ECGs. When a cardiovascular event has occurred, the study physician decides whether it is permissible for the participant to continue interventions. If the SPRINTT interventions are discontinued for safety reasons, they may be resumed after consultation with the participant's primary care physician.

Serious fall injuries and fractures are assessed using standard protocol measures, including radiographs and hospital records. When a serious fall injury or fracture occurs, a study physician decides whether it is permissible for the participant to continue interventions. If the SPRINTT interventions are discontinued for safety reasons, they may be resumed after consultation with the participant's primary care physician.

In the SPRINTT safety monitoring system, participants who report adverse events to any staff person at any time are referred to unblinded medical staff responsible for identifying, recording, and managing these events. Safety-related events are reported in a timely fashion as required by the DSMB and the Ethics Committee responsible for the study. Interventionists and other staff reporting or managing adverse events for safety purposes do not at any time communicate information regarding these events to study assessment personnel.

SPRINTT maintains an event outcome record that is completely separate and distinct from the safety monitoring system for the intervention group. This is necessary because many of the SPRINTT staff members are not masked to intervention assignment, and it is critical that the identification and reporting of serious adverse events for safety reasons not bias the study's collection of outcome data. Thus, for outcome purposes, all SPRINTT participants are systematically queried at clinic visits or phone calls according to the protocol to capture outcome data on study outcomes, medical events, or adverse experiences. This separate outcome record contains solely those adverse events that are reported through these interviews conducted by designated outcome assessment staff masked to intervention assignment.

All serious adverse events and deaths shall be reported within 48 hours to DSMB and to the Management Board. All Serious Adverse Events shall be reported in a summary format by the blinded treatment arm (A and B) to the DSMB and Management Board monthly.

11.10. Confidentiality

The information below relates to all collaborating performance sites for the study. Data are used only in aggregate and no identifying characteristics of individuals are published or presented. Results of testing are shared with the participant's private physicians if participants agree to this. Alert values for all medically relevant procedures (e.g., ECGs) are developed, and a system is in place to alert study physicians and participants' private physicians, depending on the urgency of the values.

Confidentiality of data is maintained by using research identification numbers that uniquely identify each individual. Safeguards are established to ensure the security and privacy of participants' study records. The information collected from participants in this study has a low potential for abuse, since the data do not address sensitive issues. Nevertheless, appropriate measures are taken to prevent unauthorized use of study information. The participant ID is used. The research records are kept in a locked room in the study centre. The files matching participants' names and demographic information with participant ID are kept in a separate room and are stored in a locked file that uses a different key from that of all other files. Only study personnel have access to these files. After the study is completed, local data are stored with other completed research studies in a secured storage vault.

In compliance with national and EU regulations, SPRINTT accesses personal

health information and medical records only after receiving signed informed consent. Participants' medical records are obtained, reviewed and abstracted. Such records are kept in a locked cabinet that is separate from other files cabinets and that uses a different key from that of all other files.

12. Mitigation plans

Five main risks may threaten the successful accomplishment of SPRINTT:

- *Insufficient recruitment of participants.* The enrolment of participants at each centre will be closely and regularly monitored by the Coordinating Centre via intranet, site visits, and conference calls.
  If a centre reports slow recruitment, the first mitigation measure will consist in identifying and putting in place additional sources of screening in the area. If the local counteraction fails, the other centre in the same European region may be involved at supporting the recruitment (e.g., by enrolling some extra participants). If this mitigation procedure is still inadequate, an additional recruitment site might be involved for backing-up the activities. Several potential back-up sites have already been identified throughout Europe and expressed their willingness at being involved if necessary. If the recruitment lags in multiple centres, an amendment to modify inclusion criteria might be discussed within the SPRINTT Managing Board, together with IMI representatives. If agreed, the amendment will be submitted to the Ethical Committees, and then implemented in the RCT.
- *Low adherence/compliance to the intervention protocol*. A critical issue in designing a RCT is whether older adults can reasonably participate and adhere to the proposed intervention (in SPRINTT requiring regular PA sessions for a prolonged time). In case of a consistently low adherence, amendments to modify the intervention will be discussed within the SPRINTT Consortium together with IMI. Previous long-term trials of PA among elders suggest that adherence to physical training interventions usually is relatively high (60-85%) [17]. It is anticipated that the proposed study design (including frequent contacts with the study staff) will support the motivation of the participants at adhering to the study protocol.
- *Safety issues*. Standard “vigilance” activities will be implemented as part of the study protocol similarly to any other RCT in order to report and analyse possible adverse events occurring during the conduction of SPRINTT. The DSMB will regularly review safety data and propose the necessary steps for optimising the study protocol or decide its early termination.
- *Dysfunctions or quality issues in the conduction of the intervention*. A qualitatively high performance of the SPRINTT partners is critical and will be closely monitored. The poor performance of a centre will lead to its exclusion and possible redistribution of its activities to the other existing centres or back-up sites.
- *Inadequacy of ICT equipment*. The performance of the technical equipment has been tested and tuned during the SPRINTT preliminary phase. Regular monitoring of functioning will be conducted by the ICT partners for timely solving unforeseen issues and guarantee the proper conduction of activities. The implementation of a double system for recording actimetry data (Adamo watch and ActivPAL™) represents a built-in strategy to safeguard the successful collection of this important information.

13. Ethics

13.1. Institutional Review Board(s)/Independent Ethics Committee(s)

The study protocol, the "Participant information and consent form" document, the list of investigators document, the insurance documents, will be submitted to (an) IRB(s)/IEC(s) by the investigator(s) or the national coordinator(s) or the sponsor in accordance with local regulations.

The study will not start in a centre before written approval by corresponding IRB/IEC(s) has been obtained, the local regulatory requirements have been complied with, and the signature of the clinical study protocol of each contractual party involved has been obtained.

13.2. Study conduct

The study will be performed in accordance with the ethical principles stated in the Declaration of Helsinki 1964, as revised in Fortaleza, 2013 (see Appendix 1).

In any case, the participant must be informed that he/she is entitled to be informed about the outcome of the study by the research doctor.

The investigator or a person designated by him/her is to collect written consent from each participant before his/her participation in the study.

Prior to this, the investigator or his/her delegate must inform each participant of the objectives, benefits, risks and requirements imposed by the study.

The participant will be provided with an information and consent form in clear, simple language. He/she must be allowed ample time to inquire about details of the study and to decide whether or not to participate in the study.

Two original information and consent forms must be completed, dated and signed personally by the participant and by the person responsible for collecting the informed consent.

If the participant is unable to read, an impartial witness should be present during the entire informed consent discussion. The participant must give consent orally and, if capable of doing so, complete, sign and personally date the information and consent form. The witness must then complete, sign and date the form together with the person responsible for collecting the informed consent.

The participant will be given one signed original information and consent form; the second original will be kept by the investigator.

A copy of the information and consent form in the language(s) of the country is given in the “Participant information and consent form” document attached to the protocol.

The participant is still free to withdraw him or herself when he/she wants, at any time with destruction of his samples.

13.3. Modification of the information and consent form

Any change to the information and consent form constitutes an amendment to this document and must be submitted for approval to the IRB/IEC(s), and if applicable to the Competent Authorities.

A copy of the new version of the information and consent form in the language(s) of the country will be given in the amendment to the “Participant Information and consent form”.

Such amendments may only be implemented after written approval of the IRB/IEC has been obtained and compliance with the local regulatory requirements, with the exception of an amendment required to eliminate an immediate risk to the study participants.

Each participant affected by the amendment must complete, date and sign two originals of the new version of the information and consent form together with the person

who conducted the informed consent discussion. He/she will receive one signed original amendment to the information and consent form.

14. Study sites

14.1. Procedures for selection of study sites

The pre-selection of the sites which will carry out the clinical trial was operated by the SPRINTT Consortium under the coordination of the Catholic University of Sacred Heart (Rome, Italy). A pre-feasibility questionnaire was sent to clinical and research sites located across Europe that had expressed their interest in the project at the time of preparation of the "Expression of Interest".

The following criteria were considered for pre-selection:

- Interest in participating to the project
- Previous experience with clinical studies involving vulnerable older people
- Access to the target population
- Adequate estimated recruitment capacity
- Presence of qualified personnel for conducting clinical studies involving multidimensional assessments and for implementing the multi-component intervention of SPRINTT (minimum requirements: physicians, nutritionist, physical exercise trainer, DXA technician, nurses, lab technician, study coordinator)
- Availability of equipment and instrumentations for participant assessment and implementation of the intervention (minimum requirements: 25-metre-long corridor for the 400-m walk test, exam rooms, DXA, handgrip dynamometer, refrigerated centrifuge, -20° freezer, on-site gym or adequate space to accommodate the physical exercise intervention, on-site certified laboratory for standard blood work, availability of computers and internet access).

Only those study sites fulfilling all the above-listed criteria were pre-selected. Based on the pre-feasibility survey, 14 sites were chosen. The sites are located in 11 countries, organised in 7 regional areas (see table below). The pre-selection procedure allowed reducing the number of potential study sites, therefore decreasing variability and operational costs. At the same time, attention was paid in assuring that the European geography would be adequately represented within the SPRINTT project. Each pre-selected study site was certified during an on-site visit by an *ad hoc* established "standardisation team" in the presence of the Local Coordinator.

SPRINTT study sites.

Regional area	Country	Site name	Local coordinator
Italy (Responsible: Francesco Landi)	Italy	Catholic University of the Sacred Heart School of Medicine (Rome; RCT Coordinating Site)	Francesco Landi
	Italy	IRCCS-INRCA (Ancona)	Antonio Cherubini
France (Responsible: Bruno Vellas)	France	CHU Toulouse (Toulouse)	Matteo Cesari
	France	CHU Limoges (Limoges)	Thierry Dantoine
Spain (Responsible: Leocadio Rodriguez Mañas)	Spain	Getafe University Hospital (Madrid)	Leocadio Rodriguez Mañas
	Spain	Hospital Universitario Ramón y Cajal (Madrid)	Alfonso J Cruz Jentoft

Germany & Austria (Responsible: Cornel Sieber)	Germany	Friedrich-Alexander Universität Erlangen-Nürnberg (Nurnberg)	Cornel Sieber
	Germany	University of Hamburg (Hamburg)	Wolfgang von Renteln-Kruse
United Kingdom (Responsible: Alan Sinclair)	United Kingdom	Diabetes Frail, Medici Medical Practice (Luton)	Alan Sinclair
	United Kingdom	Heart Of England NHS Foundation Trust (Birmingham)	Srikanth Bellary
Eastern Europe (Responsible: Eva Topinkova)	Czech Republic	Charles University (Prague)	Eva Topinkova
	Poland	Jagiellonian University Medical College (Krakow)	Anna Skalska
Nordic Countries & The Netherlands (Responsible: Timo Strandberg)	Finland	University of Helsinki	Timo Strandberg
	The Netherlands	Maastricht University Medical Center (Maastricht)	Jos Schols

14.2. Brief description of the SPRINTT study site

14.2.1. Catholic University of the Sacred Heart - Centre for Geriatric Medicine

The Centro di Medicina dell'Invecchiamento (Centre for Geriatric Medicine, CeMI) was inaugurated in 1998 and since then it has been considered a unique environment in which care, research and education form an inspiring and stimulating continuum. Under the direction of Prof. Roberto Bernabei, the CeMI has operated as a natural laboratory for the Italian Ministry of Health and for the Ministry of Welfare, for its vision of an integrated approach to the problems of a rapidly aging population. The Centre occupies a seven-story facility, hosting the Department of Geriatrics, Neurosciences and Orthopaedics at the Catholic University of the Sacred Heart in Rome (Italy). The building hosts a 24-bed acute-care ward, an intensive rehabilitation unit comprising 30 beds, an orthogeriatric unit comprising 10 beds, a day-hospital with 20 beds, and an outpatient clinic including several specialised centres. The CeMI occupies an entire building, which is physically separated from the main hospital, but it is integrated and networked with the entire campus. The CeMI is a fully computerised facility controlled by a dedicated system manager who runs a state-of-the art server allowing the necessary technological sophistication. In addition to the facilities on campus, the CeMI's staff operates two large, hospital-affiliated, nursing homes, and a clinical centre. The CeMI has a parking lot in front of the building and a direct entrance to facilitate access of research participants, a reception and waiting area and five exam and interview rooms. Visit rooms are also suitable for phlebotomy. The CeMI has its own DXA scanner (Hologic) located in a dedicated room. In the basement, the facility hosts a fully equipped gym and several rooms for physical exercise. Outside the gym and one floor up, four corridors are available for 400-metre walk testing. At the ground level and the first floor, there are two rooms equipped for multimedia presentations where workshops and seminars will be held for the HALE group. The first floor also hosts office areas, equipped with computers and locked cabinets for securely storing trial documents. Located in the CeMI is the Biogerontology Laboratory. The lab is equipped with state-of-the-art instrumentation for proteomic and genomic analyses, besides standard equipment for sample processing and storage. The lab and its adjacent office

area are also equipped with computers connected to the department networks. Biospecimens collected during the trial will be processed and temporarily stored at -80°C within the lab. Back-up -80°C and -20°C freezers are also available in the facility.

The CeMI hosts a MacPro Server with 4 hard drives and 7 TByte of memory mass storage to store information collected in the MY CONCeRT database. A computer scientist and a statistician of the CEMI will administer and maintain the database.

The number of participants to be enrolled at the CeMI is 107. Specific recruitment strategies and tools will be used to reach the target enrolment. Brochures and flyers illustrating the study purposes and interventions will be prepared to assist in publicising the study during the 12-month recruitment period. The main recruitment sources will include the CeMI outpatient clinic and day-hospital as well as registries of subjects who have participated in other research studies. Spouses and friends of inpatients will also be informed about the study and invited to participate. Colleagues within the Teaching Hospital will be informed of the study through direct calls, departmental/practice meetings, word of mouth, brochures, and recruitment flyers. A press release will be issued to announce the study to initiate recruitment. Advertisements will be placed in local area newspapers. Information about the study will also be provided through the participation of SPRINTT investigators to local and national TV as well as radio programmes.

15. References

1. Cesari M, Vellas B, Gambassi G. The stress of aging. *Exp Gerontol*. 2013;48(4):451-456.
2. Rodríguez-Mañas L, Féart C, Mann G et al. Searching for an Operational Definition of Frailty: A Delphi Method Based Consensus Statement. The Frailty Operative Definition-Consensus Conference Project. *J Gerontol A Biol Sci Med Sci*. 2012;68(1):62-67.
3. Fried LP, Walston J. Frailty and failure to thrive. In: Hazzard WR, Blass JP, Ettinger WH, Halter JB, Ouslander JG, eds. *Principles of Geriatric Medicine and Gerontology*. New York: McGraw-Hill; 1998:1387-1402.
4. Fried LP, Tangen CM, Walston J et al. Frailty in older adults: evidence for a phenotype. *J Gerontol A Biol Sci Med Sci*. 2001;56(3):M146-M156.
5. Rockwood K, Song X, MacKnight C et al. A global clinical measure of fitness and frailty in elderly people. *CMAJ*. 2005;173(5):489-495.
6. Ensrud K, Ewing SK, Taylor BC et al. Comparison of 2 frailty indexes for prediction of falls, disability, fractures, and death in older women. *Arch Intern Med*. 2008;168(4):382-389.
7. Gobbens RJ, van Assen MA, Luijkx KG, Wijnen-Sponselee MT, Schols JM. The Tilburg Frailty Indicator: psychometric properties. *J Am Med Dir Assoc*. 2010;11(5):344-355.
8. Buchman AS, Boyle PA, Wilson RS, Tang Y, Bennett DA. Frailty is associated with incident Alzheimer's disease and cognitive decline in the elderly. *Psychosom Med*. 2007;69(5):483-489.
9. Morley JE, Malmstrom TK, Miller DK. A Simple Frailty Questionnaire (FRAIL) Predicts Outcomes in Middle Aged African Americans. *J Nutr Health Aging*. 2012;16(7):601-608.
10. Vellas B, Balardy L, Gillette-Guyonnet S et al. Looking for Frailty in Community-Dwelling Older Persons: The Gerontopole Frailty Screening Tool (GFST). *J Nutr Health Aging*. 2013;17(7):629-631.
11. Cesari M, Demougeot L, Boccalon H et al. A Self-Reported Screening Tool for Detecting Community-Dwelling Older Persons with Frailty Syndrome in the Absence of Mobility Disability: The FiND Questionnaire. *PLoS One*. 2014;9(7):e101745.
12. Theou O, Brothers TD, Mitnitski A, Rockwood K. Operationalization of frailty using eight commonly used scales and comparison of their ability to predict all-cause mortality. *J Am Geriatr Soc*. 2013;61(9):1537-1551.
13. Hoogendijk EO, van der Horst HE, Deeg DJ et al. The identification of frail older adults in primary care: comparing the accuracy of five simple instruments. *Age Ageing*. 2013;42(2):262-265.
14. Morley JE, Vellas B, Abellan van Kan G et al. Frailty consensus: a call to action. *J Am Med Dir Assoc*. 2013;14(6):392-397.
15. Cruz-Jentoft AJ, Baeyens JP, Bauer JM et al. Sarcopenia: European consensus on definition and diagnosis: Report of the European Working Group on Sarcopenia in Older People. *Age Ageing*. 2010;39(4):412-423.
16. Cesari M, Landi F, Vellas B, Bernabei R, Marzetti E. Sarcopenia and physical frailty: two sides of the same coin. *Front Aging Neurosci*. 2014;6:192.
17. Pahor M, Guralnik JM, Ambrosius WT et al. Effect of Structured Physical Activity on Prevention of Major Mobility Disability in Older Adults: The LIFE Study Randomized Clinical Trial. *JAMA*. 2014;311(23):2387-2396.
18. *Fit for Frailty - consensus best practice guidance for the care of older people living in community and outpatient settings - a report from the British Geriatrics Society.*

British Geriatrics Society; 2014.
19. Fried TR, Bradley EH, Williams CS, Tinetti ME. Functional disability and health care expenditures for older persons. *Arch Intern Med*. 2001;161(21):2602-2607.
20. Clegg A, Young J, Iliffe S, Rikkert MO, Rockwood K. Frailty in elderly people. *Lancet*. 2013;381:752-762.
21. Ferrucci L, Cavazzini C, Corsi A et al. Biomarkers of frailty in older persons. *J Endocrinol Invest*. 2002;25(10 Suppl):10-15.
22. Abellan van Kan G, Rolland YM, Morley JE, Vellas B. Frailty: toward a clinical definition. *J Am Med Dir Assoc*. 2008;9(2):71-72.
23. Santos-Eggimann B, Cuénoud P, Spagnoli J, Junod J. Prevalence of frailty in middle-aged and older community-dwelling Europeans living in 10 countries. *J Gerontol A Biol Sci Med Sci*. 2009;64(6):675-681.
24. Xue QL. The frailty syndrome: definition and natural history. *Clin Geriatr Med*. 2011;27(1):1-15.
25. Gill TM, Gahbauer EA, Allore HG, Han L. Transitions between frailty states among community-living older persons. *Arch Intern Med*. 2006;166(4):418-423.
26. Cesari M. The multidimentionality of frailty: many faces of one single dice. *J Nutr Health Aging*. 2011;15(8):663-664.
27. Rockwood K, Bergman H. FRAILTY: A Report from the 3(rd) Joint Workshop of IAGG/WHO/SFGG, Athens, January 2012. *Can Geriatr J*. 2012;15(2):31-36.
28. Walston J, McBurnie MA, Newman AB et al. Frailty and activation of the inflammation and coagulation systems with and without clinical comorbidities: results from the Cardiovascular Health Study. *Arch Intern Med*. 2002;162(20):2333-2341.
29. Marzetti E, Calvani R, Bernabei R, Leeuwenburgh C. Apoptosis in skeletal myocytes: a potential target for interventions against sarcopenia and physical frailty - a mini-review. *Gerontology*. 2012;58(2):99-106.
30. Fielding RA, Rejeski WJ, Blair S et al. The Lifestyle Interventions and Independence for Elders Study: Design and Methods. *J Gerontol A Biol Sci Med Sci*. 2011;66(11):1226-1237.
31. Calvani R, Miccheli A, Landi F et al. Current nutritional recommendations and novel dietary strategies to manage sarcopenia. *J Frailty Aging*. 2013;2 (1):38-53.
32. Studenski SA, Peters KW, Alley DE et al. The FNIH Sarcopenia Project: Rationale, Study Description, Conference Recommendations, and Final Estimates. *J Gerontol A Biol Sci Med Sci*. 2014;69(5):547-558.
33. Guralnik JM, Ferrucci L, Simonsick EM, Salive ME, Wallace RB. Lower-extremity function in persons over the age of 70 years as a predictor of subsequent disability. *N Engl J Med*. 1995;332(9):556-561.
34. Studenski S, Perera S, Wallace D et al. Physical performance measures in the clinical setting. *J Am Geriatr Soc*. 2003;51(3):314-322.
35. Guralnik JM, Ferrucci L, Pieper CF et al. Lower extremity function and subsequent disability: consistency across studies, predictive models, and value of gait speed alone compared with the short physical performance battery. *J Gerontol A Biol Sci Med Sci*. 2000;55(4):M221-M231.
36. Abizanda P, Romero L, Sanchez-Jurado PM, Atienzar-Núñez P, Esquinas-Requena JL, Garcia-Nogueras I. Association between functional assessment instruments and frailty in older adults: the FRADEA study. *J Frailty Aging*. 2012;1(4):162-168.
37. Newman AB, Simonsick EM, Naydeck BL et al. Association of long-distance corridor walk performance with mortality, cardiovascular disease, mobility limitation, and disability. *JAMA*. 2006;295(17):2018-2026.
38. Pahor M, Blair SN, Espeland M et al. Effects of a physical activity intervention on measures of physical performance: Results of the lifestyle interventions and

independence for Elders Pilot (LIFE-P) study. *J Gerontol A Biol Sci Med Sci*. 2006;61(11):1157-1165.
39. Cesari M, Vellas B, Hsu FC et al. A Physical Activity Intervention to Treat the Frailty Syndrome in Older Persons-Results From the LIFE-P Study. *J Gerontol A Biol Sci Med Sci*. 2015;70(2):216-222.
40. King AC, Rejeski WJ, Buchner DM. Physical activity interventions targeting older adults. A critical review and recommendations. *Am J Prev Med*. 1998;15(4):316-333.
41. Borg G. Perceived exertion as an indicator of somatic stress. *Scand J Rehabil Med*. 1970;2(2):92-98.
42. Ettinger WHJ, Burns R, Messier SP et al. A randomized trial comparing aerobic exercise and resistance exercise with a health education program in older adults with knee osteoarthritis. The Fitness Arthritis and Seniors Trial (FAST). *JAMA*. 1997;277(1):25-31.
43. King AC, Friedman R, Marcus B et al. Harnessing motivational forces in the promotion of physical activity: the Community Health Advice by Telephone (CHAT) project. *Health Educ Res*. 2002;17(5):627-636.
44. King AC, Haskell WL, Young DR, Oka RK, Stefanick ML. Long-term effects of varying intensities and formats of physical activity on participation rates, fitness, and lipoproteins in men and women aged 50 to 65 years. *Circulation*. 1995;91(10):2596-2604.
45. King AC, Baumann K, O'Sullivan P, Wilcox S, Castro C. Effects of moderate-intensity exercise on physiological, behavioral, and emotional responses to family caregiving: a randomized controlled trial. *J Gerontol A Biol Sci Med Sci*. 2002;57(1):M26-M36.
46. Abellan van Kan G, Rolland Y, Houles M, Gillette-Guyonnet S, Soto M, Vellas B. The assessment of frailty in older adults. *Clin Geriatr Med*. 2010;26(2):275-286.
47. Kelaiditi E, Guyonnet S, Cesari M. Is nutrition important to postpone frailty? *Curr Opin Clin Nutr Metab Care*. 2015;18(1):37-42.
48. Correia MI, Hegazi RA, Higashiguchi T et al. Evidence-based recommendations for addressing malnutrition in health care: an updated strategy from the feedM.E. Global Study Group. *J Am Med Dir Assoc*. 2014;15(8):544-550.
49. Bauer J, Biolo G, Cederholm T et al. Evidence-Based Recommendations for Optimal Dietary Protein Intake in Older People: A Position Paper From the PROT-AGE Study Group. *J Am Med Dir Assoc*. 2013;14(8):542-559.
50. Recommendations Abstracted from the American Geriatrics Society Consensus Statement on Vitamin D for Prevention of Falls and Their Consequences. *J Am Geriatr Soc*. 2014;62 (1):147-152.
51. Rolland YM, Cesari M, Miller ME, Penninx BW, Atkinson HH, Pahor M. Reliability of the 400-m usual-pace walk test as an assessment of mobility limitation in older adults. *J Am Geriatr Soc*. 2004;52(6):972-976.
52. Rantanen T, Guralnik JM, Foley D et al. Midlife hand grip strength as a predictor of old age disability. *JAMA*. 1999;281(6):558-560.
53. Rantanen T, Harris T, Leveille SG et al. Muscle strength and body mass index as long-term predictors of mortality in initially healthy men. *J Gerontol A Biol Sci Med Sci*. 2000;55(3):M168-M173.
54. Rejeski WJ, Ip EH, Marsh AP, Miller ME, Farmer DF. Measuring disability in older adults: the International Classification System of Functioning, Disability and Health (ICF) framework. *Geriatr Gerontol Int*. 2008;8(1):48-54.
55. Rejeski WJ, Ip EH, Marsh AP, Zhang Q, Miller ME. Obesity influences transitional states of disability in older adults with knee pain. *Arch Phys Med Rehabil*. 2008;89(11):2102-2107.
56. Katz S, Ford AB, Moskowitz RW, Jackson BA, Jaffe MW. Studies of illness in the

aged. The index of ADL: a standardized measure of biological and psychosocial function. *JAMA*. 1963;185:914-919.
57. Patel KV, Coppin AK, Manini TM et al. Midlife physical activity and mobility in older age: The InCHIANTI study. *Am J Prev Med*. 2006;31(3):217-224.
58. Landi F, Cesari M, Onder G, Lattanzio F, Gravina EM, Bernabei R. Physical activity and mortality in frail, community-living elderly patients. *J Gerontol A Biol Sci Med Sci*. 2004;59(8):833-837.
59. Psaty BM, Lee M, Savage PJ, Rutan GH, German PS, Lyles M. Assessing the use of medications in the elderly: methods and initial experience in the Cardiovascular Health Study. The Cardiovascular Health Study Collaborative Research Group. *J Clin Epidemiol*. 1992;45(6):683-692.
60. Folstein MF, Folstein SE, McHugh PR. “Mini-mental state”. A practical method for grading the cognitive state of patients for the clinician. *J Psychiatr Res*. 1975;12(3):189-198.
61. Lezak MD. Orientation and attention. In: Lezak MD, ed. *Neuropsychological assessment*. New York, NY: Oxford University Press; 1995:335-384.
62. Dolan P. Modeling valuations for EuroQol health states. *Med Care*. 1997;35(11):1095-1108.
63. Prieto L, Sacristán JA. What is the value of social values? The uselessness of assessing health-related quality of life through preference measures. *BMC Med Res Methodol*. 2004;4:10.
64. Rabin R, de Charro F. EQ-5D: a measure of health status from the EuroQol Group. *Ann Med*. 2001;33(5):337-343.
65. Radloff LS. The CES-D scale: a self-report depression scale for research in the general population. *Appl Psychol Meas*. 1977;1:385-401.
66. Kohout FJ, Berkman LF, Evans DA, Cornoni-Huntley J. Two shorter forms of the CES-D (Center for Epidemiological Studies Depression) depression symptoms index. *J Aging Health*. 1993;5(2):179-193.
67. Guigoz Y. The Mini Nutritional Assessment (MNA) review of the literature--What does it tell us? *J Nutr Health Aging*. 2006;10(6):466-85; discussion 485.
68. Parmelee PA, Thuras PD, Katz IR, Lawton MP. Validation of the Cumulative Illness Rating Scale in a geriatric residential population. *J Am Geriatr Soc*. 1995;43(2):130-137.
69. Malmstrom TK, Morley JE. SARC-F: a simple questionnaire to rapidly diagnose sarcopenia. *J Am Med Dir Assoc*. 2013;14(8):531-532.
70. Wimo A, Jonsson L, Zbrozek A. The Resource Utilization in Dementia (RUD) instrument is valid for assessing informal care time in community-living patients with dementia. *J Nutr Health Aging*. 2010;14(8):685-690.
71. Salamone LM, Fuerst T, Visser M et al. Measurement of fat mass using DEXA: a validation study in elderly adults. *J Appl Physiol*. 2000;89(1):345-352.
72. Visser M, Fuerst T, Lang T, Salamone L, Harris TB. Validity of fan-beam dual-energy X-ray absorptiometry for measuring fat-free mass and leg muscle mass. Health, Aging, and Body Composition Study--Dual-Energy X-ray Absorptiometry and Body Composition Working Group. *J Appl Physiol*. 1999;87(4):1513-1520.
73. Gill TM, DiPietro L, Krumholz HM. Role of exercise stress testing and safety monitoring for older persons starting an exercise program. *JAMA*. 2000;284(3):342-349.
74. Chodzko-Zajko WJ, Proctor DN, Fiatarone Singh MA et al. American College of Sports Medicine position stand. Exercise and physical activity for older adults. *Med Sci Sports Exerc*. 2009;41(7):1510-1530.
75. Fletcher GF, Balady GJ, Amsterdam EA et al. Exercise standards for testing and

training: a statement for healthcare professionals from the American Heart Association. *Circulation*. 2001;104(14):1694-1740.
76. Hollenberg M, Ngo LH, Turner D, Tager IB. Treadmill exercise testing in an epidemiologic study of elderly subjects. *J Gerontol A Biol Sci Med Sci*. 1998;53(4):B259-B267.
77. Furberg CD, Manolio TA, Psaty BM et al. Major electrocardiographic abnormalities in persons aged 65 years and older (the Cardiovascular Health Study). Cardiovascular Health Study Collaborative Research Group. *Am J Cardiol*. 1992;69(16):1329-1335.
78. Hakim AA, Curb JD, Petrovitch H et al. Effects of walking on coronary heart disease in elderly men: the Honolulu Heart Program. *Circulation*. 1999;100(1):9-13.
79. Hakim AA, Petrovitch H, Burchfiel CM et al. Effects of walking on mortality among nonsmoking retired men. *N Engl J Med*. 1998;338(2):94-99.
80. American College of Sports Medicine Position Stand. Exercise and physical activity for older adults. *Med Sci Sports Exerc*. 1998;30(6):992-1008.

16. Appendices

Appendix 1: World Medical Association Declaration of Helsinki

WORLD MEDICAL ASSOCIATION DECLARATION OF HELSINKI

Ethical Principles for Medical Research Involving Human Subjects

Adopted by the 18th WMA General Assembly, Helsinki, Finland, June 1964, and amended by the:
29th WMA General Assembly, Tokyo, Japan, October 1975
35th WMA General Assembly, Venice, Italy, October 1983
41st WMA General Assembly, Hong Kong, September 1989
48th WMA General Assembly, Somerset West, Republic of South Africa, October 1996
52nd WMA General Assembly, Edinburgh, Scotland, October 2000
53th WMA General Assembly, Washington DC, USA, 2002 (Note of Clarification added)
55th WMA General Assembly, Tokyo, Japan, 2004 (Note of Clarification added)
59th WMA General Assembly, Seoul, Republic of Korea, October 2008
64th WMA General Assembly, Fortaleza, Brazil, October 2013

Preamble

1. The World Medical Association (WMA) has developed the Declaration of Helsinki as a statement of ethical principles for medical research involving human subjects**,** including research on identifiable human material and data.

   The Declaration is intended to be read as a whole and each of its constituent paragraphs should be applied with consideration of all other relevant paragraphs.

2. Consistent with the mandate of the WMA, the Declaration is addressed primarily to physicians. The WMA encourages others who are involved in medical research involving human subjects to adopt these principles

General Principles

3. The Declaration of Geneva of the WMA binds the physician with the words, “The health of my patient will be my first consideration,” and the International Code of Medical Ethics declares that, “A physician shall act in the patient's best interest when providing medical care.”

4. It is the duty of the physician to promote and safeguard the health, well-being and rights of patients, including those who are involved in medical research. The physician's knowledge and conscience are dedicated to the fulfilment of this duty.

5. Medical progress is based on research that ultimately must include studies involving human subjects.

6. The primary purpose of medical research involving human subjects is to understand the causes, development and effects of diseases and improve preventive, diagnostic and therapeutic interventions (methods, procedures and treatments). Even the best

proven interventions must be evaluated continually through research for their safety, effectiveness, efficiency, accessibility and quality.

7. Medical research is subject to ethical standards that promote and ensure respect for all human subjects and protect their health and rights.

8. While the primary purpose of medical research is to generate new knowledge, this goal can never take precedence over the rights and interests of individual research subjects.

9. It is the duty of physicians who are involved in medical research to protect the life, health, dignity, integrity, right to self-determination, privacy, and confidentiality of personal information of research subjects. The responsibility for the protection of research subjects must always rest with the physician or other health care professionals and never with the research subjects, even though they have given consent.

10. Physicians must consider the ethical, legal and regulatory norms and standards for research involving human subjects in their own countries as well as applicable international norms and standards. No national or international ethical, legal or regulatory requirement should reduce or eliminate any of the protections for research subjects set forth in this Declaration.

11. Medical research should be conducted in a manner that minimises possible harm to the environment.

12. Medical research involving human subjects must be conducted only by individuals with the appropriate ethics and scientific education, training and qualifications. Research on patients or healthy volunteers requires the supervision of a competent and appropriately qualified physician or other health care professional.

13. Groups that are underrepresented in medical research should be provided appropriate access to participation in research.

14. Physicians who combine medical research with medical care should involve their patients in research only to the extent that this is justified by its potential preventive, diagnostic or therapeutic value and if the physician has good reason to believe that participation in the research study will not adversely affect the health of the patients who serve as research subjects.

15. Appropriate compensation and treatment for subjects who are harmed as a result of participating in research must be ensured.

Risk, Burdens and Benefits

16. In medical practice and in medical research, most interventions involve risks and burdens.

    Medical research involving human subjects may only be conducted if the importance of the objective outweighs the risks and burdens to the research subjects.

17. All medical research involving human subjects must be preceded by careful assessment of predictable risks and burdens to the individuals and groups involved in the research in comparison with foreseeable benefits to them and to other individuals or groups affected by the condition under investigation.

    Measures to minimise the risks must be implemented. The risks must be continuously monitored, assessed and documented by the researcher.

18. Physicians may not be involved in a research study involving human subjects unless they are confident that the risks have been adequately assessed and can be satisfactorily managed.

    When the risks are found to outweigh the potential benefits or when there is conclusive proof of definitive outcomes, physicians must assess whether to continue, modify or immediately stop the study.

Vulnerable Groups and Individuals

19. Some groups and individuals are particularly vulnerable and may have an increased likelihood of being wronged or of incurring additional harm.

    All vulnerable groups and individuals should receive specifically considered protection.

20. Medical research with a vulnerable group is only justified if the research is responsive to the health needs or priorities of this group and the research cannot be carried out in a non-vulnerable group. In addition, this group should stand to benefit from the knowledge, practices or interventions that result from the research.

Scientific Requirements and Research Protocols

21. Medical research involving human subjects must conform to generally accepted scientific principles, be based on a thorough knowledge of the scientific literature, other relevant sources of information, and adequate laboratory and, as appropriate, animal experimentation. The welfare of animals used for research must be respected.

22. The design and performance of each research study involving human subjects must be clearly described and justified in a research protocol.

    The protocol should contain a statement of the ethical considerations involved and should indicate how the principles in this Declaration have been addressed. The protocol should include information regarding funding, sponsors, institutional affiliations, potential conflicts of interest, incentives for subjects and information

regarding provisions for treating and/or compensating subjects who are harmed as a consequence of participation in the research study.

In clinical trials, the protocol must also describe appropriate arrangements for post-trial provisions.

Research Ethics Committees

23. The research protocol must be submitted for consideration, comment, guidance and approval to the concerned research ethics committee before the study begins. This committee must be transparent in its functioning, must be independent of the researcher, the sponsor and any other undue influence and must be duly qualified. It must take into consideration the laws and regulations of the country or countries in which the research is to be performed as well as applicable international norms and standards but these must not be allowed to reduce or eliminate any of the protections for research subjects set forth in this Declaration.

    The committee must have the right to monitor on-going studies. The researcher must provide monitoring information to the committee, especially information about any serious adverse events. No amendment to the protocol may be made without consideration and approval by the committee. After the end of the study, the researchers must submit a final report to the committee containing a summary of the study's findings and conclusions.

Privacy and Confidentiality

24. Every precaution must be taken to protect the privacy of research subjects and the confidentiality of their personal information.

Informed Consent

25. Participation by individuals capable of giving informed consent as subjects in medical research must be voluntary. Although it may be appropriate to consult family members or community leaders, no individual capable of giving informed consent may be enrolled in a research study unless he or she freely agrees.

26. In medical research involving human subjects capable of giving informed consent, each potential subject must be adequately informed of the aims, methods, sources of funding, any possible conflicts of interest, institutional affiliations of the researcher, the anticipated benefits and potential risks of the study and the discomfort it may entail, post-study provisions and any other relevant aspects of the study. The potential subject must be informed of the right to refuse to participate in the study or to withdraw consent to participate at any time without reprisal. Special attention should be given to the specific information needs of individual potential subjects as well as to the methods used to deliver the information.

    After ensuring that the potential subject has understood the information, the physician or another appropriately qualified individual must then seek the potential

subject's freely-given informed consent, preferably in writing. If the consent cannot be expressed in writing, the non-written consent must be formally documented and witnessed.

All medical research subjects should be given the option of being informed about the general outcome and results of the study.

27. When seeking informed consent for participation in a research study the physician must be particularly cautious if the potential subject is in a dependent relationship with the physician or may consent under duress. In such situations the informed consent must be sought by an appropriately qualified individual who is completely independent of this relationship.

28. For a potential research subject who is incapable of giving informed consent, the physician must seek informed consent from the legally authorised representative. These individuals must not be included in a research study that has no likelihood of benefit for them unless it is intended to promote the health of the group represented by the potential subject, the research cannot instead be performed with persons capable of providing informed consent, and the research entails only minimal risk and minimal burden.

29. When a potential research subject who is deemed incapable of giving informed consent is able to give assent to decisions about participation in research, the physician must seek that assent in addition to the consent of the legally authorised representative. The potential subject's dissent should be respected.

30. Research involving subjects who are physically or mentally incapable of giving consent, for example, unconscious patients, may be done only if the physical or mental condition that prevents giving informed consent is a necessary characteristic of the research group. In such circumstances the physician must seek informed consent from the legally authorised representative. If no such representative is available and if the research cannot be delayed, the study may proceed without informed consent provided that the specific reasons for involving subjects with a condition that renders them unable to give informed consent have been stated in the research protocol and the study has been approved by a research ethics committee. Consent to remain in the research must be obtained as soon as possible from the subject or a legally authorised representative.

31. The physician must fully inform the patient which aspects of their care are related to the research. The refusal of a patient to participate in a study or the patient's decision to withdraw from the study must never adversely affect the patient-physician relationship.

32. For medical research using identifiable human material or data, such as research on material or data contained in biobanks or similar repositories, physicians must seek informed consent for its collection, storage and/or reuse. There may be exceptional situations where consent would be impossible or impracticable to obtain for such research. In such situations the research may be done only after consideration and approval of a research ethics committee.

Use of Placebo

33. The benefits, risks, burdens and effectiveness of a new intervention must be tested against those of the best proven intervention(s), except in the following circumstances:

    Where no proven intervention exists, the use of placebo, or no intervention, is acceptable; or

    Where for compelling and scientifically sound methodological reasons the use of any intervention less effective than the best proven one, the use of placebo, or no intervention is necessary to determine the efficacy or safety of an intervention

    and the patients who receive any intervention less effective than the best proven one, placebo, or no intervention will not be subject to additional risks of serious or irreversible harm as a result of not receiving the best proven intervention.

    Extreme care must be taken to avoid abuse of this option.

Post-Trial Provisions

34. In advance of a clinical trial, sponsors, researchers and host country governments should make provisions for post-trial access for all participants who still need an intervention identified as beneficial in the trial. This information must also be disclosed to participants during the informed consent process.

Research Registration and Publication and Dissemination of Results

35. Every research study involving human subjects must be registered in a publicly accessible database before recruitment of the first subject.

36. Researchers, authors, sponsors, editors and publishers all have ethical obligations with regard to the publication and dissemination of the results of research. Researchers have a duty to make publicly available the results of their research on human subjects and are accountable for the completeness and accuracy of their reports. All parties should adhere to accepted guidelines for ethical reporting. Negative and inconclusive as well as positive results must be published or otherwise made publicly available. Sources of funding, institutional affiliations and conflicts of interest must be declared in the publication. Reports of research not in accordance with the principles of this Declaration should not be accepted for publication.

Unproven Intervention in Clinical Practice

In the treatment of an individual patient, where proven interventions do not exist or other known interventions have been ineffective, the physician, after seeking expert advice, with informed consent from the patient or a legally authorised representative, may use an unproven intervention if in the physician's judgment it offers hope of saving life, re-establishing health or alleviating suffering. This intervention should subsequently be made

the object of research, designed to evaluate its safety and efficacy. In all cases, new information must be recorded and, where appropriate, made publicly available.

STATISTICAL ANALYSIS PLAN

Sarcopenia and Physical fRailty IN older people: multi-componenT Treatment strategies

SPRINTT

A randomised clinical trial comparing the efficacy of a multicomponent intervention (based on long-term structured physical activity, nutritional counselling and information & communication technology intervention) versus a Healthy Aging Lifestyle Education (HALE) programme for preventing incident mobility disability in community-dwelling older persons with physical frailty and sarcopenia

---

Total number of pages: 46

TABLE OF CONTENTS

STATISTICAL ANALYSIS PLAN 1

TABLE OF CONTENTS 2

LIST OF ABBREVIATIONS AND DEFINITION OF TERMS 4

1 OVERVIEW AND INVESTIGATIONAL PLAN 5

1.1 STUDY DESIGN AND RANDOMIZATION 5

1.2 OBJECTIVES 5
1.2.1 Primary objectives 5
1.2.2 Secondary objectives 6

1.3 DETERMINATION OF SAMPLE SIZE 6

1.4 STUDY PLAN 7

1.5 MODIFICATIONS TO THE STATISTICAL SECTION OF THE PROTOCOL 8

1.6 STATISTICAL MODIFICATIONS MADE IN THE STATISTICAL ANALYSIS PLAN 8

2 STATISTICAL AND ANALYTICAL PROCEDURES 9

2.1 ANALYSIS ENDPOINTS 9
2.1.1 Demographic and baseline characteristics 9
2.1.2 Prior or concomitant medications 11
2.1.3 Efficacy endpoints 11
2.1.3.1 Primary efficacy endpoint(s) 12
2.1.3.2 Secondary efficacy endpoint(s) 14
2.1.4 Safety endpoints 17
2.1.4.1 Adverse events variables 17
2.1.4.2 Falls 18
2.1.4.3 Deaths 19
2.1.4.4 Laboratory safety variables 19
2.1.4.5 Vital signs variables 19
2.1.5 Quality-of-life endpoints 20
2.1.6 Health economic endpoints 20
2.1.7 Biomarker endpoints 20

2.2 DISPOSITION OF PARTICIPANTS 20

2.3 ANALYSIS POPULATIONS 21

2.4 STATISTICAL METHODS 22
2.4.1 Demographics and baseline characteristics 22
2.4.2 Prior or concomitant medications....22
2.4.3 Intervention attendance and compliance 23
2.4.3.1 Intervention attendance 23
2.4.3.2 Compliance 24
2.4.4 Analyses of efficacy endpoints....24
2.4.4.1 Analysis of primary efficacy endpoint(s) 24
2.4.4.2 Analyses of secondary efficacy endpoints....26
2.4.4.3 Multiplicity issues 27
2.4.5 Analyses of safety data....27
2.4.5.1 Analyses of adverse events 28
2.4.5.2 Falls....31
2.4.5.3 Deaths....31
2.4.5.4 Analyses of laboratory variables....31
2.4.5.5 Analyses of vital sign variables....32
2.4.6 Analyses of pharmacokinetic and pharmacodynamic variables 32
2.4.7 Analyses of quality of life variables....33

2.5 DATA HANDLING CONVENTIONS 33
2.5.1 General conventions 33
2.5.2 Data handling conventions for secondary efficacy variables....35
2.5.3 Missing data....35
2.5.4 Windows for time points....36
2.5.5 Unscheduled visits 36
2.5.6 Pooling of centers for statistical analyses....37

3 INTERIM ANALYSIS 38

4 DATABASE LOCK 39

5 SOFTWARE DOCUMENTATION 40

6 REFERENCES....41

7 LIST OF APPENDICES....42

APPENDIX A STUDY FLOWCHART....43

APPENDIX B VITAL SIGNS ABNORMALITIES AND ALERT....45

APPENDIX C BLOOD TEST ABNORMALITIES....46

LIST OF ABBREVIATIONS AND DEFINITION OF TERMS

AE:	Adverse event
AESI:	Adverse event of special interest
BMI:	Body Mass Index
BMI:	Body mass index
CES-D:	Center for Epidemiological Studies-Depression, Center for Epidemiological Studies-Depression
CI:	Confidence interval
COPD:	Chronic obstructive pulmonary disease
EAE:	Emergent adverse event
HALE:	Healthy Aging Lifestyle Education
HLGT:	High level group term
HLT:	High level term
ICT:	Information and Communication Technology
KM:	Kaplan Meier
LLT:	Lower level term
MCI:	MultiComponent Intervention
MedDRA:	Medical Dictionary for Regulatory Activities
MMRM:	Mixed-effect model with repeated measures
MMSE:	Mini Mental State Examination
MNA-SF:	Mini Nutritional Assessment-Short Form
PA:	Physical Activity
PF&S:	Physical frailty and sarcopenia
PT:	Preferred term
RCT:	Randomised controlled trial
SOC:	System organ class
SPPB:	Short Physical Performance Battery
TIA:	Transient ischemic attack
VAS:	Visual Analogue Scale

1 OVERVIEW AND INVESTIGATIONAL PLAN

1.1 STUDY DESIGN AND RANDOMIZATION

The SPRINTT trial is a multicenter randomized controlled trial (RCT) involving a multicomponent intervention (MCI) (based on Physical Activity (PA) and nutritional counselling/dietary intervention, and information and communication technology [ICT] intervention) versus a healthy aging lifestyle education (HALE) programme in 1500 non-disabled, community-dwelling persons age 70 years and older with physical frailty and sarcopenia (PF&S) across nine European countries.

The eligibility of participants is established in a multi-step screening process. Participants are first screened by phone or mail, the phone interview/mail being designed to exclude individuals who are clearly ineligible or unlikely to benefit from participation in the study. Then, at the first clinic screening visit, medical and functional exclusions criteria are assessed. A second screening clinic visit is possible if medical information is considered insufficient at the first visit, in order to definitively determine the eligibility of the participant.

Each eligible participant is then randomized to one of the two interventions arms of the clinical trial (MCI or HALE group) using a ratio 1:1, with permuted-block randomization list through a centralized allocation system.

Randomization can only occur after all eligibility requirements have been met and all key baseline data have been entered in the SPRINTT application. Randomization is stratified by study site, gender, and SPPB category (i.e., <8 or ≥8). The algorithm has been designed to randomize 80% of patients with a SPPB category < 8.

Approximately 1500 participants (750 in each intervention arm) were expected to be recruited and randomized from approximately 17 sites in 11 European countries.

1.2 OBJECTIVES

1.2.1 Primary objectives

The primary objective of this study is to evaluate the effectiveness of a multicomponent intervention (MCI) programme (physical activity [PA], nutritional counselling/dietary intervention, and information & communication technology [ICT] intervention) compared with a healthy aging lifestyle education (HALE) programme on the hazard rate of mobility disability assessed by the inability to complete a 400-m walk test in less than 15 minutes without sitting, help from another person or use of a walker, in non-disabled older people with physical frailty and sarcopenia (PF&S).

1.2.2 Secondary objectives

The secondary objectives are to evaluate the effect of the MCI programme compared with the HALE programme on relevant health-related outcomes, in particular:

- Changes in physical performance measures,
- Changes in disability status,
- Changes in body composition, anthropometric and nutritional measures,
- Changes in cognitive function and mood,
- Incidence of falls and injurious falls
- Modifications of quality of life
- Difference in use of healthcare services
- Difference in mortality

A cost-effectiveness analysis is also planned and will be conducted by a dedicated team within work package 5.

1.3 DETERMINATION OF SAMPLE SIZE

The sample size calculation was based on the LIFE-M study database (1, 2). Specifically, survival analyses were run according to different levels of Short Physical Performance Battery (SPPB) score (lower than 8, versus 8 and 9). The outcome considered was major mobility disability (i.e. inability to complete the 400-m walk test). The effect of PA on major mobility disability was negligible in participants with a baseline SPPB score ≥8 (hazard ratio=0.94; 95% confidence interval [CI]: 0.72-1.23; p=0.65). Conversely, the hazard ratio was statistically significant in participants with a baseline SPPB score lower than 8 (hazard ratio=0.75; 95%CI: 0.59-0.94; p=0.012).

- A sample of 1,200 older persons with SPPB score < 8 enrolled over 12 months is estimated to provide 85% power (434 events) to detect a 25% reduction in the hazard of major mobility disability over a maximum of 36 months of follow-up, considering an exponential dropout rate of 25% over 2 years and a log rank test with a 5% two-sided alpha level. An interim blinded sample size reassessment after 11 months from the beginning of recruitment allows taking immediate actions to preserve the study power in case the efficiency of enrolment or the number of observed events is not coherent with the original assumptions.

- In addition to not restrict the enrolment to older people with SPPB score lower than 8 that would jeopardize the aim of project to comprehensively characterize the PF&S condition and to not decrease the study power hence, the probability of success of the trial, it was decided to include in the study a convenience sample of 300 older adults with baseline SPPB score 8 or 9. An ad hoc hierarchical testing procedure has been formulated to control type I error: first, we will compare the effects of the

interventions in participants with SPPB score lower than 8 and, only in case of significant result (i.e., two sided p<5%), the comparison will be extended to the whole study population

- At the first blinded sample size reassessment (may 2017) it was decided to increase the accrual period by 6 months, but maintaining a maximum of 36 months of follow-up period
- The last participant was randomized on November 15th 2017 (1205 in SPPB<8 stratum, 314 in SPPB 8-9 stratum)
- A second blinded power reassessment was performed on May 7th 2018, and revealed a lower than expected number of events (215 vs. 270). Therefore, in order to maximize the probability to reach the requested number of event (434) at the end of the study, the study duration has been increased by 7 months.

1.4 STUDY PLAN

Approximately 1500 participants will be randomized in a ratio 1:1 to the following two treatment groups:

- HALE programme (n = 750 ; approximately n=600 with SPPB<8 and n=150 with SPPB≥8)
- MCI programme (n = 750 ; approximately n=600 with SPPB<8 and n=150 with SPPB≥8))

In the MCI intervention group, the PA component is designed to be performed both at the center and at home. Participants will train at the center twice a week under direct supervision of instructors. The PA schedule below shows the expectation in term of center-based sessions and home-based exercises.

Table 1 – Intervention staff contacts for the PA group

Week	Center-based PA	Home-based PA
Adoption (week 1-52)	2 times each week	1time/week (week 1-4)<br>2 times/week (week 4-8)<br>Up to 3-4 times/week (week 8-52)
Maintenance (weeks 53 – end of the trial)	2 times each week	Up to 3-4 times/week

Week	Center-based PA	Home-based PA

The total amount of PA has to be monitored by investigators using results of actimetry from the ActivPAL^{TM} device (measured at baseline and every 6 months [±2 weeks]).
In addition, the local dietician/nutritionist (D/N) will train each participant randomized to the MCI group on how to complete a 3-day dietary record. The 3-day dietary record will be collected from each participant in the MCI intervention group at baseline and every 12 months. This assessment will then support the elaboration of personalized nutritional recommendations by the local D/N, in agreement with national and international guidelines.

In the HALE programme arm, participants will meet in small groups, two times per month with required participation at least one per month. The HALE programme is based on workshop series, during which participants receive information on a variety of topics of relevance to older adults. At the end of the class, there is also 5-10 minutes of instructor-led programme of upper extremity stretching exercises or some relaxation techniques.

The participants will have a different length of follow-up according to the date of his/her recruitment. Those randomised at the beginning of the recruitment phase will have a longer follow-up duration (up to 3 years) compared with those included at the end of recruitment (minimum 2 years of follow-up).

The study flow chart of the administration of questionnaires and measures according to the clinic visits scheduled is detailed in section 7 of the protocol.

1.5 MODIFICATIONS TO THE STATISTICAL SECTION OF THE PROTOCOL

This section summarizes major changes to the protocol statistical section with emphasis on changes after study start (after the first participant was enrolled).

The statistical section of the protocol was not changed in an amendment

1.6 STATISTICAL MODIFICATIONS MADE IN THE STATISTICAL ANALYSIS PLAN

Not applicable

2 STATISTICAL AND ANALYTICAL PROCEDURES

2.1 ANALYSIS ENDPOINTS

2.1.1 Demographic and baseline characteristics

The baseline value is defined as the last available value up to the randomization visit V1 – T0 or date of randomization if V1 is missing (included).

All baseline safety and efficacy parameters (apart from those listed below) are presented along with the on-study summary statistics in the safety and efficacy sections (Section 2.4.5 and Section 2.4.4).

Demographic characteristics

Demographic variables are gender (Male, Female), race (White, Black or African American, Asian, American Indian or Alaska Native, Native Hawaiian or other Pacific Island, Other), age in years (quantitative and qualitative variable : <70, [70 – 75[, [75 – 80[, [80 – 85[, [85 – 90[, ≥ 90), ethnicity (Hispanic or Latino, Not Hispanic or Latino, Not reported, Unknown), and country. In addition to demographic variables, height, weight and BMI (quantitative and qualitative variable : <18.5, [18.5 – 25[, [25 – 30[, [30 – 35[, [35 – 40[, ≥ 40) will be presented as baseline characteristics.

Medical or surgical history

Medical history and medical findings include:

Physical examination (normal/abnormal/exclusion): skin, lungs, heart, extremities, neurological System, vascular system (present/absent).

Medical (or surgical) histories (Y/N/Refused/Don't know):

- Any cardiovascular medical history: High blood pressure or hypertension; heart attack, or coronary, or myocardial infarction leading to overnight hospitalization; heart failure or congestive heart failure; angina or chest pain due to blockages in the arteries of the heart; coronary artery bypass surgery; stent and/or angioplasty of the coronary arteries; carotid endarterectomy; pacemaker

- Any diabetes medical history: Diabetes, sugar in urine, or high blood sugar; oral antidiabetic drugs intake; use of insulin injections

- Any neurologic medical history: transient ischemic attack (TIA) or mini-stroke ; stroke or brain hemorrhage leading to hospitalization ; difficulty from the stroke (Arm and/or leg still weak/hard to use, Trouble walking, Trouble with speech and/or Other)

- Any cancer or malignant tumor, excluding minor skin cancer
- Any musculoskeletal medical history: broken or fractured hip leading to hospitalization, other broken of fractures bones since the age of 50 (wrist/arm/back or spine/other bones);
- Any arthritis medical history during the last 6 months: arthritis or rheumatism; any pain and/or stiffness in the joints (hands or fingers/shoulders/knees/hips/back or spine/foot)
- Any cirrhosis or liver disease
- Any chronic lung disease (chronic bronchitis, COPD, asthma, or emphysema)
- Any emotional, nervous or psychiatric problems, since the age of 50
- Any fall over the past year and number of times (1-3 times/4 or more falls), injurious falls requiring doctor, emergency room, hospital or urgent care center in past year
- Other predefined medical history in the 5 past years inquiry from physician or medical professional advice regarding: back injury, paralysis, fainting or passing out, shortness of breath, asthma, chest congestion/cough, abnormal heart rhythm, depression, foot ulcer, a wound that would not heal
- Any symptoms in the past 6 months of: anxiety, fatigue, decreased appetite, insomnia, dizziness, muscle or joint stiffness, muscle strain or soreness, sprain (ankle or knee), foot pain
- Any other medical conditions that might affect ability to participate in a physical activity program
- Other medical and surgical history in the last 5 years: this information will be coded using the version of Medical Dictionary for Regulatory Activities (MedDRA) currently in effect at Sanofi at the time of database lock.

Disease characteristics at baseline

Specific disease history includes:

- The SARC-F total score range from 0: best to 10: worst (quantitative and qualitative variable: <3, 3, 4, ≥ 5). A score equal to or greater than 4 is predictive of sarcopenia.
- The 400-meter walk test duration at baseline (min) and 400-m walk speed (m/s), (see details in Section 2.5.2),

- The local and central reading of the Appendicular lean mass (aLM) (kg) and aLM^{BMI} (Body mass index-adjusted appendicular lean mass) by gender assessed by Dual energy X- ray absorptiometry (DXA). Discrepancies between local and central readings assessed by aLM >0.5 or <-0.5 kg or delta aLM^{BMI} > 0.05 or <-0.05 will be summarized by categories

- The SPPB total score (quantitative and qualitative variable: <8, ≥ 8; scale from 0: low performance to 12: high performance)

- The Mini Mental State Examination (MMSE) total score (scale from 0: severe cognitive impairment to 30: normal cognitive function)

- The Cumulative Illness Rating Scale (CIRS): Total score, severity index and comorbidity index.

Any technical details related to computation are described in Section 2.5.

2.1.2 Prior or concomitant medications

All medications taken by the participants during the screening visit and for each subsequent visits all medications taken in the past two previous weeks before the visit, including ATC coded medications and non ATC coded medications, are to be reported in the case report form pages.

All ATC coded medications will be coded using the World Health Organization-Drug Dictionary (WHO-DD) using the version currently in effect at Sanofi at the time of database lock.

- Prior medications are those the participant reported at the screening visit. Prior medications can be discontinued before the first intervention or can be ongoing during the study.
- Concomitant medications are any treatments reported by the participant at any post-randomization visits (i.e. from V2-Month 3).

2.1.3 Efficacy endpoints

The observation period of efficacy data is from the randomization visit V1-T0 or date of randomization if V1 is missing (excluded) until the end of the study.

Except the 400-meter walk tests, all measurements, scheduled or withdrawal visit (V9), will be assigned to analysis windows defined in Section 2.5.4 in order to provide an assessment of the efficacy parameters for Month 3 to Month 36 time points.

The baseline value is the last available and valid measurement obtained up to the randomization visit V1-T0 or date of randomization if V1 is missing (included).

2.1.3.1 Primary efficacy endpoint(s)

The primary endpoint is the time (in days) from randomization to the date of first occurrence of major mobility disability assessed by the inability to complete the 400-meter walk test within 15 minutes, without sitting, help from another person or use of a walker.

This time is calculated as date of event minus date of randomization visit V1-T0 (or date of randomization if V1 is missing) + 1 day.

The 400-meter walk test is assessed at Month 3 then every 6 months after the randomization visit.

Adjudication of primary endpoint

All 400-meter walk tests not performed (including missing tests due to visits performed by phone or proxy) will be adjudicated. A pre-defined algorithm will be applied to automatically adjudicate the missing tests (i.e. disability ascertained or no disability). Following this automatic adjudication, all remaining missing tests will be then evaluated by an adjudication committee.

The algorithm was built by identifying all the information recorded in the eCRF that was related to the participants' walking ability. Once selected, all this information (e.g. answers to questionnaires, results from the gait speed test, etc.) was then included in a logistic regression model run on all available 400-meter walk tests in order to determine which information was most predictive of the disability status (i.e. success or failure).

To adopt a conservative approach, the selected information was then fine-tuned to limit the number of tests wrongly classified as no disability to 5%. The number of tests wrongly classified as disability reaches 25%.

The resulting algorithm, based on result from the Gait speed test and some answers obtained from the ADL Proxy and Assistive Device questionnaires, is as follows:

1. "Disability ascertained" if:
    - Gait speed test performed in 0.4 m/s or less ***OR*** participants not able to walk the 4 meters ***OR*** had to use an aid other than a cane to walk
    - ***OR*** participants answer "*Yes*" to at least one of the following questions:
        - *"When the subject walks outside his/her home, does he/she usually require a walker?"*
        - *"When the subject walks outside his/her home, does he/she usually require a tripod or a quad cane?"*
        - *"When the subject walks inside his/her home, does he/she usually require a walker?"*
        - *"When the subject walks inside his/her home, does he/she usually require a tripod or a quad cane?"*

- ***OR*** participants answers "*Unable to do*" or "*A lot of difficulty*" to at least one of the following questions:
  - *"Walking for 400 metres, which is a little shorter than half a kilometre?"*
  - *"Walking for more than one kilometre because of your health?"*
- ***OR*** participants answers "Unable to do" or "A lot of difficulty" or "*Some difficulty*" to the question "*Walking 100 metres because of your health?*"

2. "No disability" if:
   - Gait speed test performed in more than 0.67 m/s (less than 6 sec to walk 4 meters) without an aid other than a cane ***OR*** Lawton ADL score equal to 8
   - ***AND*** participants answer "*No difficulty*" to the 2 following questions
     - *"Walking for 400 metres, which is a little shorter than half a kilometre?"*
     - *"Walking 100 metres because of your health?"*
   - ***AND*** participants answer "No difficulty" or "A little difficulty" or "*Some difficulty*" to the question "*Walking for more than one kilometre because of your health?*"

Then, the adjudication committee will assess remaining missing 400-m walk tests using all information in the following available eCRF modules:

- 400-meter walk test
- Assistive device questionnaire
- PAT-D
- ADL Proxy
- Outcome events
- Adverse events

The 3 possible assessments by the adjudication committee are "Disability ascertained", "No disability" or "Disability not conclusive" (i.e. when the outcome could not be adjudicated due to the lack of available information)

<u>Derivation of primary endpoint</u>

Disability will be considered ascertained:

- at the date of the 400-meter walk test, if participants fail to walk 400 meters within 15 minutes, or need an assistive device
- or at the date of the visit (or date of the 400-meter walk test if available) when the visit is performed but the participant did not perform the 400-meter walk test and the adjudication (automatic or from the committee) does not classified the event as no disability (i.e. ascertained or not conclusive) if any

- or at the date of death if the participants died during the study

Participants who have not met this disability criterion at the end of their study participation (including withdrawals) will be considered as right-censored observations. The censoring time will be calculated as the date of their last successful completed 400-meter walk test (including assessment of "no disability" by the automatic adjudication or the adjudication committee) minus the date of randomization visit V1-T0 (or date of randomization if V1 is missing) + 1 day.

Particular cases: In the case where more than 9 months are observed between two consecutive visits or between the last available visit and the death:

- If the disability criterion is not meet at the 2nd consecutive visit, then participants will be censored at the date of their first consecutive visit. In the case where the first of the two consecutive visits is the randomization visit, then the censoring time will be equal to 1.

- If the disability criterion is observed at the 2nd visit of the two consecutive visits, or the participant died more than 9 month after the last available visit, then the disability will be considered ascertained at the date of the first consecutive visit or at the date of the last available visit before to die, plus 183 days (i.e., 6 months). In the case where the first of the two consecutive visits (or the last available visit before to die) is the randomization visit, then the disability will be considered ascertained at the date of randomization visit V1-T0 (or date of randomization if V1 is missing) + 183 days.

If no post-baseline visit is done, then participants will be censored at the date of randomization visit V1-T0 (or date of randomization if V1 is missing).

2.1.3.2 Secondary efficacy endpoint(s)

The timing of all secondary endpoints assessments are specified in the study flowchart (in Appendix A).

The secondary efficacy endpoints include:

- Changes in physical performance measures from baseline to Month 24 and Month 36, assessed by:
  - The total SPPB score computed directly in the corresponding e-CRF page (ranging from 0: low performance to 12: high performance)
  - The usual gait speed in m/s, assessed by the ratio of the 4 meters walks divided by time (faster of the two walks of the gait speed test)(see details in Section 2.5.2)
  - The handgrip strength (maximum reading in kg)

- Changes in disability status from baseline to Month 24 and Month 36, assessed by:
    - The Lawton IADL score (ranging from 0 to 8; the higher the score, the greater the participant's abilities) obtained from the Pepper Assessment Tool for Disability (PAT-D) questionnaire computed directly in the corresponding e-CRF page
    - The KATZ ADL score obtained from the PAT-D questionnaire (ranging from 0 [participant totally dependent] to 6 [participant independent])
- Time (in days) from the randomization to the date of first occurrence of persistent mobility disability, operationalized as the failure to complete the 400-metre walk test within 15 minutes or as the assessment of disability by the adjudication in two consecutive visits, or as a first mobility disability followed by the death:
    - Persistent mobility disability will be considered ascertained:
        - at the date of the first test failure among the 2 consecutive available test failures, whatever the time interval between the 2 consecutive visits, if the first test failure is observed no more than 9 months after the previous test success
        - Or at the date of the last available test failure before the date of death, whatever the time interval between the failure and the death, if the test failure is observed no more than 9 months after the previous test success
        - Or at the date of the success test (including baseline test) + 183 days (i.e. months 6) if a persistent disability (i.e. 2 consecutive observed failure tests whatever the time interval between the 2 consecutive visits, or one failure test followed by the death) is observed more than 9 months after the previous test success
    - Participants will be censored:
        - at the date of last available test (failure or success) or date of death if no persistent mobility disability observed and no more than 9 months between two consecutive visits or between the last available successful test and the death,
        - or at the date of the first test (failure or success) where the following test is observed more than 9 months after the previous test, and no persistent disability is observed.

Notes: The baseline assessment is considered as a successful test in this endpoint definition.

If no post-baseline visit is done, then participants will be censored at the date of randomization visit V1-T0 (or date of randomization if V1 is missing).

- Changes in body composition measures from baseline to Month 24 and Month 36, assessed using DXA (centralized reading):

- aLM^{BMI}, in males and females
- aLM in kg, in males and females

- Changes in anthropometric parameters from baseline to Month 24 and Month 36, assessed by:
  - Weight in kg, assessed using percent change
  - Body mass index (BMI) in kg/m^2,
  - Calf circumference in cm,
  - Waist circumference in cm,
  - Hip circumference in cm,
  - Mid-arm circumference in cm
- Changes in nutritional status from baseline to Month 24 and Month 36 assessed by the Mini Nutritional Assessment-Short Form (MNA-SF) score computed directly in the corresponding e-CRF page (ranging from 0 to 14: a score of 12 or greater indicating satisfactory nutritional status and a score strictly below 8 indicating malnutrition)
- Changes in cognitive function from baseline to Month 24 and Month 36, assessed by the Mini Mental State Examination (MMSE) score computed directly in the corresponding e-CRF page (ranging from 0 to 30: a score of 27 or greater indicating normal cognitive function, between 19 and 24 indicating mild cognitive impairment, between 10 and 18 indicating moderate cognitive impairment and a score of 9 or below indicating severe cognitive impairment)
- Changes in Cumulative Illness Rating Scale (CIRS) from baseline to Month 24 and Month 36, computed directly in the corresponding e-CRF page:
  - Total score (ranging from 0 to 56)
  - Severity index (ranging from 0 to 4)
  - Comorbidity index (ranging from 0 to 14)
- Changes in mood from baseline to Month 24 and Month 36 using the Center for Epidemiological Studies-Depression scale (CES-D):
  - The score obtained with the 11-item version of the CES-D will be transformed using the procedure recommended by Kohout (3) to make it compatible with the full 20-item instrument (see details in Section 2.5.2). Total scores range from 0 to 60, with higher scores indicating more depressive symptoms.
- Time (in seconds) to complete the Trail Making Test (TMT) of visuo-motor speed (part A) and executive function (part B)
- 7-day average of total steps, sitting and lying activity (hours), standing activity (hours) and stepping activity (hours), from ActivPal accelerometry

2.1.4 Safety endpoints

The safety analysis will be based on the reported adverse events and other safety information, such as clinical laboratory data and vital signs.

The observation period of safety data is from the randomization visit V1-T0 or date of randomization if V1 is missing (excluded) until the end of the study.

All measurements of safety information (except adverse events and deaths), scheduled or withdrawal visit (V9), will be assigned to analysis windows defined in Section 2.5.4 in order to provide an assessment of the safety parameters at each post-baseline time point planned to be collected as per protocol.

2.1.4.1 Adverse events variables

The following adverse events (AE) are to be collected during the study:

- Serious AEs,
- Unexpected AEs that may be related to study procedures,
- AEs that occurred while under the supervision of guidance of study related personnel
- AE of special interest (AESI)

Adverse event observation period:
AEs are classified into three distinct categories:

- Pre-emergent adverse events are adverse events that occurred from the signed informed consent date up to the randomization visit V1-T0 or date of randomization if V1 is missing (included)
- Emergent adverse events (EAE) are adverse events that occurred from the randomization visit V1-T0 or date of randomization if V1 is missing (excluded) up to the end date of study participation (included)
- Post-emergent adverse events are adverse events that occurred after the end date of study participation

All adverse events are coded to a lower-level term (LLT), preferred term (PT), high-level term (HLT), high-level group term (HLGT), and associated primary system organ class (SOC) using the version of Medical Dictionary for Regulatory Activities (MedDRA) currently in effect at Sanofi at the time of database lock.

In SPRINTT study, SAEs criteria include the following events:

- Death,
- In-patient hospitalization
- Life threatening illness or accident,

- Permanent disability or incapacity
- Other serious illness that might have results in an SAE without aggressive medical intervention

AEs that occurred while under the supervision or guidance of study related personnel include:

- AE that meets criteria for SAE
- Event requiring active intervention by research staff to reduce potential harm
- Chest pain for more than two minutes after stopping exercise
- Dyspnea for more than two minutes after stopping exercise
- Vital signs out of range (systolic BP >= 250 mmHg or diastolic >= 115 mmHg, HR>120 beats/minute or < 45 beats/minute) for more than two minutes after stopping exercise
- A fall during study recommended activity
- A symptom or illness that developed and required medical management or attention

Adverse events of special interest include:

- Emergency room or urgent care visit
- Fracture
- Outpatient surgery
- Abnormal laboratory or diagnostic test result requiring immediate medical attention
- Restricted activity due to health problem potentially related to the study activity that led an inability to leave home for at least one week

2.1.4.2 Falls

Falls will be described using information collected in the Outcome events eCRF form:

- Since the last visit, participants who broke a bone as a result of a fall (Yes/No/Refused/Don't know)
- Since the last visit, participants who fallen, and participants who fallen resulting in an inability to leave home for at least one week (Yes/No/Refused/Don't know)

Injury falls will be identified using a customized MedDRA searches

2.1.4.3 Deaths

The deaths observation period is from the date of randomization until the end of the study participation.

The safety endpoint mortality will also be assessed using the time from randomization to the date of death, as reported in an adverse event form by the investigator.

Participants not dead or lost to follow-up at the end of the study will be censored at the date of the study end or date of last news reported in the end-of-study form.

2.1.4.4 Laboratory safety variables

Clinical laboratory data consists of blood analysis, including hematology, clinical chemistry, and urinalysis.

Blood samples for clinical laboratories will be taken at randomization visit V1-T0, M12, M24, M36 and, if applicable, at the end-of-study visit. The laboratory parameters will be classified as follows:

- Hematology
  - **Red blood cells and platelets and coagulation**: hemoglobin, hematocrit, Ery. mean corpuscular volume, Ery. mean corpuscular hemoglobin concentration, Ery. mean corpuscular hemoglobin, red blood cells count, platelet count.
  - **White blood cells:** white blood cells count, neutrophils, lymphocytes, monocytes, basophils, eosinophils
- Clinical chemistry
  - **Metabolism:** glucose, total cholesterol, HDL-cholesterol, LDL-cholesterol, triglycerides, total protein, albumin,
  - **Electrolytes**: sodium, potassium, calcium
  - **Renal function**: creatinine, blood urea nitrogen
  - **Liver function**: alanine aminotransferase (ALT), aspartate aminotransferase (AST), total bilirubin
  - **Vitamin D concentration**

2.1.4.5 Vital signs variables

Vital signs include: heart rate (bpm), sitting systolic and diastolic blood pressure (mmHg) are recorded at screening, M3, M6, M12, M18, M24, M30, M36 and at the end of the study (if no evaluation in the past 3 months).

2.1.5 Quality-of-life endpoints

In SPRINTT, participants' quality of life will be assessed at each clinical visit using the EuroQoL-5D instrument. The EuroQoL-5D is a 5-item questionnaire exploring the following dimensions: Mobility capacity, Self-care, Usual activities, Pain/Discomfort, Anxiety/Depression.

Each item can take one out of five possible degrees of impairment/severity and are coded from '1': no problems to '5': extreme problems. The EuroQoL-5D index utility score is calculated using the crosswalk method based on the UK value set (4).
The EuroQoL instrument also includes a 10-cm long visual analogue scale (VAS). Participants will thus be asked to draw a line from a box to the point on the thermometer-like scale (ranging from 0 [worst quality of life] to 100 [best quality of life]) "corresponding to their health-related quality of life".

2.1.6 Health economic endpoints

Heath economic components and analyses, including cost-effectiveness analysis, will be detailed in a separate dedicated document.

2.1.7 Biomarker endpoints

Biomarkers endpoints and analyses will be detailed in a separate dedicated document.

2.2 DISPOSITION OF PARTICIPANTS

This section describes participant disposition for both participant study status and the analysis populations.

Screened participants are defined as any participant who signed the informed consent.

Randomized participants consist of all participants with a signed informed consent form and who have been randomized by the system in one of the intervention arms, whatever the intervention attendance.

For participant study status, the total number of participants in each of the following categories will be presented in the clinical study report using a flowchart diagram or summary table:

- Screened participants
- Screen failure participants and reasons for screen failure as reported by the investigators in the inclusion criteria and exclusion criteria e-CRF pages
- Randomized participants
- Participants who completed the study (i.e. participants who were followed for at least 2 years)

- Participants who did not complete the study (i.e. participants who were followed for less than 2 years)
- Participants who discontinued study by main reason for study discontinuation

All categories of participants (except for the screened and nonrandomized categories) will be presented by intervention arm and the percentages will be calculated using the number of randomized participants within each intervention arm as the denominator. Reasons for study discontinuation will be supplied in a table giving numbers and percentages by intervention arm, as well as, displayed in a listing.

A summary of the distribution of participants by country and site will also be provided (overall number of patients screened, randomized, as well as number of participants who discontinued from study.

All critical or major deviations will be summarized in tables giving numbers and percentages of deviations by intervention arm.

Additionally, the following analysis study populations will be summarized by intervention arm.

- All randomized participants
- All randomized participants with a baseline SPPB score <8 assigned by randomization
- All randomized participants with a baseline SPPB score ≥8 assigned by randomization

The discrepancy between randomization strata assigned by randomization (i.e. the baseline SPPB score and the sex) and the information reported on e-CRF will be summarized for all randomized patients

2.3 ANALYSIS POPULATIONS

The randomized population includes any participant who has been allocated to a randomized programme whatever the number of intervention attendance.

For any participant randomized more than once, only the data associated with the first randomization will be used in any analysis population. The safety experience associated with any later randomization will be assessed separately.

The main analysis population for all analyses (i.e. efficacy, safety and quality-of-life) will be the randomized population with a baseline SPPB score < 8 as assigned by randomization. Participants will be analyzed according to the intervention arm allocated by randomization.

The secondary analysis population will include all randomized participants with a baseline SPPB score >= 8 as assigned by randomization and analyzed according to the intervention arm allocated by randomization.

2.4 STATISTICAL METHODS

2.4.1 Demographics and baseline characteristics

Continuous data will be summarized using the number of available data, number of missing values, mean, standard deviation (SD), median, minimum, and maximum for each intervention arm. Categorical and ordinal data will be summarized using the number and percentage of participants in each intervention arm.

Parameters will be summarized on the randomized population analyzed in the intervention group to which they were randomized. Analyses will be provided in the randomized population according to the baseline SPPB categories (<8, ≥ 8) assigned by randomization.

Parameters described in Section 2.1.1 will be summarized by intervention arm and overall using descriptive statistics.

Other medical/surgical history will be summarized in each intervention arm by primary SOC, HLT and PT. Events will be sorted by SOC internationally agreed order and decreasing frequency of HLT and PT based on the overall incidence of participants with a baseline SPPB < 8 assigned by randomization.

P-values on demographic and baseline characteristic data will not be calculated.

No specific description of the efficacy parameters, safety parameters and quality-of-life parameters will be provided at baseline. If relevant, baseline values will be described along with each efficacy, safety or quality-of-life analysis, respectively.

2.4.2 Prior or concomitant medications

The prior and concomitant medications will be presented for the randomized population. Analyses will be provided in the randomized population according to the baseline SPPB categories (<8, ≥ 8) assigned by randomization.

The number of participants who reported at least one ATC coded medication (prior and concomitant separately), as well as the number of participants who reported at least one non-ATC coded medication (prior and concomitant separately) will be presented.

ATC coded medications will be summarized by intervention arm according to the WHO-DD dictionary, considering the first digit of the anatomic category (ATC) class (anatomic category) and the first 3 digits of the ATC class (therapeutic category).

All ATC codes corresponding to a medication will be summarized, and patients will be counted once in each ATC category (anatomic or therapeutic) linked to the medication. Therefore, patients may be counted several times for the same medication

The table for prior ATC coded medications will be sorted by decreasing frequency of ATC followed by all other therapeutic classes based on the incidence of overall randomized population with a baseline SPPB score <8 assigned by randomization. In case of equal frequency regarding ATCs (anatomic or therapeutic categories), alphabetical order will be used.

The tables for concomitant medications will be sorted by decreasing frequency of ATC followed by all other therapeutic classes based on the incidence in the MCI intervention group with a baseline SPPB score < 8 assigned by randomization. In case of equal frequency regarding ATCs (anatomic or therapeutic categories), alphabetical order will be used.

In addition, the repartition of the participants according to the number of ATC coded medications reported at screening (i.e. prior medications), as well as the number of ATC coded medications reported post-randomization visit (i.e. concomitant medications) will be summarized by visit.

2.4.3 Intervention attendance and compliance

For each participant, in both intervention arms, the number of attended sessions and the number of expected attended sessions should be reported each month for the following type of sessions:

- Center-based PA attendance (MCI group)
- Home-based PA attendance (MCI group)
- HALE attendance (HALE group)

The intervention attendance and compliance will be assessed and summarized by intervention arm in the randomized population according to the baseline SPPB categories (<8, ≥8) assigned by randomization.

2.4.3.1 Intervention attendance

In the MCI intervention arm, both the center-based physical exercise attendances and the home-based physical exercise attendances will be summarized.

In the HALE intervention arm, the number of sessions attended will be summarized.

For each type of sessions, the number of participants who attended at least one session, the total number of sessions attended (quantitative), and the total number of sessions per months (quantitative) will be described.

The total number of sessions attended per month will be derived as follows:

$$\frac{\text{Total Number of sessions done since the beginning of the study}}{\text{Number of months}}$$

2.4.3.2 Compliance

In the MCI intervention arm, both the center-based physical exercise compliance and the home-based physical exercise compliance will be summarized.

In the HALE intervention arm, compliance will be summarized.

For each type of sessions, the compliance will be calculated per participant according to the information recorded by investigators in the e-CRF page as follows:

$$\frac{\text{Total Number of sessions attended since the beginning of the study}}{\text{Total Number of sessions expected since the beginning of the study}} \times 100$$

In addition to the attendance compliance for the MCI group, the collection of a 3-day dietary record (Y/N) will be summarized overtime.

2.4.4 Analyses of efficacy endpoints

2.4.4.1 Analysis of primary efficacy endpoint(s)

The analysis of the primary efficacy endpoint will be the comparison between the two intervention arms using a two-sided 0.05 alpha level log-rank test procedure stratified by randomization stratification factors (i.e., study site and gender).

The primary comparison will be conducted in the randomized participants with a baseline SPPB score <8 assigned by randomization.

The hazard ratio between intervention groups and the corresponding 95% confidence interval will be estimated using a Cox proportional hazard model stratified by the same stratification factors as those used for the log-rank test described above.

The underlying assumption of proportional hazards for Cox model will be checked graphically by plotting natural logarithm of the cumulative hazard Kaplan-Meier estimate (Log(-Log (KM survival function estimates))) versus natural logarithm of time for each intervention arm.

If proportionality is not observed on primary analysis, sensitivity analyses will be performed by including a time-dependent variable with the corresponding interaction.

The cumulative incidence functions for the 2 intervention arms will be summarized using Kaplan-Meier method and displayed graphically. The cumulative incidence rate over time at each scheduled time-points of the protocol together with appropriate 95% confidence interval will be provided.

Reason for censoring will be summarized by intervention arms.

If this primary analysis performed in the randomized population with a baseline SPPB score <8 is statistically significant (p<0.05), the comparison will be extended to include the remaining participants with baseline SPPB 8-9 only if the interaction between the baseline SPPB randomization stratum and the intervention arm can be ignored. The hazard ratio between intervention groups and the corresponding 95% confidence interval will be then computed using a Cox proportional hazard model stratified by randomization strata (site, gender, baseline SPPB) for the comparison in the whole randomized population.

The interaction between the baseline SPPB randomization stratum and intervention arm will be assessed graphically by comparing, between the two baseline SPPB categories (<8 and ≥8), the cumulative incidence functions for the 2 intervention arms using the Kaplan-Meier method.

Subgroup analyses

The consistency of the results from the primary analysis conducted in the randomized participants with a baseline SPPB score <8 assigned by randomization will be evaluated across pre-defined subgroups in patients with available results. The subgroups are:

- Gender (Female; Male)
- Race (White ; All others race)
- Age (<80; ≥80 years)
- History of cardiovascular disease (Yes; No)
- History of diabetes (Yes; No)
- Gait speed (<0.8; ≥0.8 m/sec)

For each subgroup, the hazard ratio and its associated 95% confidence interval will be estimated using the same Cox proportional hazards model applied for the primary analysis and by adding terms for the subgroup, intervention group and subgroup-by-intervention. A forest plot summarizing the results for each subgroup will be provided.

When the subgroup considered is equal to the randomization stratum of gender, the stratification by the randomization stratum of gender will be removed from the model.

Supportive analysis

A supportive analysis will be performed by excluding deaths from the definition of the disability event (described in Section 2.1.3.1). Participants who have not met the disability criterion before to die will be considered as right-censored observations. The censoring time will be calculated as the date of their last successful completed 400-meter walk test minus the date of randomization visit V1-T0 (or date of randomization if V1 is missing) + 1 day.

Same methodology as described above in Section 2.4.4.1 for the primary efficacy endpoint will be used to analyze the time (in days) from randomization visit V1-T0 (or date of randomization if V1 is missing) to the date of first occurrence of major mobility disability using the new disability definition that excludes deaths.

2.4.4.2 Analyses of secondary efficacy endpoints

The change from baseline (or percent change for weight) for all continuous variables with a normal distribution described in Section 2.1.3.2 will be analyzed in the randomized population with a baseline SPPB score <8 using mixed-effect models with repeated measures (MMRM) approach. All post-baseline data available within Month 3 to Month 36 analysis window as defined in Section 2.5.4 will be used.

These models will include the fixed categorical effects of intervention arm (MCI versus HALE), the planned time point (Month 3 to Month 36), the randomization strata of site and gender, the intervention-by-time point interaction as well as, the continuous fixed covariates of baseline value and baseline value-by-time point interaction. For change in body composition measures, assessed using DXA (centralized reading) (aLM^{BMI}, and aLM), the models will be assessed separately for males and females.

These models will be run using SAS Mixed procedure with an unstructured correlation matrix to model the within-participant errors. Parameters will be estimated using restricted maximum likelihood method with the Newton-Raphson algorithm. Denominator degrees of freedom will be estimated using Satterthwaite's approximation.

These models will provide adjusted least-squares means estimates at Month 24 and Month 36 for both intervention arms, as well as the difference of these estimates, and their corresponding standard errors (SEs) and 95% confidence intervals (CI).

In addition to these models, results per time point for all planned time points visits (value at visits using analysis windows, and changes from baseline) will be summarized and plotted for all quantitative secondary endpoints using LS mean and SE for each intervention group obtained from the same MMRM models as used for endpoints above and with raw values and changes from baseline as response variable in the model as appropriate. For weight, similar summary will be provided, but the percent change from baseline will be presented instead.

Time to first occurrence of persistent mobility disability will be compared, as for the primary analysis endpoint, in the randomized population with a baseline SPPB score <8 within the intervention arm, using a two-sided Log-rank's test stratified by study site and gender. Hazard ratio (and 95% CI) between intervention groups will be estimated using a Cox proportional hazard model with the stratification factors of study site and gender as covariates.

The cumulative incidence functions for the 2 intervention arms will be summarized using Kaplan-Meier method and displayed graphically. The cumulative incidence rate over

time at each scheduled time-points of the protocol together with appropriate 95% confidence interval will be provided.

The time to complete the TMT-A test and the TMT-B test, as well as, the 7-day average of total steps, sitting and lying activity (hours), standing activity (hours) and stepping activity (hours) from ActivPal accelerometry will be summarized by visit using number, median, Q1, Q3, minimum and maximum. In addition, results overtime will be presented graphically using boxplots.

For other efficacy parameters not normally distributed, a non-parametric approach will be used. Descriptive statistics will be summarized using median, Q1, Q3, minimum and maximum.

All secondary endpoints analyses will be provided also in the randomized population with a baseline SPPB ≥8 assigned by randomization.

2.4.4.3 Multiplicity issues

In order to handle the multiple comparisons of the primary endpoint in the randomized population with a baseline SPPB score <8 and then in the whole randomized population, the type-I error will be controlled by the use of the sequential inferential approach. Only if the primary analysis in the randomized population with a baseline SPPB score <8 is statistically significant ($p<0.05$), the comparison of the primary endpoint between arms will be extended to the whole randomized population. The tests for the primary endpoint will be performed two-sided at level alpha equal to 0.5.

The secondary efficacy endpoints will be analyzed for exploratory purpose only.

2.4.5 Analyses of safety data

The summary of safety results will be presented by intervention arm in the randomized population with a baseline SPPB score <8 assigned by randomization, and in the randomized population with a baseline SPPB score ≥8 assigned by randomization separately.

The “observation period” defined in Section 2.1.4 is applicable to all safety analyses for the classification of AEs, determination of abnormality/potential abnormality values and the last on-study value for the laboratory and vital signs.

General common rules

All safety analyses will be performed on the randomized population, using the following common rules:

- All the data from the randomization visit V1-T0 (or date of randomization if V1 is missing) will be considered in safety analyses

- For each safety parameter, the baseline for each participant is defined as the screening visit value or the randomization visit (V1-T0) value, whichever is the latest one.
- Abnormality/potential abnormality values are defined as values considered medically important according to predefined criteria/thresholds defined in the protocol for clinical laboratory test and vital signs [Appendix B and Appendix C])
- Abnormality criteria will determine which participants had at least one post-baseline abnormality during the emergent adverse event period, taking into account all evaluations performed during the study, including nonscheduled or repeated evaluations. The number of all such participants will be the numerator for the post-baseline abnormality percentage
- The post-baseline abnormality denominator by intervention arm for a given parameter will be based on the number of participants who have that parameter assessed during post-baseline assessments.
- All measurements, scheduled or withdrawal visit (V9), will be assigned to analysis windows defined in Section 2.5.4 in order to provide an assessment for Month 3 to Month 36 time points.
- For quantitative safety parameters based on laboratory or vital signs measurements, descriptive statistics will be used to summarize results and change from baseline values by visit and intervention arm using analysis windows as defined in section 2.5.4.
- Summaries will include the last on-study value. The last on-study defined as the last available value collected at or just prior to the study end.

2.4.5.1 Analyses of adverse events

Generalities

The primary focus of adverse event reporting will be on the emergent adverse events.

If an adverse event date is incomplete, an imputation algorithm will be used to classify the adverse event as pre-emergent, emergent or post-emergent. The algorithm for imputing date will be conservative and will classify an adverse event as emergent unless there is definitive information to determine it is pre- or post-emergent. Details on classification of adverse events with missing or partial onset dates are provided in Section 2.5.3.

Adverse event incidence tables will present by SOC, HLGT, HLT, and PT, sorted by the internationally agreed order for SOCs and in alphabetic order for HLGT, HLT and PT within a SOC in alphabetical order for each intervention arm, the number (n) and percentage (%) of participants experiencing an adverse event and the number and rate of AEs per patient-year. The denominator for computation of percentages is the randomized

population with a baseline SPPB score <8, and the randomized population with a baseline SPPB ≥8 respectively, within each intervention arm.

Sorting within tables ensures the same presentation for the set of all AEs within the observation period (pre-emergent, emergent, post-emergent). For that purpose, the table of all emergent adverse events presented by SOC and PT sorted by the internationally agreed SOC order and decreasing frequency of PTs within SOCs will define the presentation order for all other tables unless otherwise specified. In case of equal frequency regarding PTs, alphabetical order will be used.

Analysis of all emergent adverse events

The following emergent adverse event summaries will be generated:

- Overview of emergent adverse events, summarizing number (%) of participants with at least one event and number and rate per patient-year for:
  - Pre-emergent adverse event
  - Emergent adverse event
  - Serious emergent adverse event
    - Death,
    - In-patient hospitalization
    - Life threatening illness or accident,
    - Permanent disability or incapacity
    - Other serious illness that might have results in an SAE without aggressive medical intervention
  - Emergent unexpected event that may be related to study procedures
  - Emergent adverse event that occurred while under the supervision or guidance of study related personnel
    - AE that meets criteria for SAE
    - Event requiring active intervention by research staff to reduce potential harm
    - Chest pain for more than two minutes after stopping exercise
    - Dyspnea for more than two minutes after stopping exercise
    - Vital signs out of range (systolic BP >= 250 mmHg or diastolic >= 115 mmHg, HR>120 beats/minute or < 45 beats/minute) for more than two minutes after stopping exercise
    - A fall during study recommended activity
    - A symptom or illness that developed and required medical management or attention

- Emergent adverse event of special interest
  - Emergency room or urgent care visit
  - Fracture
  - Outpatient surgery
  - Abnormal laboratory or diagnostic test result requiring immediate medical attention
  - Restricted activity due to health problem potentially related to the study activity that led an inability to leave home for at least one week

- All emergent adverse events by primary SOC, HLGT, HLT, and PT, showing number (%) of participants with at least 1 emergent adverse event and number of events and rate per patient-year sorted by the SOC internationally agreed order. The other levels (HLGT, HLT, PT) will be presented in alphabetical order
- All emergent adverse events by primary SOC and PT, showing the number (%) of participants with at least 1 emergent adverse event and number of events and rate per patient-year, sorted by the internationally agreed SOC order and by decreasing incidence of PTs within each SOC in the MCI group.

Analysis of all emergent serious adverse event(s)

- All emergent serious adverse, by primary SOC an PT for each subcategory, showing the number (%) of participants and number of events and rate per patient-year, sorted by the internationally agreed SOC order and by decreasing incidence of PTs within each SOC in the MCI group for each subcategory.

Analysis of emergent adverse events of special interest

- All emergent adverse events with special interest, by primary SOC an PT for each subcategory, showing the number (%) of participants and number of events and rate per patient-year, sorted by the internationally agreed SOC order and by decreasing incidence of PTs within each SOC in the MCI group for each subcategory.

Analysis of emergent adverse events occurred under the supervision of study-related personnel

- All EAE for AE occurred under the supervision of study-related personnel, by primary SOC and PT for each subcategory, showing the number (%) of participants and number of events and rate per patient-year, sorted by the internationally agreed SOC order and by decreasing incidence of PTs within each SOC in the MCI group for each subcategory.

Analysis of emergent adverse events unexpected related to study procedures

- All EAE for unexpected AE related to study procedures, by primary SOC and PT, showing the number (%) of participants and number of events and rate per

patient-year, sorted by the internationally agreed SOC order and by decreasing incidence of PTs within each SOC in the MCI group.

2.4.5.2 Falls

- Emergent injury falls by primary by primary SOC, HLT and PT, showing the number (%) of participants and number of events and rate per patient-year, sorted by the SOC internationally agreed order. The other levels (HLT, PT) will be presented in alphabetical order.

2.4.5.3 Deaths

- Emergent adverse events leading to death (fatal as an outcome on the adverse event case report form page as reported by the Investigator) by primary SOC , HLGT, HLT, and PT showing number (%) of participants and the number (rate per patient-year) of AEs sorted by internationally agreed SOC order, with HLGT, HLT, and PT presented in alphabetical order within each SOC .
- Time to death will be compared in the randomized population with a baseline SPPB score <8 (as assigned by randomization) within the intervention arm, using a two-sided Log-rank's test stratified by study site and gender. Hazard ratio (and 95% CI) between intervention groups will be estimated using a Cox proportional hazard model with the stratification factors (study site and gender) as covariates. The cumulative incidence functions for the 2 intervention arms will be summarized using Kaplan-Meier method and displayed graphically. The cumulative incidence rate over time at each scheduled time-points of the protocol together with appropriate 95% confidence interval will be provided.

2.4.5.4 Analyses of laboratory variables

Laboratory parameters will be grouped and summarized by biological function as described in Section 2.1.4.4. The summary statistics (including number, mean, median, SD, minimum and maximum) of all laboratory variables (values and changes from baseline) will be calculated for each visit or study assessment (baseline, each post baseline time point, last on-study) by intervention arm.

For baseline hematology and clinical chemistry parameters, the number and percentage of participants with normal value, out of range value, potentially medically important value and value requiring immediate notification, according to the thresholds defined in the protocol (list provided in Appendix C).

The total number and percentage of participants presenting at least one post-baseline abnormality as defined in the protocol will be presented by intervention arm, irrespective of the baseline level and according to the following baseline categories:

- Normal/missing
- Abnormality criteria as defined in the protocol

2.4.5.5 Analyses of vital sign variables

The summary statistics (including number, mean, median, standard deviation, minimum and maximum) of all vital signs variables (SBP, DBP, HR and changes from baseline) will be calculated for each study assessment (baseline, each post-baseline time point, last on-study) by intervention arm. For these parameters mean changes from baseline with the corresponding standard error will be plotted over time (at same time points) in intervention arm.

The total number and percentage of participants presenting at least one post-baseline abnormality in SBP, DBP or HR, as per the threshold defined in the protocol (list provided in Appendix B) will be presented by intervention arm, irrespective of the baseline level and according to the following baseline categories:

- Normal/missing
- Abnormality criteria as defined in the protocol

2.4.6 Analyses of pharmacokinetic and pharmacodynamic variables

Not applicable

2.4.7 Analyses of quality of life variables

The analysis of data from EQ-5D -5L instrument will be performed on all randomized participants with a baseline SPPB score <8 assigned by randomization, and on all randomized participants with a baseline SPPB score ≥8 assigned by randomization separately.

Baseline is defined as the randomization visit V1 (T0) evaluation. Analysis window will be used to assign time points (see Section 2.5.4).

The same MMRM model, as described in section 2.4.4.2 will be used to analyze the change from baseline in EQ-5D-5L index utility score, as well as the change from baseline in EQ-5D-5L VAS.

In addition, results per time point for all planned time points visits (value at visits using analysis windows, and changes from baseline) will be summarized and plotted using LS mean and SE for each intervention group obtained from the same MMRM models with raw values and changes from baseline as response variable in the model as appropriate. For weight, similar summary will be provided, but the percent change from baseline will be presented instead.

2.5 DATA HANDLING CONVENTIONS

2.5.1 General conventions

The following formulas will be used for computation of parameters.

Date of visit

The date of the anthropometry exam is considered as the date of visit. In case the date of anthropometry is missing, the following order of available dates is considered:

- Date of exam from the “400-meter walk test” e-CRF form
- Date of exam from the “ADL Proxy” e-CRF form
- Date of exam from the “Assistive Device Questionnaire” e-CRF form
- Date of exam from the “PAT-D” e-CRF form
- Date of exam from the “Outcome events” e-CRF form

Medical history

“Cardiovascular medical history” is defined using pre-listed items under the “Cardiovascular” subsection of the e-CRF page “Medical History”: if any of the pre-listed questions below is answered “Yes” then the participant is considered having a cardiovascular medical history:

- Has a doctor ever told you that you have high blood pressure or hypertension?

- Are you currently taking any medicine for your high blood pressure?
- Has a doctor ever told you that you had a heart attack, or coronary, or myocardial infarction and you had to be hospitalized overnight?
- Has a doctor ever told you that you had heart failure or congestive heart failure?
- Has a doctor ever told you that you had angina or chest pain due to blockage in the arteries of your heart?
- Have you had coronary artery bypass surgery ?
- Have you ever had a stent and/or angioplasty of the coronary arteries, which is a dilation of the arteries of the heart with a balloon?
- Have you ever had a carotid endarterectomy, which is surgery on the arteries in your neck to improve blood flow to your brain?
- Do you have a pacemaker?

“Diabetes medical history” is defined using pre-listed items under the “Diabetes” subsection of the e-CRF page “Medical History”: if any of the pre-listed questions below is answered “Yes” then the participant is considered having a diabetes medical history:

- Has a doctor ever told you that you had diabetes, sugar in your urine, or high blood sugar?
- Are you now using medication that you swallow to treat or control your diabetes?
- Are you now using insulin injections?

“Neurologic medical history” is defined using pre-listed items under the “Neurologic” subsection of the e-CRF page “Medical History”: if any of the pre-listed questions below is answered “Yes” then the participant is considered having a neurologic medical history:

- Has a doctor ever told you that you had a transient ischemic attack (TIA) or mini-stroke?
- Has a doctor ever told you that you had a stroke or brain hemorrhage and had to be hospitalized?

“Musculoskeletal medical history” is defined using pre-listed items under the “Musculoskeletal” subsection of the e-CRF page “Medical History”: if any of the pre-listed questions below is answered “Yes” then the participant is considered having a musculoskeletal medical history:

- Has a doctor ever told you that you had a broken or fractured hip and had to be hospitalized?
- Since the age of 50, have you ever been told by a doctor, nurse, therapist, or medical assistant that you had broken or fractured any other bones?

“Arthritis medical history” is defined using pre-listed items under the “Arthritis” subsection of the e-CRF page “Medical History”: if any of the pre-listed questions below is answered “Yes” then the participant is considered having an arthritis medical history:

- During the last 6 months, have you seen a doctor specifically for arthritis or rheumatism?
- Did you have pain and/or stiffness in any of the following joints? (Hands/Fingers, Shoulders, Knees, Hips, Back/spine, Foot)

Disease characteristics at baseline

The 400-m walk speed in m/s is the ratio of 400 divided by the time to walk 400 meters in seconds.

Rate of adverse events per patient-year

Computed as [sum of number of events for all analysis population] / [sum of patient-year of study participation for all analysis population], with patient-year of study participation calculated per participant as number of days from date of randomization visit V1-T0 (or date of randomization if V1 is missing) to end date of study participation as reported by investigators in the End of study e-CRF page divided by 365.25.

2.5.2 Data handling conventions for secondary efficacy variables

- The usual gait speed in m/s is the ratio of 4 meters walks divided by the time for the faster for the two walks in seconds (as reported in eCRF for the Gait Speed test component of SPPB).
- Changes in mood using the Center for Epidemiological Studies-Depression scale (CES-D): The score obtained with the 11-item version of the CES-D will be transformed using the procedure recommended by Kohout (3) to make it compatible with the full 20-item instrument, i.e.:
  - For males, (Z score*7.2)+7.1, where Z score is the familiar standardizing transformation
  - For females, (Z score*9.1)+10.0

2.5.3 Missing data

For categorical variables, participants with missing data are not included in calculations of percentages unless otherwise specified. When relevant, the number of participants with missing data is presented.

Handling of adverse events with missing or partial date/time of onset

Missing or partial adverse event onset dates will not be imputed. By default, if the partial adverse event onset date information does not indicate that the adverse event started prior to the randomization visits or after the end date of study participation as reported by investigators in the End of study e-CRF, the adverse event will be classified as emergent. No imputation of adverse event end dates will be performed.

Handling of laboratory and vital signs abnormalities

If a participant has a missing baseline he will be grouped in the category "normal/missing at baseline."

2.5.4 Windows for time points

Data analyzed by time point (including efficacy, biological data, vital signs, quality of life) will be summarized using the analysis windows given in Table 2.

Table 2 – Analysis windows definition

Time point	Targeted study day	Analysis window in study days
Month 3	91	61 to 137 (excluded)
Month 6	183	137 to 274 (excluded)
Month 12	365	274 to 457 (excluded)
Month 18	548	457 to 639 (excluded)
Month 24	730	639 to 822 (excluded)
Month 30	913	822 to 1004 (excluded)
Month 36	1095	1004 to 1187 (excluded)

Study days are calculated from the day of randomization visit V1-T0 (or day of randomization if V1 is missing); the day of randomization visit being Day 0.

For all post-baseline time points (including withdrawal visits), the value used for the analyses at a given time point (e.g. at month 24) is the valid value obtained within the corresponding analysis window. If multiple valid values of a variable exist within an analysis window, the nearest from the targeted study day will be selected. In case of equality, the value associated to the planned protocol visit that corresponds to the analysis visit (as per analysis windows) is selected if any, otherwise the earliest between the 2 assessments is selected.

2.5.5 Unscheduled visits

For efficacy, safety laboratory data, vital signs or quality of life, withdrawal visit measurements may be used to provide a measurement for a time point using analysis windows re-allocation (see Section 2.5.4), if appropriate according to their definitions. The measurements may also be used to determine abnormal values.

2.5.6 Pooling of centers for statistical analyses

There is no plan for pooling centers in the analyses.

3 INTERIM ANALYSIS

No interim analysis is planned.

4 DATABASE LOCK

The database is planned to be locked in Q4 2019/Q1 2020

5 SOFTWARE DOCUMENTATION

All summaries and statistical analyses will be generated using SAS version 9.4 or higher.

6 REFERENCES

1. Pahor M, Guralnik JM, Ambrosius WT et al. Effect of Structured Physical Activity on Prevention of Major Mobility Disability in Older Adults: The LIFE Study Randomized Clinical Trial. JAMA. 2014;311(23):2387-2396.

2. Fielding RA, Rejeski WJ, Blair S et al. The Lifestyle Interventions and Independence for Elders Study: Design and Methods. J Gerontol A Biol Sci Med Sci. 2011;66(11):1226-1237.

3. Kohout FJ, Berkman LF, Evans DA, Cornoni-Huntley J. Two shorter forms of the CES-D (Center for Epidemiological Studies Depression) depression symptoms index. J Aging Health. 1993; 5(2):179-1934.

4. Van Hout B, Janssen MF, et al. Interim scoring for the EQ-5D-5L: Mapping the EQ-5D-5L to EQ-5D-3L value sets. Value in Health. 2012 Jul-Aug; 15(5):708-15

7 LIST OF APPENDICES

Appendix A: Study flowchart

Appendix B: Vital signs abnormalities and alert

Appendix C: Blood test abnormalities

Appendix A Study flowchart

	**PS**	**S1**	**S2**	**V1**	**V2**	**V3**	**V4**	**V5**	**V6**	**V7**	**V8**	**V9**
Type of contact	*Phone*	*Visit*	*Visit*	*Visit*	*Visit*	*Visit*	*Visit*	*Visit*	*Visit*	*Visit*	*Visit*	*Visit*
Time (D=days, M=Months)	*-D60-T0*	*-D30-T0*	*-D30-T0*	*T0*	*M3*	*M6*	*M12*	*M18*	*M24*	*M30*	*M36*	*EoS*
Verbal consent	X											
First-contact interview	X											
Informed consent		X										
Medical history		X										
Physical exam		X										
Medication inventory		X			X	X	X	X	X	X	X	X**
SPPB		X			X	X	X	X	X	X	X	X**
MMSE		X					X		X		X	X**
ECG		X				X	X	X	X	X	X	X**
Blood pressure		X			X	X	X	X	X	X	X	X**
400-m walk test		X	X*		X	X	X	X	X	X	X	X**
SARC-F		X										
Anthropometry		X		X	X	X	X	X	X	X	X	X**
DXA		X	X*				X		X		X	X**
Study eligibility checklist				X								
Planning of follow-up		X		X	X	X	X	X	X	X	X	X**
Update contact information		X		X	X	X	X	X	X	X	X	X**
Randomisation				X								
Provision of ICT kit to the participant				X								
Blood drawn				X	X		X		X		X	X**
Urine sampling				X	X		X		X		X	X**
Sociodemographic characteristics				X								
Comorbidity				X			X		X		X	X**
CIRS				X			X		X		X	X**
Motivation questionnaire				X			X		X		X	X**
EuroQoL-5D				X	X	X	X	X	X	X	X	X**
Handgrip strength				X		X	X	X	X	X	X	X**
PAT-D				X	X	X	X	X	X	X	X	X**
TMT-A and TMT-B				X			X		X		X	X**
CES-D				X		X	X	X	X	X	X	X**
MNA-SF				X		X	X	X	X	X	X	X**
Self-reported PA questionnaire				X	X	X	X	X	X	X	X	X**
HE questionnaire				X	X	X	X	X	X	X	X	X**
Accelerometry (7-day Physical Activity report)				X		X	X	X	X	X	X	X**
Standard blood biochemical assessment				X			X		X		X	X**
Dietary assessment (3-day record, intervention only)				X			X		X		X	

Incident outcomes assessment					X	X	X	X	X	X	X	X**

SPPB: Short Physical Performance Battery; MMSE: Mini-Mental state Examination; ECG: electrocardiogram; SARC-F: SARC-F questionnaire: DXA: dual energy X-ray absorptiometry; ICT: Information and Communication Technology: CIRS: Cumulative Illness Rating Scale; PAT-D: Pepper Assessment Tool for Disability: TMT: Trial Making Test: CES-D: Centre for Epidemiological Studies-Depression scale; MNA: Mini-Nutritional Assessment-Short Form; PA: Physical activity; HE: Health economics

* The assessment may be conducted during a second screening visit

** If the participant decides to withdraw from the study an end of study visit (EoS) will be conducted. Every effort will be made in order to collect the largest amount of information about his/her health status. The assessment of the primary outcome will be prioritised. DXA scan will be reacquired if the previous examination has been performed more than 6 months earlier. All tests and questionnaires will be performed if previous assessments have conducted more than 3 months earlier

Appendix B Vital signs abnormalities and alert

ALERT	ACTION
Blood Pressure<br>SBP > 140mm/Hg or<br>DBP > 90mm/Hg	Clinic staff inform the participant
Blood Pressure<br>SBP > 170mm/Hg or<br>DBP > 100mm/Hg	Qualified staff should talk to participant, and encourage participant to seek additional follow-up and/or evaluation.
Resting Pulse<br>Rate > 100 or < 40 beats/min	Qualified staff should talk to participant, and encourage participant to seek additional follow-up and/or evaluation.

Appendix C Blood test abnormalities

Test	Value out of reference range	Value potentially medically significant	Value requiring immediate notification
Triglycerides (mg/dL)	>199		
Total cholesterol (mg/dL)	>199		
HDL (mg/dL)	<40		
Haemoglobin (g/dL)	M <13 or > 16<br>F <12 or > 15	M < 12 or > 16.5<br>F < 11 or > 16	< 8 or > 18
WBC count (/mm^3)	< 4000 or >11000	< 3000 or > 12000	< 2000 or > 20000
Platelet count (/mm^3)	< 130000 or > 400000	< 100000 or > 500000	< 30000 or > 1000000
Sodium (mEq/L)	< 135 or > 146	< 130 or > 155	< 125 or > 160
Potassium (mEq/L)	< 3.5 or > 5.3	< 3.0 or > 5.7	< 2.6 or > 6.2
Calcium (mEq/L)	<8.5 or > 10.3	< 8.0 or > 11.5	< 7.0 or > 13.0
Glucose (mg/dL)	< 70 or > 125	< 60 or > 140	< 50 or > 400
BUN (mg/dL)	> 30	> 40	> 80
Creatinine (mg/dL)	> 1.4	M >2.0<br>F > 1.6	> 3.5
Albumin (g/dL)	< 3.5	< 3.0	
Vitamin D (ng/mL)	< 30		

SPRINTT trial sites

Site name	City, country	Site coordinator	Enrolled participants
Università Cattolica del Sacro Cuore (coordinating centre)	Rome, Italy	Francesco Landi	202
Centre Hospitalier Universitaire de Limoges	Limoges, France	Achille Tchalla	61
Centre Hospitalier Universitaire de Toulouse	Toulouse, France	Bruno Vellas	104
Diabetes Frail, Medici Medical Practice	Luton, United Kingdom	Alan J. Sinclair	65
Friedrich-Alexander-Universität Erlangen-Nürnberg	Nurnberg, Germany	Cornel C. Sieber	123
Hospital Universitario de Getafe	Getafe, Spain	Leocadio Rodriguez-Mañas	117
Hospital Universitario Ramón y Cajal	Madrid, Spain	Alfonso J. Cruz-Jentoft	81
IRCCS INRCA	Ancona, Italy	Fabrizia Lattanzio	159
Lanspitali University Hospital §	Reykjavik, Iceland	Pálmi V. Jónsson	25
Medizinische Universität Graz §	Graz, Austria	Regina Roller-Wirnsberger	51
Silesian Hospital in Opava	Opava, Czech Republic	Ingrid Rýznarová	55
Università degli Studi di Parma §	Parma, Italy	Marcello Maggio	108
Universiteit Maastricht	Maastricht, The Netherlands	Annemie M. W. J. Schols	56
University of Helsinki	Helsinki, Finland	Timo Strandberg	142
Univerzita Karlova v Praze	Prague, Czech Republic	Eva Topinková	60
Uniwersytet Jagiellonski Collegium Medicum	Krakow, Poland	Anna Skalska	101
Aston University *	Birmingham, United Kingdom	Srikanth Bellary	9

* This site withdrew from the study because of administrative issues after approximately 5 months of trial beginning

§ These centres were engaged as backup sites to support participant recruitment as per the protocol's mitigation plan

SPRINTT consortium partners

Astellas Pharma Europe BV (Leiden, The Netherlands): Andeleeb Dahy, Makoto Kashiwa; *Bluecompanion Ltd.* (London, United Kingdom): Susanna Del Signore; *Boehringer Ingehleim International GmbH* (Ingelheim am Rhein, Germany): Laurent Nicolas, Joachim Scholpp; *Caretek S.r.l.* (Turin, Italy): Gianluca Zia (co-leader of the ICT Enabling Infrastructure and Operations work package), Cinzia Bertuzzi, Sabina De Giorgi, Luca Feletti, Alessandro Loria, Davide Mantovani, Elisa Marchioro, Francesco Mocci, Alberto Sacco, Maria Grazia Varesio; *Centre Hospitalier Universitaire de Limoges* (Limoges, France): Achille Tchalla (local principal investigator), Maxime Billot, Noëlle Cardinaud, Muriel Castelli, Marion Charenton-Blavignac, Cecilia Ciccolari-Micaldi, Thierry Dantoine, Caroline Gayot, Nicolas Giroult, Anael Larreur, Cécilie Laubarie-Mouret, Delphine Marchesseau, Thomas Mergans, Thai Binh Nguyen, Arnaud Papon, Johann Ribet, Isabelle Saulnie; *Centre Hospitalier Universitaire de Toulouse* (Toulouse, France): Bruno Vellas (local principal investigator and SPRINTT co-principal investigator for the academia), Gabor Abellan Van Kan, Virginie Biville, Lauréane Brigitte, Matteo Cesari (academic co-leader of the Clinical Consensus over Indication, Target Population and Clinical Trial Design for Data Generation work package), Carole Cervera, Céline Cluzan, Muriel Croizet, Sophie Dardenne, Marie Dorard, Charlotte Dupuy, Emilie Durand, Catherine Faisant, Sophie Guyonnet, Rémi Mauroux, Agathe Milhet, Sylvie Montel, Pierre-Jean Ousset, Cécile Picauron, Gaelle Soriano, Bernard Teysseyre; *Diabetes Frail Ltd.* (Droitwich Spa, United Kingdom): Alan Sinclair (local principal investigator), Sital Harris, Allison Ogborne, Sarah Ritchie, Harriet Sinclair, Lois Tirrell, Caroline Sinclair; *EU-Open S.r.l.* (Veronella, Italy): Alfredo Cesario (co-leader of the Regulatory Consensus work package), Barbara Cabin, Pim de Boer, Claire Ignaszewski, Ingrid Klingmann; *Friedrich-Alexander-Universität Erlangen-Nürnberg* (Nurnberg, Germany): Cornel C. Sieber (local principal investigator), Tina Auerswald, Christof Engel, Anna Franke, Ellen Freiberger, Ulrike Freiheit, Susann Gotthardt, Karin Kampe, Robert Kob, Christine Kokott, Carolin Kraska, Christian Meyer, Veronika Reith, Hanna Rempe, Daniel Schoene, Gabrielle Sieber, Kerstin Zielinski; *University of*

Goettingen Medical Center (Goettingen, Germany): Stefan D. Anker (academic leader of the Biomarkers Qualification work package), Nicole Ebner, Stephan von Haehling; *GlaxoSmithKline Research and Development Ltd.* (Brentford, United Kingdom): Michael Benecky; *Hospital Universitario de Getafe* (Getafe, Spain): Leocadio Rodriguez-Mañas (local principal investigator), Alejandro Alvarez-Bustos, Cristina Alonso Bouzon, Beatriz Contreras Escamez, Jimmy Gonzales Turin, Olga Laosa Zafra, Myriel Lopez Tatis, Laura Pedraza Sepulveda, Juan Luis Sanchez, Carlos Sanchez Puelles; *Hospital Universitario Ramón y Cajal – IRYCIS* (Madrid, Spain): Alfonso J. Cruz-Jentoft (local principal investigator and academic co-leader of the Clinical Study Implementation and Operations work package), Juan Álvarez-Santos, Belén Fernández-Jiménez, Jesús Mateos-del Nozal, Beatriz Montero-Errasquín, Beatriz Ponce-Moreno, Cristina Roldán-Plaza, Alfonso Romera-de Vicente, Vicente Sánchez-Cadenas, Carmen Sánchez-Castellano, Elisabet Sánchez-García, María Nieves Vaquero-Pinto; *INSERM/Université Toulouse III – Paul Sabatier UMR1027* (Toulouse, France): Sandrine Andrieu, Alessandro Blasimme, Cedric Dray, Emmanuelle Rial-Sebbag, Philippe Valet; *Institut de Recherches Internationales Servier IRIS* (Suresnes, France): Carmen Gorostiaga Ayestarán (EFPIA co-leader of the Clinical Study Implementation and Operations work package), Laurence Laigle, Itziar Martinez-Melchor, Belen Surroca; *IRCCS INRCA* (Ancona, Italy): Fabrizia Lattanzio (local principal investigator), Stefania Ambrosi, Renato Baldoni, Serena Bernabei, Anna Rita Bonfigli, Silvia Bustacchini, Barbara Carrieri, Antonio Cherubini (leader of the Stakeholder Information and Results Dissemination work package), Anna Rita Costantini, Michela Cucchi, Giuseppina Dell'Aquila, Emma Espinosa, Luciano Izzo, Massimiliano Fedecostante, Michela Mannoni, Antonella Mengarelli, Marino Modestino, Emanuele Monterubbianesi, Stefano Piomboni, Antonia Scrimieri, Eddy Severini, Fabiana Mirella Trotta, Lorella Vece, Susanna Venere, Elisa Zengarini; *Landspitali National Hospital and University of Iceland* (Reykjavik, Iceland): Pálmi V. Jónsson (local principal investigator), Milan Chang, Hrafnhildur Eymundsdóttir, Ólöf Guðný Geirsdóttir, Steinn Baugur Gunnarsson, Steinunn Guðnadóttir, Alfons Ramel, Konstantín Shcherbak; *Medizinische Universität Graz* (Graz, Austria):

Regina Roller-Wirnsberger (local principal investigator), Gerhard Wirnsberger; *Novartis Pharma AG* (Basel, Switzerland): Ronenn Roubenoff (Scientific Coordinator of SPRINTT and EFPIA co-leader of the Clinical Consensus over Indication, Target Population and Clinical Trial Design for Data Generation work package), Sheena Kao, Ram R. Miller (EFPIA co-leader of the Biomarkers Qualification work package), Romain Barnouin; *Roessingh Research and Development BV* (Enschede, The Netherlands): Lex van Velsenm, Miriam Vollenbroek-Hutten; *Sanofi–Aventis Recherche & Développement* (Chilly-Mazarin, France): Philippe Bordes (project coordinator and EFPIA co-leader of the Project Management and Oversight work package), Christian Asbrand, Raphael Bejuit (EFPIA co-leader of the Evaluation of Results work package), Sandrine Durand, Florence Joly (EFPIA co-leader of the Health Technology Assessment work package), Klaus Flechsenhar, Harmonie Goyeau, Régis Le Lain (EFPIA co-leader of the Regulatory Consensus work package), Jerome Mshihid, Aurèle Ndja (EFPIA co-leader of the ICT Enabling Infrastructure and Operations work package); *Silesian Hospital in Opava* (Opava, Czech Republic): Ingrid Rýznarová (local principal investigator), Ivana Drastichova, Eva Hasaliková, Radim Hucko, Seget Jakub, Monika Janácová, Michaela Kilmková, Martina Parízková, Kristyna Pavelková, Michaela Redrova, Petra Rusková; *Università Cattolica del Sacro Cuore* (Rome, Italy): Roberto Bernabei (SPRINTT principal investigator for the academia), Francesco Landi (local principal investigator and academic co-leader of the Clinical Study Implementation and Operations work package), Michele Basile, Damiano Biscotti, Claudio Boni, Vincenzo Brandi, Marianna Broccatelli, Riccardo Calvani, Carilia Celesti, Americo Cicchetti (academic co-leader of the Health Technology Assessment work package), Hélio Jose Coelho-Junior, Agnese Collamati, Silvia Coretti, Emanuela D'Angelo, Mariaelena D'Elia, Eugenio Di Brino, Giovanni Landi, Luca Mariotti (project manager and academic co-leader of the Project Management and Oversight work package), Anna Maria Martone, Emanuele Marzetti (academic co-leader of the Evaluation of Results work package), Elena Ortolani, Teodosio Pafundi, Cecilia Pantanelli, Anna Picca, Matteo Ruggeri, Filippo Rumi, Sara Salini, Giulia Savera, Elisabetta Serafini, Matteo Tosato, Davide L. Vetrano,

Fabio Vitale; *Università degli Studi di Firenze (Florence, Italy)*: Mauro Di Bari (academic co-leader of the Evaluation of Results work package); *Università degli Studi di Parma* (Parma, Italy): Marcello Maggio (local principal investigator), Elisa Adorni, Fulvio Lauretani, Yari Longobucco, Giovanna Maria Pelà, Sara Tagliaferri; *Université de Paris* (Paris France): Thomas Rapp (academic co-leader of the Health Technology Assessment work package), Yves Arrghi, Bastian Ravesteinj, Jérôme Ronchetti, Quittterie Roquebert, Jonathan Sicsic, Nicolas Sirven; *Universiteit Maastricht* (Maastricht, The Netherlands): Annemie M. W. J. Schols (local principal investigator), Harry Gosker, Jos M. G. A. Schols, Lisanne Schuurman, Nick Smeets, Coby van de Bool, Claire Weling; *University of Helsinki and Helsinki University Hospital* (Helsinki, Finland): Timo Strandberg (local pincipal investigator), Katja Hallikas, Marjatta Herranen, Laura Hyvönen, Kirsi Ikonen, Satu Jyväkorpi, Anne Karppi-Sjöblom, Kaisa Karvinen, Tarja Kindstedt, Saana Leirimaa, Hanna Öhman, Kaisu Pitkälä, Anja Punkka, Anna-Maria Saavalainen, Tuulia Salo, Katja Sohlberg, Reijo Tilvis, Annele Urtamo, Hannu Vanhanen; *Univerzita Karlova v Praze* (Prague, Czech Republic): Eva Topinková (local principal investigator), Lucie Bautzká, Tereza Gueye, Ilona Juklíčková, Pavla Mádlová, Helena Mejstříková, Helena Michálková, Eva Klára Novotná, Tereza Vágnerová; *Uniwersytet Jagiellonski Collegium Medicum* (Krakow, Poland): Anna Skalska (local principal investigator), Ewa Blaszczyk-Bebenek, Marcin Cwynar, Joanna Czesak, Paulina Fatyga, Malgorzata Fedyk-Lukasik, Tomasz Grodzicki, Paulina Jamrozik, Zbigniew Janusz, Ewa Klimek, Sylwia Komoniewska, Maria Kret, Maciej Ozog, Agnieszka Parnicka, Katarzyna Petitjean, Anna Pietrzyk, Karolina Piotrowicz, Barbara Skalska-Dulinska, Damian Starzyk, Katarzyna Szczerbinska, Borys Witkiewicz, Anna Wlodarczyk, Wieslawa Zgud

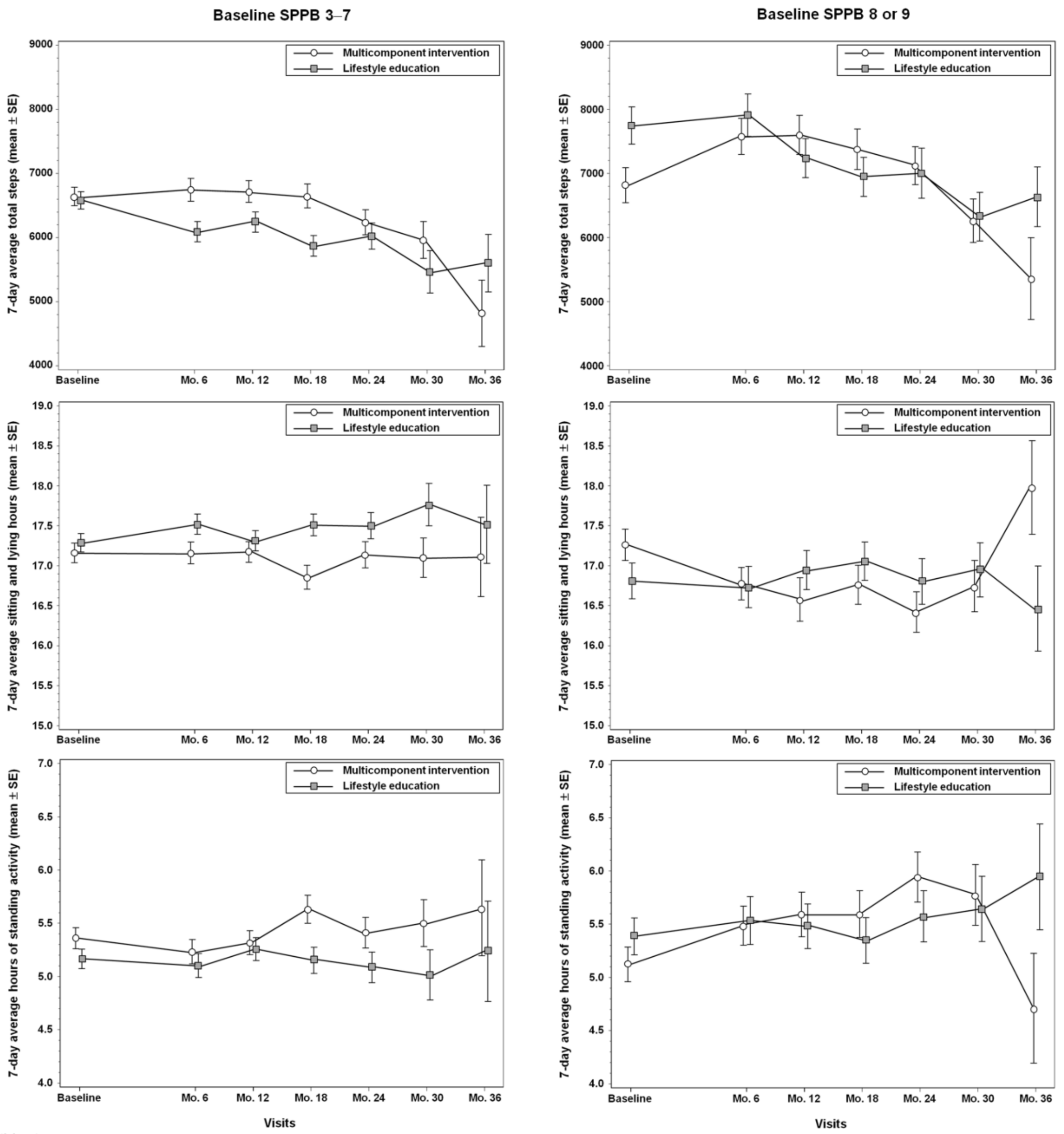

No. of participants	Baseline	Mo. 6	Mo. 12	Mo. 18	Mo. 24	Mo. 30	Mo. 36	Baseline	Mo. 6	Mo. 12	Mo. 18	Mo. 24	Mo. 30	Mo. 36
Multicomponent intervention	531	365	387	341	281	108	41	134	101	104	88	95	60	26
Lifestyle education	518	377	382	329	251	92	34	133	92	107	96	91	59	33

Fig S1 Seven day actimetry by intervention arm according to baseline short physical performance battery (SPPB) score category. SE=standard error

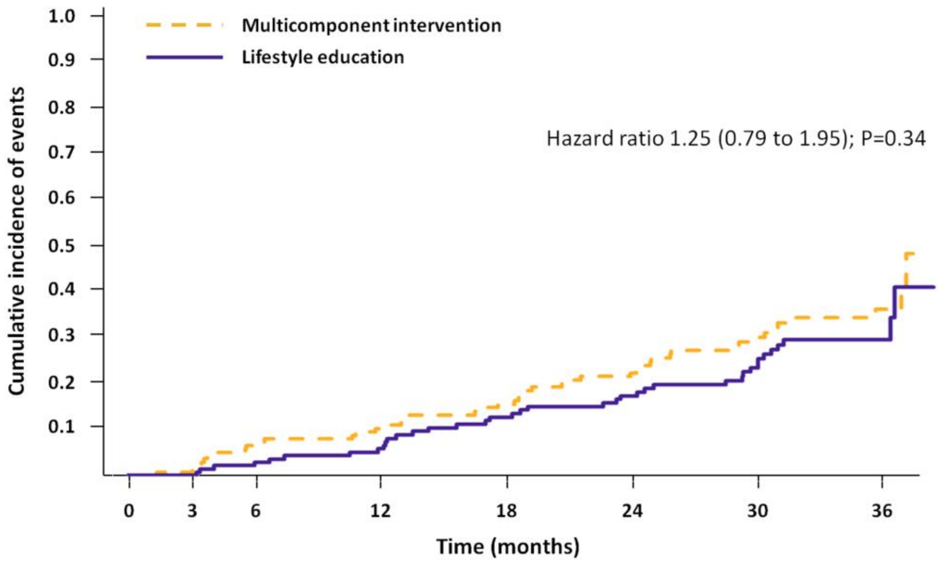

**No. at risk**								
Multicomponent intervention	155	139	131	121	113	100	69	22
Lifestyle education	159	147	135	127	114	103	77	15
**No. of events**								
Multicomponent intervention	0	1	9	14	21	31	39	44
Lifestyle education	0	0	4	8	17	23	33	37

Fig S2 Kaplan-Meier curves for incident mobility disability in participants with baseline short physical performance battery (SPPB) score of 8 or 9. The graph is truncated at 36 months, after which two additional mobility disability events were recorded in the multicomponent intervention group and one in the lifestyle education group. CI=confidence interval
