# Supplementary material for: Adherence to Physical Activity and Incident Mobility Disability in Older Adults With Mobility Limitations
Source: J Cachexia Sarcopenia Muscle. 2025 Jun 18;16(3):e13870. doi: 10.1002/jcsm.13870 (PMC12176070; doi:10.1002/jcsm.13870)
Supplement: Supplementary file 2 — Table S1 Probability of incident mobility disability according to baseline SPPB score category, removing deaths. Table S2. Effect of adherence to physical activity on changes (∆) in physical performance tests and appendicular lean mass according to baseline SPPB score category. Table S3. Effect of time on changes (∆) in physical performance tests and appendicular lean mass according to baseline SPPB score category. [file JCSM-16-e13870-s001.docx]

## **Supplementary Tables**

## **Supplementary Table 1.** Probability of incident mobility disability according to baseline SPPB score category, removing deaths.

| SPPB 3-7 | | | | | | | | | | | | |
| --- | --- | --- | --- | --- | --- | --- | --- | --- | --- | --- | --- | --- |
|  | **Model 1** | | | | | | **Model 2** | | | | | |
|  | **HR (95% CI)** | **P** | **HR (95% CI)** | **P** | **HR (95% CI)** | **P** | **HR (95% CI)** | **P** | **HR (95% CI)** | **P** | **HR (95% CI)** | **P** |
|  |  |  |  |  |  |  |  |  |  |  |  |  |
| Lifestyle education | Reference |  |  |  |  |  | Reference |  |  |  |  |  |
| Physical activity sessions |  |  |  |  |  |  |  |  |  |  |  |  |
| <2 weekly | 1.28 (1.06−1.55) | 0.011 | Reference |  |  |  | 1.14 (0.94−1.39) | 0.186 | Reference |  |  |  |
| 2-3 weekly | 0.58 (0.43−0.80) | 0.001 | 0.45 (0.33−0.63) | <0.001 | Reference |  | 0.57 (0.41−0.78) | 0.001 | 0.48 (0.34−0.69) | <0.001 | Reference |  |
| >3 weekly | 0.34 (0.25−0.48) | <0.001 | 0.26 (0.19−0.37) | <0.001 | 0.57 (0.37−0.88) | 0.010 | 0.33 (0.23−0.46) | <0.001 | 0.27 (0.18−0.38) | <0.001 | 0.50 (0.32−0.79) | 0.003 |
| Age |  |  |  |  |  |  | 1.06 (1.05−1.08) | <0.001 | 1.07 (1.05−1.10) | <0.001 | 1.09 (1.04−1.13) | <0.001 |
| Sex |  |  |  |  |  |  | 0.78 (0.64−0.95) | 0.014 | 0.75 (0.56−1.01) | 0.059 | 0.87 (0.51−1.48) | 0.608 |
| BMI |  |  |  |  |  |  | 1.02 (1.01−1.04) | 0.005 | 1.01 (0.98−1.04) | 0.475 | 1.03 (0.99−1.08) | 0.141 |
| Diabetes mellitus |  |  |  |  |  |  | 1.1 (0.89−1.35) | 0.389 | 1.13 (0.82−1.56) | 0.449 | 0.75 (0.42−1.33) | 0.323 |
| Cardiovascular disease |  |  |  |  |  |  | 1.23 (1.00−1.50) | 0.049 | 1.47 (1.06−2.03) | 0.022 | 1.77 (1.01−3.09) | 0.045 |
| Osteoarthritis |  |  |  |  |  |  | 0.99 (0.80−1.22) | 0.908 | 1.17 (0.84−1.64) | 0.360 | 1.48 (0.76−2.89) | 0.253 |
| Cancer |  |  |  |  |  |  | 0.98 (0.76−1.26) | 0.871 | 0.96 (0.65−1.40) | 0.825 | 1.15 (0.62−2.15) | 0.650 |
| SPPB score |  |  |  |  |  |  | 0.87 (0.80−0.94) | <0.001 | 0.84 (0.75−0.94) | 0.003 | 0.79 (0.63−0.98) | 0.036 |
| MMSE score |  |  |  |  |  |  | 1.00 (0.95−1.05) | 0.922 | 1.03 (0.96−1.12) | 0.368 | 1.07 (0.94−1.22) | 0.309 |
| CES-D score |  |  |  |  |  |  | 1.04 (1.01−1.06) | 0.002 | 1.00 (0.97−1.03) | 0.908 | 1.00 (0.95−1.05) | 0.996 |
| SARC-F score |  |  |  |  |  |  | 1.13 (1.07−1.19) | <0.001 | 1.12 (1.03−1.21) | 0.007 | 0.96 (0.84−1.10) | 0.528 |
| SPPB 8−9 | | | | | | | | | | | | |
|  | **Model 1** | | | | | | **Model 2** | | | | | |
|  | **HR (95% CI)** | **P** | **HR (95% CI)** | **P** | **HR (95% CI)** | **P** | **HR (95% CI)** | **P** | **HR (95% CI)** | **P** | **HR (95% CI)** | **P** |
|  |  |  |  |  |  |  |  |  |  |  |  |  |
| Lifestyle education | Reference |  |  |  |  |  | Reference |  |  |  |  |  |
| Physical activity sessions |  |  |  |  |  |  |  |  |  |  |  |  |
| <2 weekly | 2.21 (1.24−3.95) | 0.007 | Reference |  |  |  | 2.48 (1.27−4.86) | 0.008 | Reference |  |  |  |
| 2-3 weekly | 0.97 (0.48−1.95) | 0.928 | 0.45 (0.20−0.98) | 0.044 | Reference |  | 1.00 (0.49−2.05) | 0.996 | 0.32 (0.13−0.79) | 0.014 | Reference |  |
| >3 weekly | 0.57 (0.28−1.19) | 0.134 | 0.27 (0.12−0.60) | 0.001 | 0.62 (0.25−1.54) | 0.307 | 0.58 (0.27−1.21) | 0.147 | 0.19 (0.08−0.48) | <0.001 | 0.7 (0.26−1.86) | 0.477 |
| Age |  |  |  |  |  |  | 1.03 (0.98−1.08) | 0.206 | 0.97 (0.91−1.04) | 0.434 | 0.91 (0.81−1.02) | 0.109 |
| Sex |  |  |  |  |  |  | 0.56 (0.32−0.99) | 0.046 | 0.60 (0.26−1.40) | 0.238 | 0.4 (0.14−1.17) | 0.093 |
| BMI |  |  |  |  |  |  | 1.02 (0.98−1.07) | 0.324 | 1.00 (0.94−1.07) | 0.999 | 1.04 (0.94−1.14) | 0.468 |
| Diabetes mellitus |  |  |  |  |  |  | 1.44 (0.75−2.75) | 0.275 | 2.21 (0.81−6.04) | 0.121 | 2.68 (0.74−9.69) | 0.134 |
| Cardiovascular disease |  |  |  |  |  |  | 0.76 (0.43−1.33) | 0.331 | 0.54 (0.24−1.22) | 0.140 | 0.40 (0.13−1.23) | 0.108 |
| Osteoarthritis |  |  |  |  |  |  | 0.85 (0.47−1.54) | 0.598 | 0.73 (0.31−1.69) | 0.461 | 0.50 (0.16−1.58) | 0.238 |
| Cancer |  |  |  |  |  |  | 0.88 (0.45−1.72) | 0.711 | 0.63 (0.23−1.73) | 0.370 | 0.72 (0.15−3.39) | 0.677 |
| SPPB score |  |  |  |  |  |  | 0.93 (0.55−1.56) | 0.788 | 1.63 (0.78−3.37) | 0.191 | 1.02 (0.33−3.18) | 0.970 |
| MMSE score |  |  |  |  |  |  | 0.91 (0.80−1.03) | 0.149 | 0.96 (0.78−1.17) | 0.659 | 0.78 (0.55−1.10) | 0.155 |
| CES-D score |  |  |  |  |  |  | 1.06 (1.00−1.12) | 0.071 | 1.03 (0.94−1.12) | 0.563 | 0.97 (0.86−1.09) | 0.600 |
| SARC-F score |  |  |  |  |  |  | 1.13 (0.96−1.33) | 0.132 | 1.15 (0.92−1.45) | 0.225 | 1.20 (0.89−1.61) | 0.239 |

Model 1: unadjusted model. Model 2: adjusted for age, sex, BMI, medical conditions, SPPB, MMSE, CES-D, and SARC-F.

Abbreviations: Abbreviations: BMI=body mass index; CES-D=Center for Epidemiologic Studies Depression Scale; CI=confidence interval; HR=hazard ratio; MMSE=mini mental state examination; SARC-F=Strength, Assistance with walking, Rising from a chair, Climbing stairs and Falls; SPPB=short physical performance battery.

In participants with an SPPB score of 3 to 7, mobility disability occurred in 283/605 (46.8%) in the multicomponent intervention group (six deaths, 1.0%) and 316/600 (52.7%) in the lifestyle education group (seven deaths, 1.2%).

In participants with an SPPB score of 8 to 9, mobility disability occurred in 46/155 (29.7%) participants in the multicomponent intervention group (three deaths, 1.9%) and 38/159 (23.9%) in the lifestyle education group (two deaths, 1.3%).

## **Supplementary Table 2.** Effect of adherence to physical activity on changes (∆) in physical performance tests and appendicular lean mass according to baseline SPPB score category.

| SPPB 3−7 | | | | | | | | | | | | | |
| --- | --- | --- | --- | --- | --- | --- | --- | --- | --- | --- | --- | --- | --- |
|  |  | **SPPB score** | | **aLM (kg)** | | **aLM/BMI** | | **Handgrip strength (kg)** | | **Chair stand test (s)** | | **4 m gait speed (m/s)** | |
| Reference | **Compared category** | Mean diff. (95% CI) | P | Mean diff. (95% CI) | P | Mean diff. (95% CI) | P | Mean diff. (95% CI) | P | Mean diff. (95% CI) | P | Mean diff. (95% CI) | P |
| Lifestyle education | <2 sessions/week | 0.03 (−0.25, 0.31) | 1.000 | 0.03 (−0.53, 0.59) | 1.000 | −0.003 (−0.021, 0.014) | 1.000 | 0.85 (−0.42, 2.13) | 0.470 | 0.63 (−0.39, 1.65) | 0.621 | −0.22 (−0.49, 0.06) | 0.227 |
|  | 2−3 sessions/week | −0.64 (−1.02, −0.26) | <0.001 | 0.34 (−0.41, 1.10) | 1.000 | −0.004 (−0.027, 0.019) | 1.000 | 0.77 (−0.92, 2.46) | 1.000 | 0.42 (−0.96, 1.79) | 1.000 | 0.47 (0.10, 0.84) | 0.005 |
|  | >3 sessions/week | −1.38 (−1.72, −1.05) | <0.001 | 0.00 (−0.67, 0.67) | 1.000 | 0.012 (−0.009, 0.032) | 0.785 | −0.47 (−1.97, 1.03) | 1.000 | 2.90 (1.70, 4.09) | <0.001 | 0.73 (0.41, 1.05) | <0.001 |
| <2 sessions/week | Lifestyle education | −0.03 (−0.31, 0.25) | 1.000 | −0.03 (−0.59, 0.53) | 1.000 | 0.003 (−0.014, 0.021) | 1.000 | −0.85 (−2.13, 0.42) | 0.470 | −0.63 (−1.65, 0.39) | 0.621 | 0.22 (−0.06, 0.49) | 0.227 |
|  | 2−3 sessions/week | −0.67 (−1.04, −0.29) | <0.001 | 0.31 (−0.43, 1.05) | 0.937 | 0.000 (−0.023, 0.022) | 1.000 | −0.08 (−1.72, 1.56) | 1.000 | −0.21 (−1.51, 1.08) | 1.000 | 0.69 (0.33, 1.05) | <0.001 |
|  | >3 sessions/week | −1.41 (−1.75, -1.07) | <0.001 | −0.03 (−0.70, 0.64) | 1.000 | 0.015 (−0.005, 0.036) | 0.232 | −1.32 (−2.81, 0.17) | 0.103 | 2.27 (1.11, 3.42) | <0.001 | 0.95 (0.62, 1.27) | <0.001 |
| 2−3 sessions/week | HALE | 0.64 (−0.26, 1.02) | <0.001 | −0.34 (−1.10, 0.41) | 1.000 | 0.004 (−0.019, 0.027) | 1.000 | −0.77 (−2.46, 0.92) | 1.000 | −0.42 (−1.79, 0.96) | 1.000 | −0.47 (−0.84, −0.10) | 0.005 |
|  | <2 sessions/week | 0.67 (0.29, 1.04) | <0.001 | −0.31 (−1.05, 0.43) | 0.937 | 0.000 (−0.022, 0.023) | 1.000 | 0.08 (−1.56, 1.72) | 1.000 | 0.21 (−1.08, 1.51) | 1.000 | −0.69 (−1.05, −0.33) | <0.001 |
|  | >3 sessions/week | −0.74 (−1.15, −0.34) | <0.001 | −0.34 (−1.15, 0.47) | 0.940 | 0.015 (−0.009, 0.040) | 0.401 | −1.24 (−3.02, 0.55) | 0.289 | 2.48 (1.08, 3.88) | <0.001 | 0.26 (−0.13, 0.64) | 0.335 |
| >3 sessions/week | Lifestyle education | 1.38 (−1.05, 1.72) | <0.001 | 0.00 (−0.67, 0.67) | 1.000 | −0.012 (−0.032, 0.009) | 0.785 | 0.47 (−1.03, 1.97) | 1.000 | −2.90 (−4.09, −1.70) | <0.001 | −0.73 (−1.05, −0.41) | <0.001 |
|  | <2 sessions/week | 1.41 (1.07, 1.75) | <0.001 | 0.03 (−0.64, 0.70) | 1.000 | −0.015 (−0.036, 0.005) | 0.232 | 1.32 (−0.17, 2.81) | 0.103 | −2.27 (−3.42, −1.11) | <0.001 | −0.95 (−1.27, −0.62) | <0.001 |
|  | 2−3 sessions/week | 0.74 (0.34, 1.15) | <0.001 | 0.34 (−0.47, 1.15) | 0.940 | −0.015 (−0.040, 0.009) | 0.401 | 1.24 (−0.55, 3.02) | 0.289 | −2.48 (−3.88, −1.08) | <0.001 | −0.26 (−0.64, 0.13) | 0.335 |
| SPPB 8−9 | | | | | | | | | | | | | |
|  |  | **SPPB score** | | **aLM (kg)** | | **aLM/BMI** | | **Handgrip strength (kg)** | | **Chair stand test (s)** | | **4 m gait speed (m/s)** | |
| Reference | **Compared category** | Mean diff. (95% CI) | P | Mean diff. (95% CI) | P | Mean diff. (95% CI) | P | Mean diff. (95% CI) | P | Mean diff. (95% CI) | P | Mean diff. (95% CI) | P |
| Lifestyle education | <2 sessions/week | 0.00 (−0.48, 0.48) | 1.000 | 1.092 (−0.223, 2.408) | 0.170 | 0.004 (−0.033, 0.041) | 1.000 | 0.66 (−2.00, 3.33) | 1.000 | 0.47 (−1.19, 2.14) | 1.000 | −0.14 (−0.50, 0.22) | 1.000 |
|  | 2−3 sessions/week | 0.03 (−0.47, 0.54) | 1.000 | −0.013 (−1.341, 1.316) | 1.000 | 0.008 (−0.029, 0.046) | 1.000 | 0.12 (−2.57, 2.81) | 1.000 | 0.41 (−1.35, 2.17) | 1.000 | −0.16 (−0.53, 0.21) | 1.000 |
|  | >3 sessions/week | −0.66 (−1.09, −0.22) | <0.001 | 0.325 (−0.863, 1.513) | 1.000 | 0.007 (−0.027, 0.040) | 1.000 | −0.70 (−3.06, 1.67) | 1.000 | 2.07 (0.56, 3.58) | 0.002 | 0.32 (0.00, 0.64) | 0.047 |
| <2 sessions/week | Lifestyle education | 0.00 (−0.48, 0.48) | 1.000 | −1.092 (−2.408, 0.223) | 0.170 | −0.004 (−0.041, 0.033) | 1.000 | −0.66 (−3.33, 2.00) | 1.000 | −0.47 (−2.14, 1.19) | 1.000 | 0.14 (−0.22, 0.50) | 1.000 |
|  | 2−3 sessions/week | 0.03 (-0.58, 0.64) | 1.000 | −1.10 (−2.36, 0.15) | 0.105 | 0.004 (−0.041, 0.050) | 1.000 | −0.54 (−3.46, 2.37) | 1.000 | −0.06 (−1.84, 1.71) | 1.000 | −0.02 (−0.42, 0.37) | 1.000 |
|  | >3 sessions/week | −0.66 (−1.22, −0.10) | 0.014 | −0.77 (−1.94, 0.41) | 0.350 | 0.003 (−0.040, 0.045) | 1.000 | −1.36 (−4.05, 1.34) | 0.676 | 1.59 (−0.02, 3.21) | 0.054 | 0.46 (0.10, 0.82) | 0.007 |
| 2−3 sessions/week | Lifestyle education | −0.03 (−0.54, 0.47) | 1.000 | 0.013 (−1.316, 1.341) | 1.000 | −0.008 (−0.046, 0.029) | 1.000 | −0.12 (−2.81, 2.57) | 1.000 | −0.41 (−2.17, 1.35) | 1.000 | 0.16 (−0.21, 0.53) | 1.000 |
|  | <2 sessions/week | −0.03 (−0.64, 0.58) | 1.000 | 1.10 (−0.15, 2.36) | 0.105 | −0.004 (−0.050, 0.041) | 1.000 | 0.54 (−2.37, 3.46) | 1.000 | 0.06 (-1.71, 1.84) | 1.000 | 0.02 (−0.37, 0.42) | 1.000 |
|  | >3 sessions/week | −0.69 (−1.27, −0.12) | 0.012 | 0.34 (−0.84, 1.52) | 1.000 | −0.002 (−0.045, 0.041) | 1.000 | −0.82 (−3.53, 1.90) | 1.000 | 1.65 (−0.02, 3.33) | 0.054 | 0.48 (0.11, 0.86) | 0.006 |
| >3 sessions/week | Lifestyle education | 0.66 (0.22, 1.09) | <0.001 | −0.325 (−1.513, 0.863) | 1.000 | −0.007 (−0.040, 0.027) | 1.000 | 0.70 (−1.67, 3.06) | 1.000 | −2.07 (−3.58, −0.56) | 0.002 | −0.32 (−0.64, 0.00) | 0.047 |
|  | <2 sessions/week | 0.66 (0.10, 1.22) | 0.014 | 0.77 (−0.41, 1.94) | 0.350 | −0.003 (−0.045, 0.040) | 1.000 | 1.36 (−1.34, 4.05) | 0.676 | −1.59 (−3.21, 0.02) | 0.054 | −0.46 (−0.82, −0.10) | 0.007 |
|  | 2−3 sessions/week | 0.69 (0.12, 1.27) | 0.012 | −0.34 (−1.52, 0.84) | 1.000 | 0.002 (−0.041, 0.045) | 1.000 | 0.82 (−1.90, 3.53) | 1.000 | −1.65 (−3.33, 0.02) | 0.054 | −0.48 (−0.86, −0.11) | 0.006 |

Abbreviations: aLM=appendicular lean mass; BMI=body mass index; CI, confidence interval; SPPB=short physical performance battery.

## **Supplementary Table 3.** Effect of time on changes (∆) in physical performance tests and appendicular lean mass according to baseline SPPB score category.

| SPPB 3-7 | | | | | | | | | | | | | |
| --- | --- | --- | --- | --- | --- | --- | --- | --- | --- | --- | --- | --- | --- |
|  |  | **SPPB score** | | **aLM (kg)** | | **aLM/BMI** | | **Handgrip strength (kg)** | | **Chair stand test (s)** | | **4 m gait speed (m/s)** | |
| Reference | **Compared category** | Mean diff, (95% CI) | P | Mean diff, (95% CI) | P | Mean diff, (95% CI) | P | Mean diff, (95% CI) | P | Mean diff, (95% CI) | P | Mean diff, (95% CI) | P |
| Baseline | 24 months | −1.74 (−1.96, −1.51) | <0.001 | 0.58 (0.12, 1.04) | 0.007 | 0.015 (0.001, 0.029) | 0.031 | 1.014 (0.01, 2.01) | 0.046 | 3.02 (2.20, 3.83) | <0.001 | 0.78 (0.56, 1.00) | <0.001 |
|  | 36 months | −1.18 (−1.55, −0.80) | <0.001 | 1.13 (0.39, 1.87) | 0.001 | 0.048 (0.026, 0.070) | <0.001 | 2.96 (1.30, 4.61) | <0.001 | 3.98 (2.57, 5.39) | <0.001 | 0.27 (−0.10, 0.64) | 0.232 |
| 24 months | Baseline | 1.74 (1.51, 1.96) | <0.001 | −0.58 (−1.04, −0.12) | 0.007 | −0.015 (−0.029, −0.001) | 0.031 | −1.01 (−2.01, −0.01) | 0.046 | −3.02 (−3.83, −2.20) | <0.001 | −0.78 (−1.00, −0.56) | <0.001 |
|  | 36 months | 0.56 (0.17, 0.95) | 0.002 | 0.55 (−0.24, 1.33) | 0.290 | 0.033 (0.009, 0.057) | 0.003 | 1.94 (0.21, 3.67) | 0.022 | 0.96 (−0.52, 2.44) | 0.360 | −0.51 (−0.89, −0.12) | 0.005 |
| 36 months | Baseline | 1.18 (0.80, 1.55) | <0.001 | −1.13 (−1.87, −0.39) | 0.001 | −0.048 (−0.070, −0.026) | <0.001 | −2.96 (−4.61, −1.30) | <0.001 | −3.98 (−5.39, −2.57) | <0.001 | −0.27 (−0.64, 0.10) | 0.232 |
|  | 24 months | −0.56 (−0.95, −0.17) | 0.002 | −0.55 (−1.33, 0.24) | 0.290 | −0.033 (−0.057, −0.009) | 0.003 | −1.94 (−3.67, −0.21) | 0.022 | −0.96 (−2.44, 0.52) | 0.360 | 0.51 (0.12, 0.89) | 0.005 |
| SPPB 8−9 | | | | | | | | | | | | | |
|  |  | **SPPB score** | | **aLM (kg)** | | **aLM/BMI** | | **Handgrip strength (kg)** | | **Chair stand test (s)** | | **4 m gait speed (m/s)** | |
| Reference | **Compared category** | Mean diff, (95% CI) | P | Mean diff, (95% CI) | P | Mean diff, (95% CI) | P | Mean diff, (95% CI) | P | Mean diff, (95% CI) | P | Mean diff, (95% CI) | P |
| Baseline | 24 months | −0.82 (−1.15, −0.49) | <0.001 | 0.59 (−0.30, 1.49) | 0.332 | 0.011 (−0.014, 0.037) | 0.865 | 0.54 (−1.25, 2.33) | 1.000 | 5.08 (3.93, 6.22) | <0.001 | 0.38 (0.14, 0.62) | 0.001 |
|  | 36 months | −0.24 (−0.65, 0.17) | 0.464 | 1.05 (−0.06, 2.16) | 0.070 | 0.035 (0.004, 0.067) | 0.024 | 2.37 (0.18, 4.55) | 0.029 | 3.88 (2.45, 5.30) | <0.001 | −0.03 (−0.33, 0.27) | 1.000 |
| 24 months | Baseline | 0.82 (0.49, 1.15) | <0.001 | −0.59 (−1.49, 0.30) | 0.332 | −0.011 (−0.037, 0.014) | 0.865 | −0.54 (−2.33, 1.25) | 1.000 | −5.08 (−6.22, −3.93) | <0.001 | −0.38 (−0.62, −0.14) | 0.001 |
|  | 36 months | 0.58 (0.15, 1.00) | 0.004 | 0.46 (−0.72, 1.64) | 1.000 | 0.024 (−0.010, 0.057) | 0.265 | 1.83 (−0.45, 4.10) | 0.164 | −1.20 (−2.68, 0.28) | 0.157 | −0.41 (−0.72, −0.09) | 0.006 |
| 36 months | Baseline | 0.24 (−0.17, 0.65) | 0.464 | −1.05 (−2.16, 0.06) | 0.070 | −0.035 (−0.067, −0.004) | 0.024 | −2.37 (−4.55, −0.18) | 0.029 | −3.88 (−5.30, −2.45) | <0.001 | 0.03 (−0.27, 0.33) | 1.000 |
|  | 24 months | −0.58 (−1.00, −0.15) | 0.004 | −0.46 (−1.64, 0.72) | 1.000 | −0.024 (−0.057, 0.010) | 0.265 | −1.83 (−4.10, 0.45) | 0.164 | 1.20 (−0.28, 2.68) | 0.157 | 0.41 (0.09, 0.72) | 0.006 |

Abbreviations: aLM=appendicular lean mass; BMI=body mass index; CI, confidence interval; SPPB=short physical performance battery.
